# Positioning Creation of Cell Communication Channels via Chinese Microneedle

**DOI:** 10.34133/research.1459

**Published:** 2026-10-27

**Authors:** Yiwen Xu, Miaojie Fang, Zhenzhong Chen, Hong Liu, Yucheng Xue, Zilong Li, Yue Lin, Kelei Wang, Feng Lin, Ning Zhang

**Affiliations:** ^1^ Department of Orthopedic Surgery, The Second Affiliated Hospital, Zhejiang University School of Medicine, Hangzhou, Zhejiang, China.; ^2^ Orthopedics Research Institute, Zhejiang University, Hangzhou, Zhejiang, China.; ^3^ Key Laboratory of Motor System Disease Research and Precision Therapy of Zhejiang Province, Hangzhou, Zhejiang, China.; ^4^ Zhejiang Provincial Clinical Medical Research Center for Motor System Diseases, Hangzhou, Zhejiang, China.; ^5^ International Chinese Musculoskeletal Research Society, Hangzhou, Zhejiang, China.; ^6^ State Key Laboratory of Transvascular Implantation Devices, Hangzhou, Zhejiang, China.

## Abstract

The core dilemma of senescence mitigation lies in the disrupted and even detrimental communication between senescent cells and local immune cells. Existing delivery methods cannot precisely establish protective intercellular communication channels across cell types within the enclosed lesion cavity. In this study, we utilize Chinese microneedle technology for precise, minimally invasive localization of aged lesions, and to release spatially targeted and temporally sustained dual-field to create intercellular communication channels between macrophages and senescent cells. The unique physical properties of this Chinese microneedle system confine the current path to specific spatiotemporal windows, releasing an electro-field while simultaneously activating dynamic van der Waals forces that release functionalized hydrogel microparticles, thereby superimposing a vita-field. Within the dual-field active zone, the electro-field not only attenuates cellular senescence but also forms a communication channel from senescent cells to macrophages, driving macrophages into a hyperactivated state. Meanwhile, the vita-field stabilizes the macrophage membrane, enabling long-term, stable communication between hyperactivated macrophages and senescent cells. Ultimately, the intercellular communication channels established by this Chinese microneedle system alleviate cellular senescence and apoptosis, supporting the repair of aged lesions.

## Introduction

In multicellular organisms, tissue repair and regeneration are tightly regulated processes that rely on coordinated interactions among multiple cell types [1]. Among these, bidirectional communication between immune cells and resident somatic cells serves as a central regulatory system for these processes [2]. Beyond traditional inflammation-centered views, immune cells undergo dynamic functional-state transitions during repair and build complex signaling networks with somatic cells, involving not only cytokines and chemokines but also small-molecule metabolites that guide cell-fate transitions, migration, and tissue remodeling [3,4]. Macrophages, as a key immune population, have been shown to coordinate tissue repair through dynamic interactions with epithelial cells, endothelial cells, fibroblasts, and stem cells [5–7]. A bidirectional feedback loop exists between macrophages and mesenchymal stem cells [8], wherein macrophages drive a pro-regenerative phenotype, while activated stem cells reinforce the local anti-inflammatory microenvironment [9]. During cardiac and nervous system repair, macrophages promote angiogenesis and axonal regeneration through vascular endothelial growth factor-A (VEGF-A) and other signals [10,11]; in liver and skeletal muscle, they release Wnt3a and interleukin-10 (IL-10) to modulate progenitor cell differentiation [12,13]. These studies collectively establish macrophages as central regulatory hubs that orchestrate tissue regeneration through intercellular communication.

In pathological settings such as intervertebral disc degeneration (IVDD), this macrophage-dominated communication network becomes markedly dysregulated [14], creating a major barrier to tissue repair. Persistent inflammation, abnormal mechanical loading, and hypoxia in the degenerated disc collectively induce apoptosis and senescence of nucleus pulposus (NP) cells [15], leading to extensive loss of extracellular matrix (ECM) and fibrotic remodeling of the annulus fibrosus [16]. Current therapeutic strategies, which rely largely on broad modulation of signaling pathways, cannot overcome the disc’s unique avascular structure and dense annular barrier to achieve spatially precise intervention. The central challenge lies in the disrupted communication between senescent NP cells and local immune cells within this confined, immune-privileged niche. Existing delivery methods cannot reliably establish protective intercellular communication channels across cell types in this enclosed lesion. Therefore, how to breach the disc’s physical and biological barriers and precisely rewire beneficial intercellular communication remains a key scientific challenge for addressing IVDD.

To address this challenge, we propose a spatially precise therapeutic paradigm based on a Chinese microneedle system. This miniaturized device contains helical grooves at its tip that physically retain functionalized hydrogel microspheres via a thermosensitive adhesive [17], preventing premature loss during tissue penetration and enabling localized deposition within the avascular NP compartment. The hyaluronic acid methacrylate (HAMA) hydrogel microspheres, loaded with piezoelectric poly(l-lactic acid) (PLLA) and glycine-encapsulated liposomes, were fabricated by 3-dimensional (3D) printing. Under ultrasound stimulation, the piezoelectric PLLA component generates a localized electro-field, defined here as a noninvasive electrical field that directly modulates cellular activity [18]. Concurrently, the HAMA microspheres enable controlled release of glycine, establishing a vita-field, defined as a glycine-mediated cytoprotective biochemical microenvironment that preserves membrane integrity. The spatiotemporal coupling of these 2 fields constitutes the dual-field system, achieving synergistic electrical stimulation (ES) and metabolic protection.

In this dual-field system, the electro-field initiates the therapeutic cascade by reprogramming NP cell metabolism and inducing the secretion of a cocktail of immunomodulatory metabolite, while the vita-field serves as a protective niche that sustains macrophage survival following gasdermin D (GSDMD) pore formation, thereby enabling a persistent feedback loop back to NP cells.

We reasoned that this spatially precise delivery of the dual-field system would achieve 3 interrelated therapeutic effects. First, the electro-field directly improves mitochondrial function and reduces senescence markers in NP cells [19–21]. Second, electro-field-stimulated NP cells secrete a cocktail of specific metabolites, including leucine, proline, lysine, carnitine, *N*-acetyl-l-methionine, *N*-acetyl-d-glucosamine, and resveratrol (putatively annotated by accurate mass and mass spectrometry (MS)/MS spectral matching to the in-house database; Metabolomics Standards Initiative (MSI) level 2), that drives infiltrating macrophages toward a non-pyroptotic hyperactivated state via GSDMD pore formation, thereby inducing secretion of reparative cytokines (e.g., IL-10 and transforming growth factor-β (TGF-β)). Third, the vita-field stabilizes macrophage plasma membrane integrity, enabling these hyperactivated macrophages to sustain prolonged communication with senescent NP cells. Among the factors secreted by hyperactivated macrophages, we identified a morroniside-like metabolite as a key feedback metabolite that attenuates reactive oxygen species (ROS)-driven senescence signaling in NP cells via the Hippo-YAP/TAZ pathway, further reinforcing senescence mitigation.

The core innovation of this study is the spatially precise construction of a bidirectional, self-sustaining intercellular communication loop between NP cells and macrophages in the degenerated disc. By co-delivering the electro-field and the vita-field through the microneedle-microsphere system, we establish a dual-field microenvironment that (a) directly restores NP cells, (b) triggers NP-to-macrophage signaling through a specific metabolite cocktail, (c) promotes macrophages toward a non-pyroptotic hyperactivated state via GSDMD pores, with glycine providing membrane protection, and (d) creates a feedback loop mediated by morroniside-like metabolites that reinforces NP rejuvenation. This work thus introduces a dual-field, metabolite-mediated bidirectional communication network that achieves multicellular therapeutic effects on disc degeneration. By combining biophysical stimulation with immunometabolic regulation, we propose an “electro-immunometabolic axis” as a new paradigm for intervertebral disc regeneration, which provides a platform for deep-tissue repair and opens new avenues for next-generation regenerative therapies [22] (Fig. 1).

**Fig. 1.**
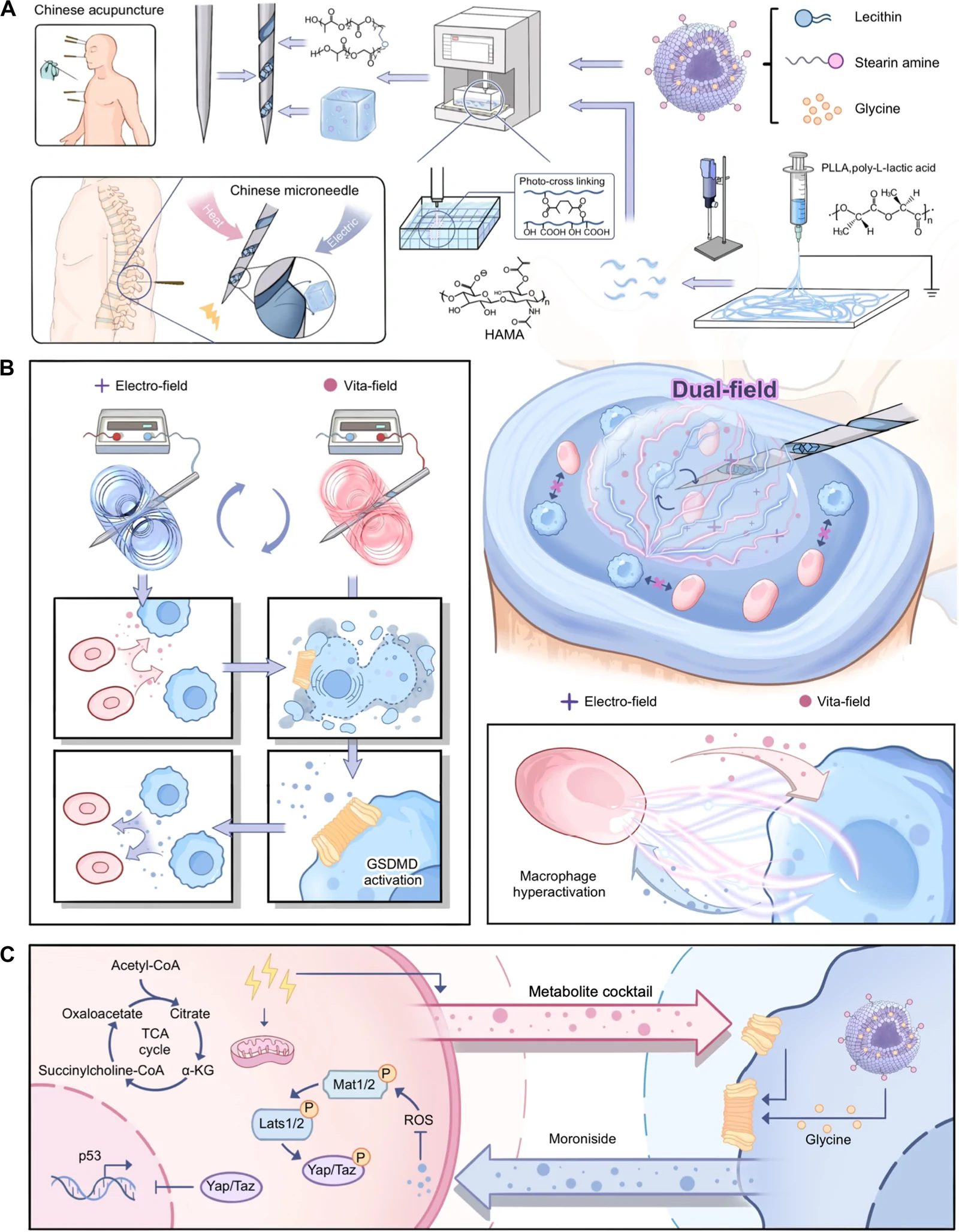
Dual-field creation of a metabolite-mediated cell-communication channel for disc rejuvenation. (A) Piezoelectric PLLA and glycine-loaded liposomes were integrated into composite microspheres via 3D printing using UV-assisted layer-by-layer curing. The microspheres were then loaded into the threaded grooves of a conductive Chinese microneedle using a thermosensitive gel (viscous at room temperature, flowable upon heating), enabling minimally invasive and spatially precise delivery into the degenerated intervertebral disc. (B) The microneedle generates a localized electro-field that rejuvenates senescent NP cells. These rejuvenated NP cells secrete a cocktail of specific metabolites (including leucine, proline, lysine, carnitine, *N*-acetyl-l-methionine, *N*-acetyl-d-glucosamine, and resveratrol), which stimulates non-pyroptotic GSDMD pore formation in infiltrating macrophages, triggering the secretion of reparative factors. Concurrently, glycine released from the liposomes creates a protective vita-field that maintains macrophage viability and function after GSDMD pore formation, sustaining a hyperactivated state. Among the secreted factors, a morroniside-like metabolite is a key feedback metabolite. (C) Morroniside (authentic standard) acts on senescent NP cells to suppress ROS-driven senescence signaling through the Hippo-YAP/TAZ pathway, thereby further alleviating NP cell senescence and apoptosis. Together, the electro-field and the vita-field establish a bidirectional, self-sustaining metabolic dialogue that reverses disc aging and restores tissue homeostasis.

## Results

### Fabrication and characterization of electrically responsive hydrogel-PLLA microspheres

We constructed an integrated physicochemical therapeutic platform through integration of piezoelectric PLLA films and HAMA microsphereliposome delivery systems into microneedle patches (Fig. 2A). Under ultrasound stimulation, PLLA films produce localized electrical signals to modulate cellular activity, while HAMA microspheres enable controlled release of encapsulated liposomes, thereby creating a spatiotemporally coupled “ES-drug delivery” modality for synergistic regulation.

**Fig. 2.**
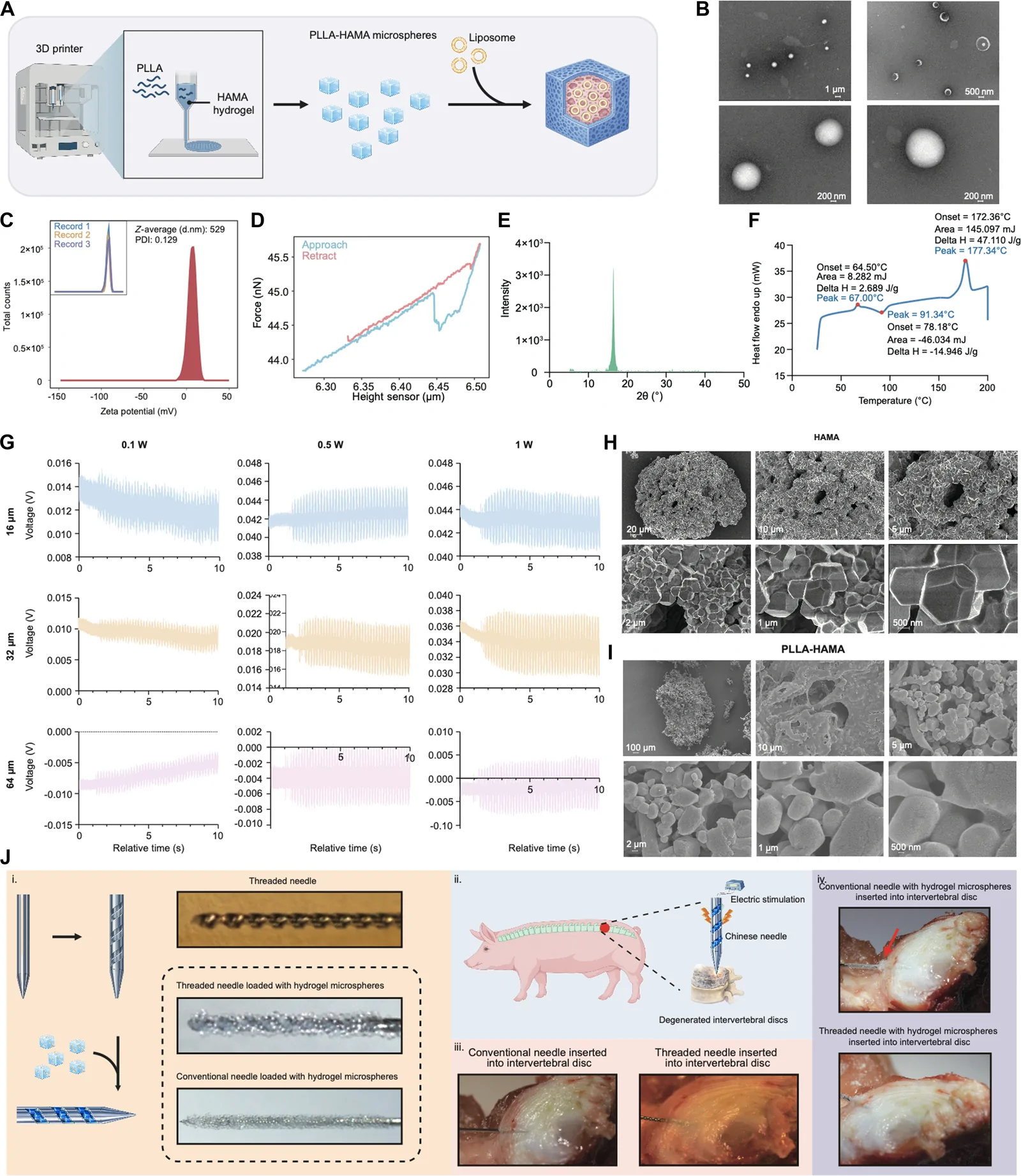
Construction and functional characterization of the integrated microneedle-HAMA microsphere-PLLA platform. (A) The system is constructed using a 3D printer to fabricate composite microspheres composed of PLLA and HAMA hydrogel. These PLLA-HAMA microspheres are subsequently loaded with liposomes carrying bioactive molecules. For therapeutic application, the composite microspheres are delivered into degenerated intervertebral discs via minimally invasive needle injection. Under ultrasound or mechanical stimulation, the PLLA component generates localized electrical signals, while the HAMA hydrogel enables sustained release of encapsulated liposomes, thereby achieving synergistic physicochemical regulation to promote disc regeneration. (B) TEM images of liposomes. Following negative staining with 2% (w/v) uranyl acetate, liposomes exhibited typical spherical vesicle morphology with uniform size distribution, good dispersity, and clearly visible phospholipid bilayer structures. (C) DLS analysis of liposome size distribution. The liposomes showed a *Z*-average diameter of 529 nm and a PDI of 0.129, indicating excellent size uniformity and colloidal stability (*n* = 3). (D) AFM force-distance curves of PLLA films. Red curve: approach; blue curve: retraction. Hertz model fitting yielded an average Young’s modulus of 1.66 GPa (*n* = 3). (E) XRD pattern of PLLA films. A distinct diffraction peaks at ~16.5° and 19.0° confirmed the presence of α-crystalline domains, indicating good crystallinity. (F) DSC curve of PLLA films (heating rate: 10 °C/min). (G) Piezoelectric voltage outputs of PLLA films with different thicknesses (16, 32, and 64 μm) under ultrasound stimulation at varying power levels (0.1, 0.5, and 1 W) (*n* = 3). (H) SEM images of 3D-printed HAMA microspheres at different magnifications. (I) SEM images of 3D-printed PLLA-HAMA composite microspheres. (J) Comparison of threaded versus conventional needle for microsphere delivery. (i) Threaded needle with helical grooves: The groove structure enables secure entrapment of hydrogel microspheres. (ii to iv) In a porcine intervertebral disc model, the threaded needle exhibits distinct structural and functional advantages: The helical grooves allow stable loading of hydrogel microspheres along the needle shaft, retain them during insertion, and enable precise release into the disc space. In contrast, the conventional needle lacks groove protection, resulting in substantial extrusion of microspheres during insertion and failed delivery (red arrows).

As bioactive-molecule carriers, the liposomes appeared as spherical vesicles with a uniform size distribution and clear bilayer structures in transmission electron microscopy (TEM) images after 2% (w/v) uranyl acetate negative staining (Fig. 2B). Dynamic light scattering (DLS) confirmed a *Z*-average diameter of 529 nm and a polydispersity index (PDI) of 0.129 (Fig. 2C), indicating excellent size uniformity and colloidal stability and thus supporting their use as effective vehicles for molecular loading.

The physical regulatory function of the platform depends on the piezoelectric effect of PLLA. We evaluated the mechanical properties of PLLA films (chemical structure and macroscopic morphology are shown in Fig. S1A). Atomic force microscopy (AFM) force-distance curves showed pronounced hysteresis between approach and retraction (Fig. 2D), indicating viscoelasticity and energy dissipation during nanoindentation. Hertz model fitting yielded an average Young’s modulus of 1.66 GPa (Fig. S1B), consistent with stable piezoelectric output under cyclic stress. X-ray diffraction (XRD) analysis revealed characteristic peaks at ~16.5° and 19.0° (Fig. 2E), corresponding to the α-crystalline phase of PLLA, confirming high crystallinity as the structural basis for piezoelectricity. Differential scanning calorimetry (DSC) further showed glass transition, cold crystallization, and melting peaks during heating at 10 °C/min (Fig. 2F), consistent with a semicrystalline structure. We then systematically assessed the piezoelectric output of PLLA films. Under ultrasound stimulation, films of varying thicknesses (16, 32, and 64 μm) generated stable voltage outputs, which increased with ultrasound power (0.1, 0.5, and 1 W) (Fig. 2G), demonstrating power- and thickness-dependent piezoelectric responses that enable tunable ES.

HAMA served as a sustained-release matrix for liposomes. Its chemical structure is shown in Fig. S1C, and the synthesis route is shown in Fig. S1D. ^1^H nuclear magnetic resonance (NMR) verified successful grafting of methacrylate groups onto hyaluronic acid chains (Fig. S1E), providing photo-crosslinking capability. Using 3D printing, we generated well-defined HAMA microspheres. Scanning electron microscopy (SEM) revealed a uniform spherical morphology with porous surfaces (Fig. 2H). Size distribution analysis of 280 microspheres revealed D10, D50, and D90 values of 5.52, 7.50, and 11.33 μm, respectively, with a span of 0.77, confirming size uniformity (Fig. S1F). After being coated with PLLA piezoelectric layers, the composite microspheres retained their spherical morphology with uniformly distributed PLLA shells (Fig. 2I), forming a core-shell structure that supports ultrasound-responsive ES and cargo release for synergistic therapy. To characterize the release behavior of the liposome-encapsulated glycine, we measured cumulative release in phosphate-buffered saline (PBS, pH 7.4, 37 °C) over 21 d. The release profile exhibited a typical biphasic pattern: an initial moderate release phase within the first 12 h (~18% cumulative release), followed by a sustained release phase extending up to 21 d, reaching approximately 90% cumulative release at day 21 (Fig. S1G). Three parallel samples showed consistent release behavior with minor variations, confirming the reproducibility of the release profile. These data demonstrate that the HAMA microsphere system provides sustained glycine release over an extended period. To enable minimally invasive and spatially controlled administration of the composite microspheres into the degenerated disc, we designed a Chinese microneedle. Compared with conventional smooth-surface needles, the threaded needle contained helical grooves that physically retained the hydrogel microspheres, preventing premature loss during tissue penetration (Fig. 2J (i)). Upon insertion into the intervertebral disc, the threaded needle enabled uniform deposition of microspheres along the thread channels, whereas conventional needles produced uneven distribution and substantial loss upon withdrawal (Fig. 2J (ii to iv)). This threaded design therefore provides a robust platform for precise and stable delivery of our piezoelectric PLLA-HAMA microspheres and glycine-loaded liposomes into the avascular NP, enabling subsequent generation of the electro-field and vita-field for establishment of cell-communication channels.

Finally, the maximum working concentration of functionalized hydrogel microspheres was cocultured with macrophages and NP cells to assess their biocompatibility. Live/dead staining at different time points showed that the microspheres did not cause evident cytotoxicity in either cell type at the maximum working concentration. At 24 h, both macrophages and NP cells showed robust adhesion with predominantly green live-cell signals. As the culture duration increased to 72 and 120 h, cell numbers continued to increase, and no obvious accumulation of red dead-cell signals was observed (Fig. S1H). Quantitative analysis showed that the proportion of live cells remained consistently high across all time points, with no statistically marked differences among groups (Fig. S1I). In line with these observations, Cell Counting Kit-8 (CCK-8) assays further confirmed the favorable biocompatibility of the microspheres (Fig. S1J), indicating that the material did not inhibit cell proliferation at the maximum working concentration.

### ES reprograms the transcriptional landscape and signaling networks of senescent NP cells

To generate an in vitro senescence model, NP cells were serially passaged, and d-galactose (8 mg/ml) was applied to accelerate metabolic stress and functional decline. After repeated “culture-passage” cycles, we generated a stable population of senescent NP cells (Fig. S2A). Cells were divided into 3 groups: Young, Aging, and ES. After treatment, cells were collected for bulk RNA sequencing (RNA-seq), and culture supernatants were collected for metabolomic analysis.

Transcriptomic analysis revealed that senescent NP cells exhibited pronounced up-regulation of multiple inflammation- and matrix degradation-related genes, including *Abcg1*, *Cd74*, *Il11*, *Il1a*, *Mmp3*, and *Selplg*, reflecting aberrant transcriptional activity under aging conditions (Fig. S2B). In contrast, ES substantially reprogrammed the transcriptional landscape: RNA-seq identified 224 genes significantly up-regulated in the ES group, whereas 411 genes were up-regulated in the Aging group (Fig. S2C). These transcriptional differences converged on functional pathways—ES promoted cell cycle progression, DNA replication, and oxidative phosphorylation while attenuating the senescence-associated secretory phenotype (SASP), inflammatory responses, and TGF-β signaling (Fig. S2D and E). Kyoto Encyclopedia of Genes and Genomes (KEGG) analysis further confirmed that ES favored pathways associated with cellular processes and metabolism, whereas Aging samples were enriched for immune and inflammatory signaling (Fig. S2F).

To resolve cellular heterogeneity within NP tissue, we performed single-cell RNA-seq (scRNA-seq) on rat caudal intervertebral discs. After quality filtering, 50,155 cells were retained for clustering into 19 distinct cell types (Fig. 3A and Fig. S3A and B). We then extracted NP-related populations and classified these cells into 6 subgroups based on molecular signatures (Fig. 3B and Fig. S3C): C01: stress/inflammatory NP cells (*Il23r^+^*); C02: anabolic NP chondrocytes; C03: Wnt-inhibitory NP-MSCs; C04: NP-edge MSCs (*Pi16^+^*); C05: proliferative/cycling NP-derived cells; C06: epithelial-like NP cells (Brachyury^+^). Dot plot analysis verified distinct marker expression patterns: C01 highly expressed Il23r; C02 was enriched for cartilage matrix genes (*Col9a1*, *Col9a3*); C03 expressed Wnt inhibitors (*Sfrp*, *Dkk2*); and C05 showed strong expression of cell cycle genes (*Top2a*, *Mki67*). The distribution of NP subgroups varied across conditions: Young samples were enriched in C01 and C02; Aging samples showed lower proportions of these clusters with modest increases in C03 and C05; ES treatment further reduced C01 and C02 while markedly enriching C04 and C06. These shifts indicate that ES promotes a transition from inflammatory/anabolic states toward stem/progenitor-like and epithelial-like states.

**Fig. 3.**
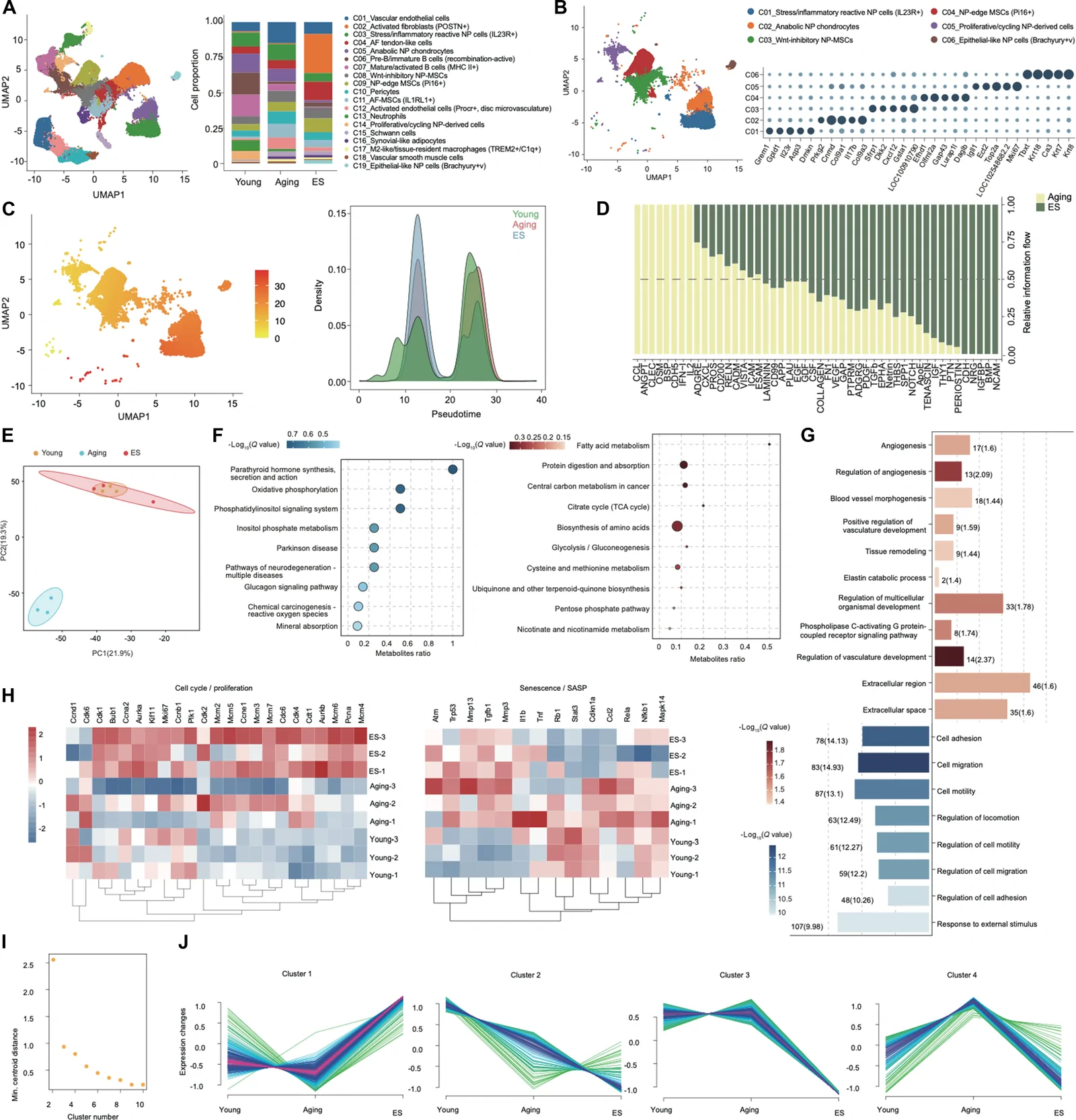
ES via microspheres restores transcriptional landscape and signaling networks of senescent NP cells. (A) UMAP visualization of integrated NP cell clusters and changes in cell type proportions across conditions. (B) Left, UMAP of NP subclusters; right, dot plots showing relative expression of marker genes. (C) Left, pseudotime trajectory analysis of NP cell differentiation. Right, density plots of pseudotime values across groups. (D) Comparison of signaling pathway activity between Aging and ES groups. CellChat rankNet analysis highlights pathways enriched in Aging (light green) versus ES (dark green). (E) PCA of metabolomic data showing clear separation among Young, Aging, and ES groups. (F) Pathway enrichment analysis comparing Aging and ES groups. Left, pathways down-regulated in ES relative to Aging (blue); right, pathways up-regulated in ES relative to Aging (red). (G) Bar plot showing enriched KEGG pathways. Left (blue), pathways down-regulated in ES relative to Aging; right (red), pathways up-regulated in ES relative to Aging. (H) Heatmap showing gene expression changes in NP tissue across 3 conditions (Young, Aging, ES). Left, cell cycle/proliferation-related genes; right, senescence/SASP-associated genes. (I) Mfuzz clustering analysis of metabolomic data in Young, Aging, and ES groups. Elbow plot showing the optimal number of clusters determined by Mfuzz analysis. (J) Expression trajectories of representative gene clusters across Young, Aging, and ES groups, illustrating distinct dynamic patterns under ES.

We then reconstructed pseudotime trajectories to define NP cell differentiation dynamics. The analysis revealed a continuous hierarchy that originated from proliferative NP-derived cells and extended toward terminally differentiated states (Fig. 3C, left). In agreement with CytoTRACE stemness scores (Fig. S3D), early pseudotime positions represented progenitor-like populations with high regenerative potential, whereas late pseudotime states reflected more differentiated or senescent cells. Density plots further illustrated condition-specific differences (Fig. 3C, right): Aging cells were broadly distributed and shifted toward late pseudotime, while ES treatment shifted the distribution toward earlier states, partially recovering a Young-like profile. Together, these findings suggest that ES mitigates aging-associated differentiation arrest and reprograms NP cells toward a younger trajectory. Cell-cell communication analysis (Fig. 3D) showed that ES enhanced multiple signaling pathways linked to repair, regeneration, and stemness maintenance. Up-regulated pathways included angiogenesis and proliferation (VEGF, platelet-derived growth factor (PDGF), insulin-like growth factor (IGF), TGF-β), developmental and stemness-related signaling (NOTCH, EPHA, NETRIN, BMP, NCAM), and ECM homeostasis (COLLAGEN, FN1, LAMININ, THBS, SPP1, TENASCIN, PERIOSTIN). Moreover, immune regulatory pathways (APOE, THY1, IGFBP) were enriched in the ES group, consistent with a shift toward a regenerative microenvironment.

Metabolomic profiling provided complementary evidence of metabolic reprogramming. Principal component analysis (PCA) showed clear separation among the 3 groups, with ES samples clustering closer to the Young group (Fig. 3E). Gene Ontology (GO) enrichment analysis revealed that ES down-regulated dysregulated pathways, including oxidative phosphorylation, phosphatidylinositol signaling, inositol phosphate metabolism, neurodegeneration, and ROS-related carcinogenesis—while up-regulating core metabolic processes such as fatty acid metabolism, protein digestion and absorption, tricarboxylic acid (TCA) cycle, glycolysis/gluconeogenesis, and amino acid biosynthesis, along with antioxidant pathways (ubiquinone biosynthesis, pentose phosphate pathway, nicotinate/nicotinamide metabolism) (Fig. 3F). KEGG analysis further showed that ES enhanced angiogenesis, vascular morphogenesis, tissue remodeling, cell adhesion, and migration while reducing elastin catabolism and stress-response pathways, suggesting that ES leads to lower ECM degradation and inflammatory reactivity (Fig. 3G).

Heatmap analysis in NP cells revealed clear clustering patterns across Young, Aging, and ES groups (Fig. 3H). Cell cycle and proliferation genes (*Ccnd1*, *Cdk1*, *Cdk2*, *Mcm* family genes, *Pcna*) were abundant in Young and ES groups but substantially reduced in Aging. By contrast, senescence- and SASP-associated genes (*Il1b*, *Tnf*, *Mmp3*, *Mmp13*, *Tgfb1*, *Cdkn1a*) were increased in Aging but suppressed in ES to levels comparable with Young. Hierarchical clustering confirmed that ES samples clustered more closely with Young than Aging, indicating transcriptional rejuvenation. To capture dynamic metabolic trajectories, Mfuzz analysis identified 4 metabolite clusters with distinct temporal patterns (Fig. 3I). Gene clustering analysis showed that NP-associated genes followed 4 distinct trajectories across Young, Aging, and ES groups, reflecting different modes of regulation under senescence and stimulation (Fig. 3J and Table S1). Genes in cluster 1 that declined with aging were reactivated by ES, indicating the recovery of proliferative and metabolic programs that are typically suppressed in senescent cells. In contrast, cluster 2 genes showed a steady decrease from Young to Aging to ES, suggesting transcriptional modules that were less responsive to rejuvenation. Cluster 3 trajectory included genes that were aberrantly induced during aging but strongly suppressed by ES, highlighting the capacity of stimulation to dampen inflammatory and SASP-related responses. Finally, genes in cluster 4 increased progressively with aging but were partially reduced under ES, indicating mitigation of stress-adaptive or metabolite-accumulation processes.

Overall, these dynamic patterns demonstrate that ES exerts complementary restorative and inhibitory effects, thereby selectively reprogramming NP cells toward a more youthful and regenerative state.

### Microsphere-derived electromechanical cues attenuate cellular senescence in the intervertebral disc

After observing that ES reversed aging-associated molecular features at both transcriptomic and metabolomic levels (Fig. 3), we investigated whether microsphere-mediated ES attenuated senescence-related phenotypes in NP cells (Fig. 4A).

**Fig. 4.**
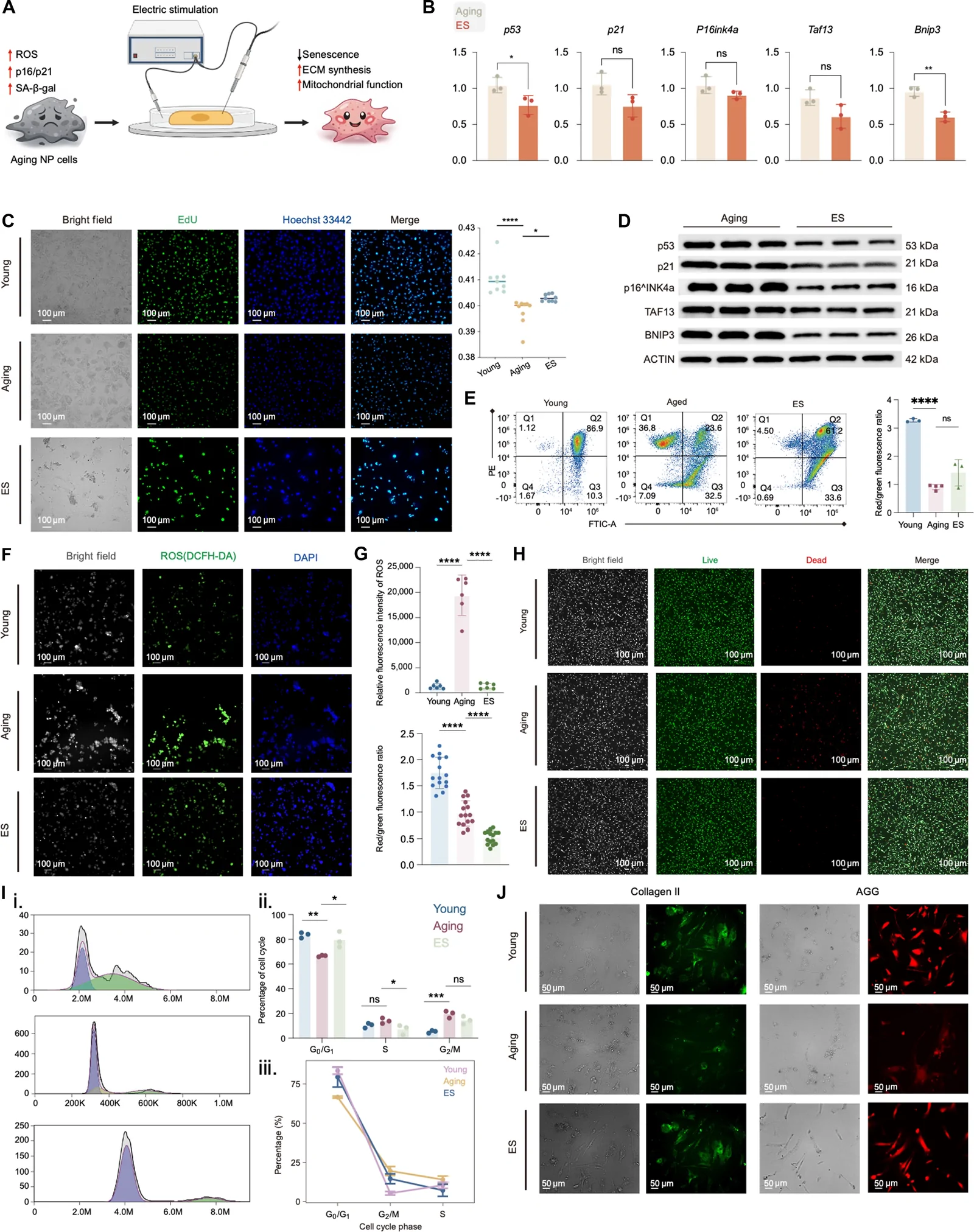
Microsphere-based intervention attenuates senescence in NP cells. (A) Schematic illustration of the experimental design. (B) qPCR analysis of Aging and ES groups. ES significantly down-regulated *p53* and *Bnip3*, whereas *p21*, *p16^INK4a^*, and *Taf13* showed no significant changes (*n* = 3). (C) EdU staining showing cell proliferation activity (*n* = 9). (D) Western blot and quantitative analysis of Aging and ES groups. ES down-regulates p53, p21, p16^INK4a^, TAF13, and mitophagy-related protein (BNIP3) (*n* = 3). (E) JC-1 staining flow cytometry analysis of mitochondrial membrane potential (*n* = 3). (F) ROS staining showing intracellular ROS levels. (G) Top, quantitative histogram of ROS staining (*n* = 6); bottom, quantitative histogram of live/dead cell staining (*n* = 15). (H) Calcein-AM/PI live/dead cell staining. (I) (i) Flow cytometry analysis of cell cycle distribution. (ii) Quantitative analysis of cell cycle phases (*n* = 3), shown as a bar graph depicting the percentage of cells in G_0_/G_1_, S, and G_2_/M phases across Young, Aging, and ES groups. (iii) Dynamic comparison of cell cycle phase distribution. (J) Immunofluorescence staining of ECM proteins COL II and AGG (*n* = 3).

Senescence-associated beta-galactosidase (SA-β-gal) staining demonstrated that the proportion of senescent cells was significantly higher in the Aging group than in the Young group, whereas ES treatment significantly reduced this proportion (Fig. S3E). Quantitative Polymerase chain reaction (qPCR) analysis showed that ES down-regulated *p53* and *Bnip3* expression, whereas *p21*, *p16^INK4a^*, and *Taf13* were not significantly changed (Fig. 4B). Accordingly, 5-ethynyl-2′-deoxyuridine (EdU) incorporation assays demonstrated that ES rescued the proliferative capacity of senescent NP cells to nearly young-cell levels (Fig. 4C). Western blot analysis further verified that ES decreased the protein levels of p53, p21, p16^INK4a^, TAF13, and BNIP3 (Fig. 4D and Fig. S3F). It is noteworthy that while *p21* and *p16*^*INK4a*^ mRNA levels did not show statistically significant changes upon ES treatment (Fig. 4B), their protein levels were markedly down-regulated (Fig. 4D). This mRNA-protein discrepancy suggests that ES may exert its anti-senescence effects, at least in part, through posttranscriptional or posttranslational regulatory mechanisms, rather than solely through transcriptional control. Collectively, these findings support the attenuation of senescence phenotypes.

Mitochondrial dysfunction is a defining feature of cellular senescence. JC-1 staining revealed a marked reduction in mitochondrial membrane potential in senescent NP cells, as indicated by a decreased red/green fluorescence ratio, and this decrease was partially reversed by ES treatment (Fig. 4E). ROS levels assessed by fluorescent staining were significantly elevated in the Aging group but were substantially reduced after ES (Fig. 4F and G, top). Calcein-AM/propidium iodide (PI) live/dead staining further showed that ES enhanced cell viability in senescent NP cells (Fig. 4G, bottom, and H). Together, these results demonstrate that ES attenuates oxidative stress and improves cell survival in senescent NP cells.

Flow cytometric analysis of cell cycle distribution showed that senescent NP cells accumulated in G_0_/G_1_ phase, with corresponding decreases in S and G_2_/M phase cells (Fig. 4I). ES treatment partially relieved this cell cycle arrest, thereby promoting S-phase entry. Immunofluorescence staining further showed that ES restored the expression of ECM proteins collagen II (COL II) and aggrecan (AGG), which were markedly reduced in senescent NP cells (Fig. 4J). Together, these findings suggest that ES not only promotes proliferation but also improves matrix-synthetic capacity, supporting functional recovery of senescent NP cells.

### NP-microsphere crosstalk promotes macrophage activation

To determine whether ES promotes metabolite-mediated crosstalk between NP cells and macrophages, we conducted metabolomic profiling on conditioned medium from 5 experimental groups: Young NP cells (Young), Aging NP cells (Aging), Aging NP cells + ES (Aging + ES), Aging NP cells cocultured with macrophages (Aging + Mφ), and Aging NP cells + ES cocultured with macrophages (Aging + ES + Mφ) (Fig. 5A).

**Fig. 5.**
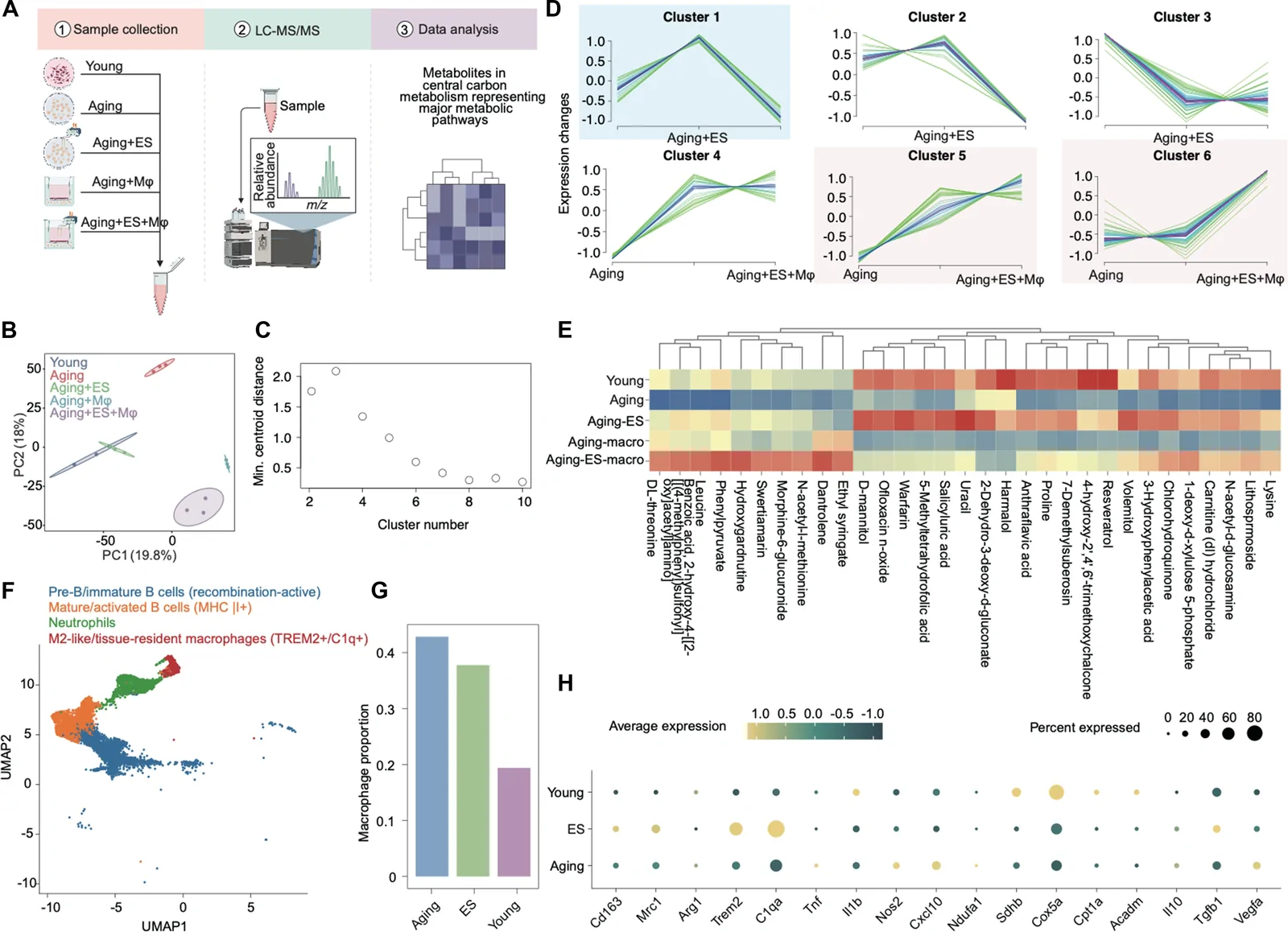
Microsphere intervention in NP cells promotes macrophage activation. (A) Schematic illustration of the experimental design. Samples were collected from each group for metabolomic profiling. (B) PCA of 5 groups: Young, Aging, Aging + ES, Aging + Mφ, and Aging + ES + Mφ. Samples show progressive distribution in PCA space, with ES and coculture gradually remodeling the metabolic profile. (C) Elbow plot of Mfuzz analysis determining optimal the cluster number (*k* = 6). (D) Mfuzz analysis showing dynamic metabolic patterns across Aging, Aging + ES, and Aging + ES + Mφ, identifying 6 distinct metabolite clusters. (E) Heatmap of candidate metabolites showing expression levels across groups. Color indicates relative abundance (red, high, blue, low). (F) Single-cell UMAP of immune cells. (G) Bar plot of M2 macrophage proportions. (H) Dot plot of M2 markers. Treat group macrophages show elevated expression of M2 functional genes (e.g., *Mrc1*, *Arg1*, and *Il10*), whereas those in the Aging group show lower expression, indicating enhanced macrophage function in the Treat group.

We initially compared the metabolomic profiles of Aging and Aging + Mφ groups to evaluate the direct effect of macrophage coculture on senescent NP cells. PCA showed distinct separation between the 2 groups (Fig. S4A), indicating that macrophage interaction substantially reshaped the metabolomic landscape of senescent NP cells. Volcano plot analysis identified differentially accumulated metabolites between these groups (|log₂FC| ≥ 1, false discovery rate (FDR) < 0.05), with red dots indicating metabolites up-regulated upon coculture and blue dots indicating down-regulated metabolites (Fig. S4B). GO enrichment analysis of these differentially abundant metabolites (Fig. S4C and D) demonstrated that metabolites up-regulated in the coculture setting were enriched in anti-inflammatory and tissue-repair pathways, including fatty acid synthesis, phenylalanine/tyrosine/tryptophan metabolism, and coenzyme A biosynthesis. Conversely, metabolites decreased by coculture were enriched in pro-inflammatory and oxidative-stress pathways, such as oxidative phosphorylation, glycolysis/gluconeogenesis, and type II diabetes-related signaling. Together, these findings suggest that macrophage coculture alone can partially offset aging-associated metabolomic alterations in NP cells.

We then examined the global metabolomic relationships among all 5 groups. PCA of the full dataset showed distinct clustering profiles (Fig. 5B). The Young and Aging groups were clearly segregated, consistent with aging-associated metabolic reprogramming. The Aging + ES group clustered more closely with the Young group, indicating that ES partially reversed aging-related metabolomic alterations. Notably, the Aging + ES + Mφ group formed a distinct cluster separate from both Aging + ES and Aging + Mφ groups, indicating that the combination of ES and macrophage coculture induces a unique metabolomic state beyond simple additive effects of either intervention alone.

To capture temporal changes in metabolite levels across experimental conditions, we conducted Mfuzz clustering analysis on the 4 NP-related experimental groups (Young, Aging, Aging + ES, and Aging + ES + Mφ). The elbow plot indicated that 6 clusters best captured the diversity of metabolomic trajectories (Fig. 5C). These 6 clusters displayed distinct dynamic patterns (Fig. 5D and Table S2). Cluster 1 metabolites were reduced in Aging, increased after ES treatment, and were further increased with macrophage coculture, indicating progressive up-regulation. Cluster 2 metabolites decreased in Aging, were partially restored by ES, and stabilized after coculture. Cluster 3 metabolites were increased in Aging but were reduced by ES and were further reduced with coculture, indicating suppression of aging-associated metabolites. Cluster 4 showed rapid induction by ES and maintained high levels upon coculture, suggesting an association with immune activation. Cluster 5 displayed a stepwise increase from Aging to ES to coculture, suggesting coordinated regulation by ES and immune cells. Cluster 6 showed a biphasic pattern, increased in Aging, decreased by ES, and increased again upon coculture. Collectively, these dynamic trajectories indicate that ES and macrophage engagement exert multilayered regulatory effects on the NP cell metabolome. To identify candidate metabolites potentially involved in NP-macrophage crosstalk, we used a stepwise screening strategy (Fig. S4E). This approach identified a panel of 31 candidate metabolites that were enriched in the Aging + ES + Mφ group. Heatmap analysis showed distinct abundance patterns of these metabolites among all 5 groups (Fig. 5E). Notably, this cocktail of metabolites included several molecules with reported roles in macrophage activation and polarization, such as leucine, proline, lysine, carnitine, *N*-acetyl-l-methionine, *N*-acetyl-d-glucosamine, and resveratrol. Collectively, these immunomodulatory metabolites may function as signaling mediators in ES-induced NP-macrophage crosstalk.

To determine whether the identified metabolomic changes were associated with functional alterations in macrophages, we analyzed immune-cell populations by scRNA-seq. Uniform Manifold Approximation and Projection (UMAP) visualization showed distinct clustering of immune-cell populations, including pre-B/immature B cells, mature/activated B cells, neutrophils, and TREM2^+^/C1q^+^ M2-like tissue-resident macrophages (Fig. 5F). Quantification of M2 macrophage proportions across Young, Aging, and ES-treated groups (Fig. 5G) showed that the Aging group contained the highest proportion, the Young group contained the lowest, and the ES-treated group showed an intermediate level, suggesting that ES partially alleviates aging-associated immune-cell accumulation. Dot plot analysis of M2 functional markers (Fig. 5H) showed that the ES group exhibited higher expression of *Mrc1*, *Arg1*, and *Il10* compared with the Aging group, together with higher proportions of positive cells. Additional repair-related factors, including *Tgfb1* and *Vegfa*, were also increased in the ES group. Together, these data support a model in which ES-activated NP cells release immunomodulatory metabolites that promote macrophage activation (Fig. S4F).

To validate whether the metabolite cocktail identified from ES-treated NP cells directly drives functional macrophage activation in a nonlytic manner, we treated macrophages with resveratrol (representing the cocktail) with or without glycine, using lipopolysaccharide (LPS) + nigericin-induced pyroptosis as a reference for lytic GSDMD activation. LPS + nigericin triggered the highest lactate dehydrogenase (LDH) release and lowest cell viability, effects reversed by glycine (*P* = 0.0057). Resveratrol alone induced only moderate LDH release without compromising viability, whereas the resveratrol + glycine combination reduced LDH to near-baseline levels and maintained the highest viability (Fig. S5A and B). Cytokine profiling revealed that LPS + nigericin elicited robust IL-1β, IL-6, and tumor necrosis factor-α (TNF-α) secretion (*P* < 0.001), whereas resveratrol induced a distinct profile, with elevated IL-6 and TNF-α and minimal IL-1β, consistent with functional activation uncoupled from pyroptotic death; glycine further sustained this secretory phenotype while preserving membrane integrity (Fig. S5C). Annexin V/PI staining confirmed that the resveratrol + glycine combination preserved the highest proportion of viable cells (71.0%) with minimal necrosis (3.3%) (Fig. S5D). Accordingly, LPS + nigericin strongly activated caspase-1 and reduced full-length GSDMD, effects attenuated by glycine; resveratrol caused only modest GSDMD cleavage, and the combination preserved full-length GSDMD to levels comparable to controls (Fig. S5E and F). To confirm functional GSDMD pore formation without pyroptotic lysis, we performed additional assays (Control, Res + Gly, LPS+Nig, Dis + Res + Gly, and Dis alone). Res + Gly increased YO-PRO-1 uptake (confirming GSDMD pores) while maintaining low LDH (membrane integrity). Disulfiram reduced both YO-PRO-1 uptake and IL-1β secretion, confirming GSDMD dependence. Caspase-1 was activated in Res + Gly, but IL-1β remained low, well below LPS/Nig, indicating restrained, noncanonical activation (Fig. S5I to L). Thus, NP-derived metabolites induce a non-pyroptotic hyperactivated state defined by caspase-1 activation, GSDMD pore formation, limited IL-1β, and membrane integrity, with glycine acting downstream of pore formation. Collectively, these data indicate that NP-derived metabolites drive macrophages into a nonlytic hyperactivated state, and that glycine sustains this state by osmoprotectively maintaining membrane integrity without interfering with GSDMD cleavage.

To dissect the mechanism by which glycine preserves membrane integrity upon GSDMD pore formation, we next assessed membrane potential dynamics and key protein expression using DiBAC_4_(3) and Western blot. LPS + nigericin induced marked membrane depolarization, whereas glycine significantly attenuated this effect (*P* = 0.0207). This protection was abolished by both a glycine receptor antagonist (*P* = 0.0189) and BAPTA-AM (*P* = 0.0112) (Fig. S5G). Consistently, dysferlin, a calcium-dependent membrane repair protein, was down-regulated in the model group, restored by glycine, and reduced again upon membrane repair blockade, whereas GlyRα1 expression did not differ significantly among groups (Fig. S5H). These results demonstrate that the vita-field preserves membrane integrity through GlyR-coupled, calcium-dependent repair pathways, providing a mechanistic basis for glycine-mediated protection in the context of nonlytic GSDMD activation.

### Macrophage activation induces feedback release of morroniside-like metabolite

To determine whether activated macrophages release feedback metabolites that regulate NP cell function, we constructed an NP-macrophage coculture system and conducted integrated transcriptomic and metabolomic profiling (Fig. 6A).

**Fig. 6.**
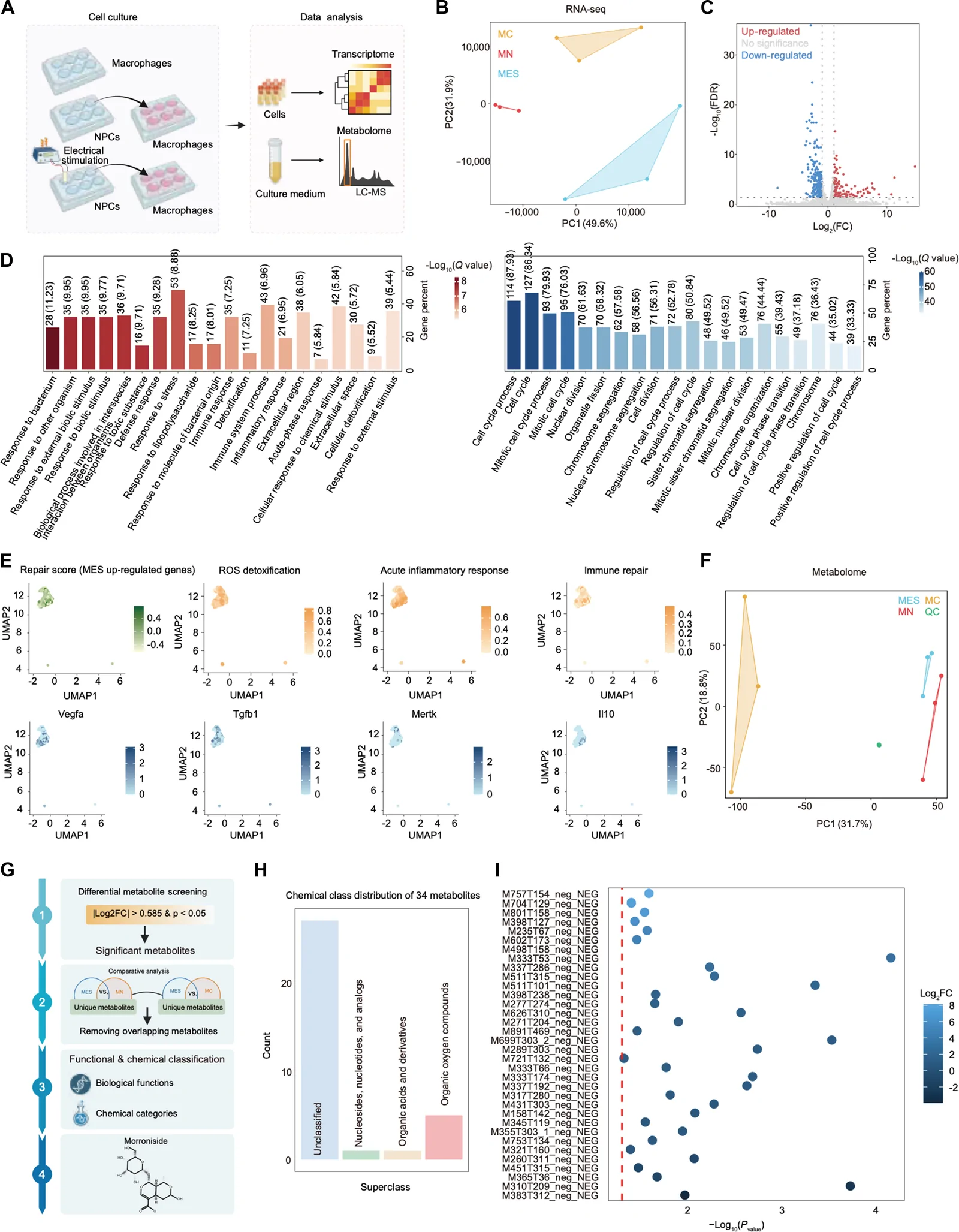
Crosstalk between NP-derived metabolites and macrophages triggers secretion of a morroniside-like feedback metabolite. (A) Coculture system combined with transcriptomic and metabolomic profiling to identify feedback metabolites secreted by macrophages. (B) Transcriptomic PCA. MC (macrophage control), MN (macrophages treated with Aging NP cell-conditioned medium), and MES (macrophages treated with Aging NP cell + ES-conditioned medium) show distinct separation in PCA space. (C) Volcano plot of differentially expressed genes between MN and MES groups. Red dots represent up-regulated genes in MES, blue dots represent down-regulated genes ($\left|\log_{2}\mathrm{FC}\right|\geq 1$, FDR < 0.05). (D) GO enrichment bar plot. Left (red) shows pathways enriched by up-regulated genes in MES; right (blue) shows pathways enriched by down-regulated genes. (E) Single-cell analysis. MES macrophages show an elevated repair score (green), activation of ROS detoxification, acute inflammatory response, and immune repair modules (orange), and up-regulation of repair-related genes *Vegfa*, *Tgfb1*, *Mertk*, and *Il10* (blue). (F) Metabolomic PCA. MC, MN, and MES show gradient distribution in metabolic profiles, consistent with transcriptomic trends. (G) Flowchart of the metabolite screening strategy. (H) Chemical class distribution of 34 candidate metabolites. (I) Manhattan plot of 34 candidate metabolites.

We first determined whether ES-treated NP cells induced transcriptional changes in macrophages. PCA of transcriptomic data showed clear segregation among 3 groups: untreated control macrophages (MC), macrophages treated with conditioned medium from Aging NP cells (MN), and macrophages treated with conditioned medium from Aging + ES NP cells (MES) (Fig. 6B). The MES group clustered separately from both MC and MN, indicating that ES-treated NP cells exert a distinct transcriptional effect on macrophages. Volcano plot analysis comparing MN and MES groups identified a large number of differentially expressed genes in MES (|log₂FC| ≥ 1, FDR < 0.05), with red dots indicating up-regulated genes and blue dots indicating down-regulated genes (Fig. 6C). GO enrichment analysis further showed that genes up-regulated in MES were enriched in immune response, inflammatory response, and external stimulus response pathways, whereas down-regulated genes were associated with cell cycle-related processes, including mitosis and chromosome segregation (Fig. 6D). These findings indicate that ES-treated NP cells shift macrophages toward an immune-activated state while reducing their proliferative activity.

Single-cell transcriptomic analysis further defined the functional phenotype of MES macrophages (Fig. 6E). UMAP visualization showed that MES macrophages exhibited elevated repair scores (green) and activated functional modules related to ROS detoxification, acute inflammatory response, and immune repair (orange). Consistently, repair-associated genes including *Vegfa*, *Tgfb1*, *Mertk*, and *Il10* were markedly up-regulated in MES macrophages (blue). Together, these data indicate that ES-treated NP cells program macrophages toward an anti-inflammatory tissue-repair phenotype.

We next examined whether activated macrophages displayed corresponding metabolomic changes. PCA of metabolomic data showed a graded distribution among MC, MN, and MES groups (Fig. 6F), paralleling the transcriptional trajectories and suggesting coordinated metabolomic remodeling. GO enrichment analysis of metabolic pathways further showed that MES macrophages up-regulated pathways associated with amino acid and lipid metabolism while down-regulating proliferation-associated pathways (Fig. S4G). To systematically define candidate feedback metabolites, we used a stepwise screening strategy (Fig. 6G). Chemical classification of the 34 candidate metabolites (Table S1) showed diverse structural classes, including unclassified compounds, organooxygen compounds, nucleosides/nucleotides, and organic acids (Fig. 6H). Manhattan plot analysis of these candidates showed varying statistical significance (Fig. 6I and Table S3). On the basis of its enrichment pattern in the MES group across multiple comparisons and its reported bioactivities related to tissue protection and anti-inflammatory activity, a morroniside-like metabolite (putatively annotated by high-resolution MS (HRMS) and MS/MS; MSI level 2) was selected for subsequent functional validation.

To validate the metabolomic identification and characterize the cellular source and release mechanism of the morroniside-like metabolite, we performed targeted liquid chromatography (LC)-tandem mass spectrometry (MS/MS) multiple reaction monitoring (MRM) absolute quantification, serum-free culture, GSDMD inhibition, and HRMS analyses (Fig. S4H to N). Targeted MRM quantification revealed a stepwise increase in the morroniside-like metabolite concentration across groups (Fig. S4H). The morroniside-like metabolite level in the Aging + ES + Mφ supernatant (561.82 ± 3.89 ng/ml) was significantly higher than that in the Control (52.59 ± 0.50 ng/ml) and Aging + Mφ (129.28 ± 20.24 ng/ml) groups (*P* < 0.001). Under serum-free conditions, the morroniside-like metabolite concentration (96.20 ± 0.90 ng/ml) remained readily detectable, albeit lower than in serum-containing cultures (561.82 ± 3.89 ng/ml, *P* < 0.001) (Fig. S4I), indicating endogenous synthesis rather than serum-derived contamination. GSDMD inhibition with disulfiram reduced morroniside-like metabolite release from 561.82 ± 3.89 ng/ml to 340.16 ± 3.51 ng/ml (~40% decrease, *P* < 0.001) (Fig. S4J), demonstrating that GSDMD pore formation is a major secretory route. HRMS analysis confirmed that the candidate metabolite shared the same molecular formula (C_17_H_25_O_11_) as morroniside, as evidenced by the [M + Na]^+^ ion at mass/charge ratio (*m*/*z*) 428.13 in both samples (mass errors: 5.98 parts per million (ppm) for [M + H]^+^ and 12.17 ppm for [M + Na]^+^) (Fig. S4K). However, the 2 compounds exhibited distinct retention times (0.591 min for morroniside versus 0.137 min for the candidate) (Fig. S4L and M), excluding the possibility that they are identical. The candidate also showed a strong signal in negative ion mode (Fig. S4N), further confirming its molecular weight. Taken together, these data suggest that the candidate metabolite is a structural analog of morroniside that is produced and released by activated macrophages in a GSDMD-dependent manner. Given that the endogenous metabolite is a structural analog of morroniside sharing the same molecular formula and core iridoid glycoside scaffold, and that its trace abundance precludes isolation for direct functional testing, we used commercially available authentic morroniside as a pharmacological surrogate to represent this class of compounds in functional assays.

### Macrophage-derived morroniside-like metabolite attenuates senescence in NP cells

To determine whether authentic morroniside exerts anti-senescent effects in NP cells and whether this effect depends on YAP/TAZ signaling, we established 4 treatment groups: Young NP cells (C), senescent NP cells (S), senescent NP cells treated with authentic morroniside (S-M), and senescent NP cells treated with authentic morroniside together with a YAP/TAZ inhibitor (S-MY) (Fig. S4O). As shown in the working mechanistic model (Fig. 7A), the morroniside-like metabolite released by activated macrophages acts on senescent NP cells to regulate the phosphorylation status of Hippo pathway kinases MST1/2 and LATS1/2, thereby modulating downstream YAP/TAZ activity.

**Fig. 7.**
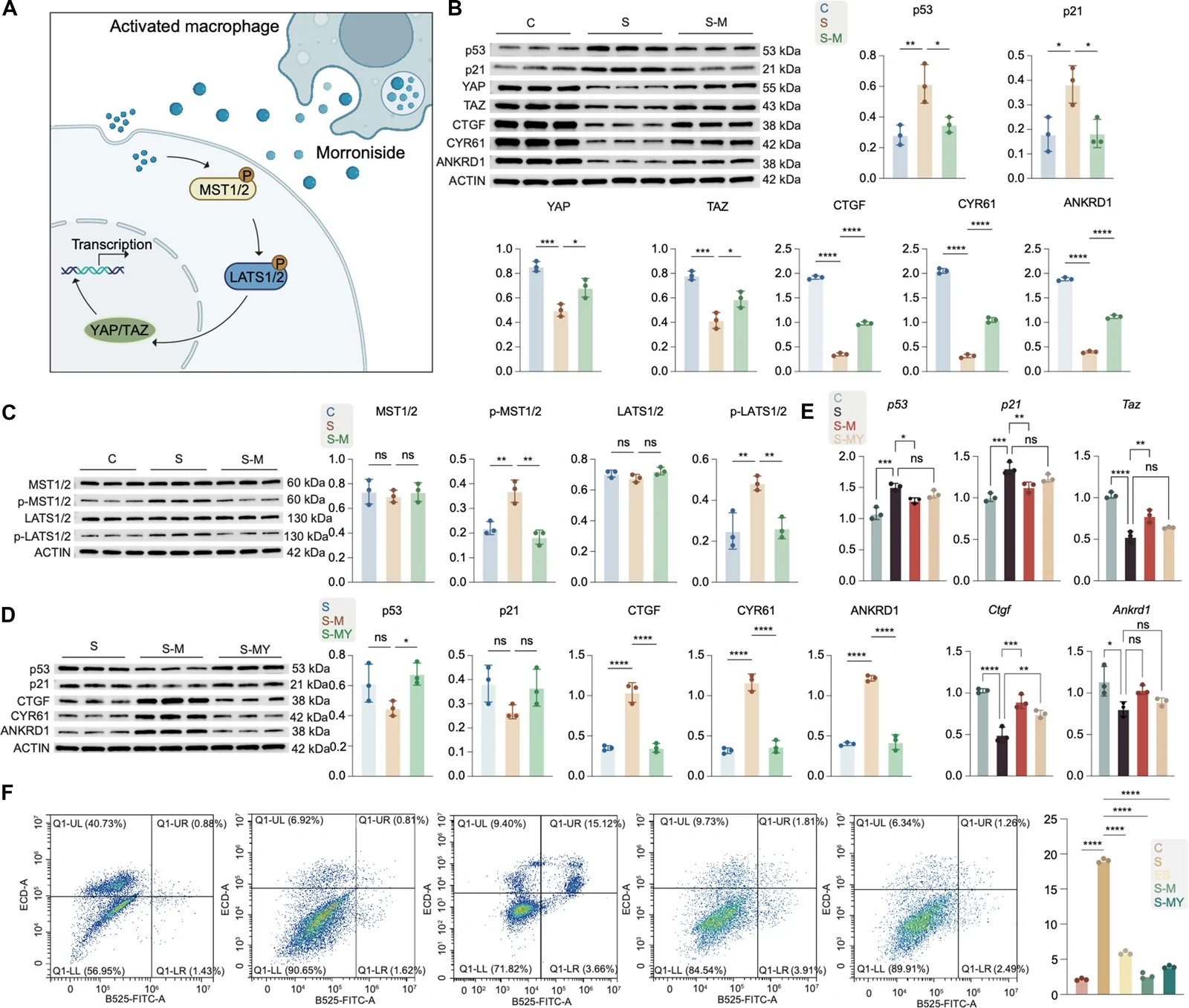
A metabolite-macrophage-morroniside feedback loop regulates NP cell senescence via the Hippo pathway. (A) Morroniside-like metabolites released from activated macrophages inhibit MST1/2 and LATS1/2 phosphorylation, activate YAP/TAZ signaling, and thereby suppress NP cell senescence while promoting ECM synthesis. (B) Western blot of C (Young NP cells), S (Aging NP cells), and S-M (Aging NP cells + authentic morroniside) groups. Authentic morroniside treatment down-regulates p53 and p21, and up-regulates the Hippo pathway effectors TAZ and YAP and their target genes CTGF, CYR61, and ANKRD1 (*n* = 3). (C) Western blot of Hippo pathway kinases in C, S, and S-M groups. Authentic morroniside treatment inhibits phosphorylation of MST1/2 and LATS1/2, indicating activation of YAP/TAZ through suppression of the Hippo pathway (*n* = 3). (D) Western blot of S, S-M, and S-MY (Aging NP cells + authentic morroniside + YAP/TAZ inhibitor) groups. YAP/TAZ inhibitor reverses authentic morroniside-induced down-regulation of p53 and p21 and up-regulation of CTGF, CYR61, and ANKRD1, indicating that morroniside functions through YAP/TAZ signaling (*n* = 3). (E) qPCR analysis of C, S, S-M, and S-MY groups. YAP/TAZ inhibitor reverses morroniside-induced down-regulation of *p53 and p21* and up-regulation of *Taz*, *Ctgf*, and *Ankrd1*, consistent with protein-level results (*n* = 3). (F) Flow cytometry showing differences in cell cycle and apoptosis distribution (C, S, ES, S-M, and S-MY groups) (*n* = 3).

Western blot analysis of C, S, and S-M groups (Fig. 7B) showed that morroniside markedly down-regulated the senescence-associated proteins p53 and p21. Concurrently, authentic morroniside increased the Hippo pathway effectors YAP and TAZ, as well as their downstream target proteins CTGF, CYR61, and ANKRD1. These results suggest that morroniside suppresses senescence signaling and activates Hippo pathway effectors.

To define the upstream mechanism, we assessed Hippo pathway kinase activity in C, S, and S-M groups (Fig. 7C). Total MST1/2 and LATS1/2 protein levels were unchanged across groups; however, phosphorylation levels were markedly increased in the S group and significantly reduced after authentic morroniside treatment. These findings indicate that morroniside promotes YAP/TAZ activation by reducing MST1/2 and LATS1/2 phosphorylation.

We next tested pathway specificity using a YAP/TAZ inhibitor. Western blot analysis of S, S-M, and S-MY groups (Fig. 7D) showed that authentic morroniside-induced up-regulation of CTGF, CYR61, and ANKRD1 was markedly reversed by treatment with a YAP/TAZ inhibitor. Similarly, the morroniside-like metabolite-mediated changes in p53 and p21 were partly reversed. qPCR analysis supported these observations: authentic morroniside down-regulated *p53* and *p21* and up-regulated *Taz, Ctgf*, and *Ankrd1* in the S-M group, and these effects were attenuated in the S-MY group (Fig. 7E). Collectively, these results indicate that morroniside exerts its anti-senescence effects in part through YAP/TAZ-dependent signaling.

Finally, we evaluated the functional effect of morroniside on NP cell survival. Flow cytometric analysis of C, S, ES, S-M, and S-MY groups (Fig. 7F) showed that the S group had a marked increase in apoptotic cells. Both ES and S-M markedly reduced apoptosis. Notably, treatment with the YAP/TAZ inhibitor partly reversed the anti-apoptotic effect of authentic morroniside in the S-MY group. These results indicate that authentic morroniside inhibits apoptosis in senescent NP cells through YAP/TAZ signaling.

### In vivo validation of Dual-stimulus Microspheres in Aged Disc Models

To assess the therapeutic efficacy of dual-field microspheres in vivo, we generated a rat model of IVDD and monitored disc height, magnetic resonance imaging (MRI) signal intensity, and histological alterations for 8 weeks in 4 groups: Aging, Lesion, ES, and Dual-regulation (microspheres + ES).

We first evaluated structural maintenance by x-ray and MRI. X-ray analysis showed that the Dual-regulation group preserved stable disc height throughout the observation period, at a level comparable to the Aging group, whereas the Lesion and ES groups showed progressive decreases (Fig. 8A). MRI signal intensity, a marker of hydration and tissue integrity, remained low and relatively stable in the Lesion group, consistent with ongoing degeneration, but increased substantially in the Dual-regulation group, with a peak at 8 weeks (Fig. 8B). Together, these imaging results indicate that dual-regulation effectively preserves disc structure and hydration, whereas ES alone provides only partial protection.

**Fig. 8.**
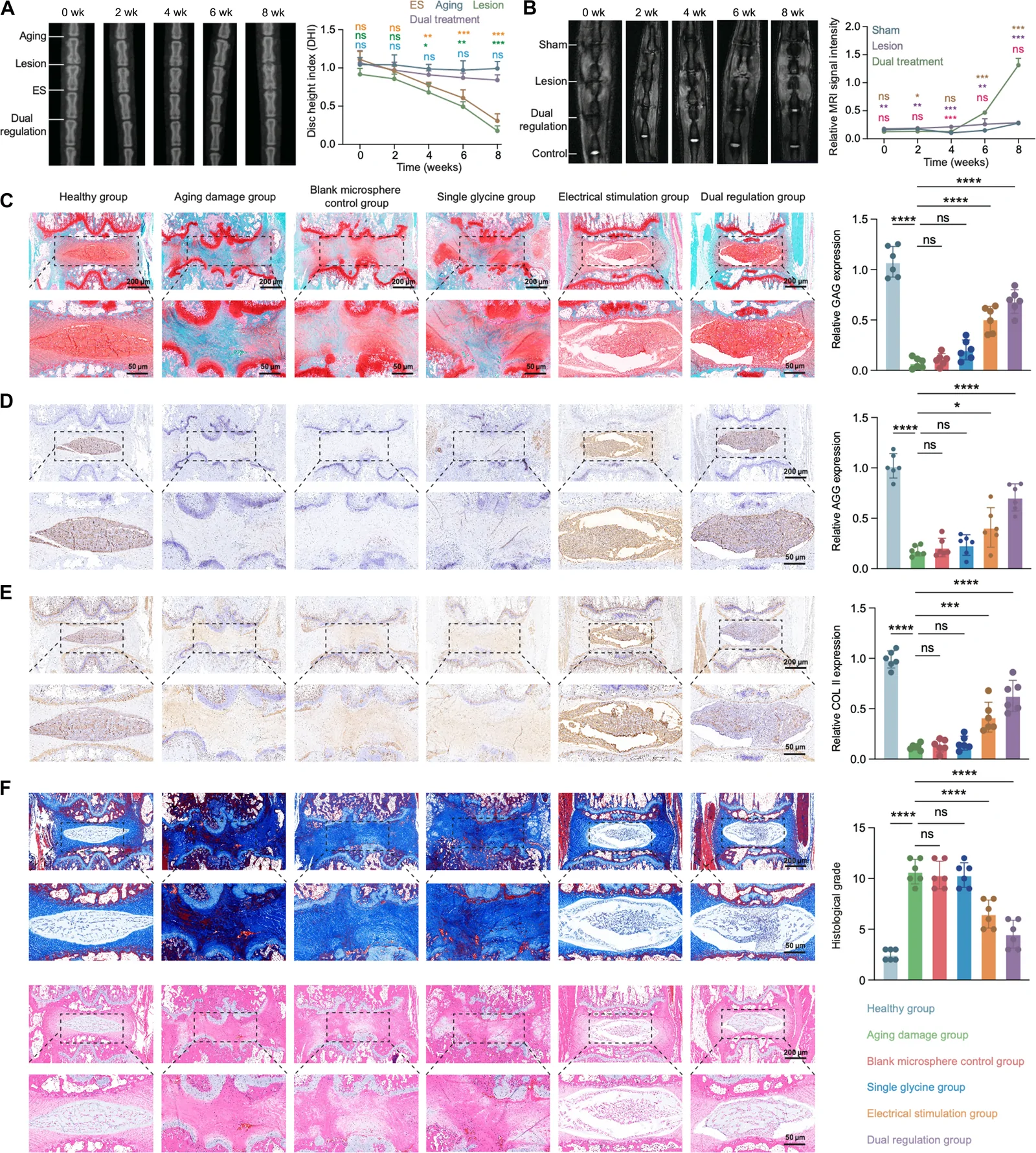
Dual-regulation microspheres preserve disc structure and matrix integrity in vivo. (A) Representative x-ray images of rat intervertebral discs at 0 to 8 weeks under 4 conditions: Aging (11-month-old untreated rats), Lesion (11-month-old + needle puncture), ES (Lesion + ES), and Dual-regulation (Lesion + microspheres + ES). Quantification of DHI shows stable disc height in the Dual-regulation group, whereas the Lesion and ES groups exhibit progressive declines. Data are presented as mean ± SD (*n* = 3 per group per time point). Statistical significance was determined by 2-way repeated-measures ANOVA with Tukey’s post hoc test. Orange symbols indicate Aging versus Lesion; green symbols indicate Aging versus ES; blue symbols indicate Aging versus Dual-regulation. (B) Representative MRI scans of discs at 0 to 8 weeks across 4 groups: Control (8-week-old untreated rats, set as the normalization baseline with a value of 1), Sham (11-month-old + needle puncture without rotation), Lesion (11-month-old + needle puncture with rotation), and Dual-regulation (Lesion + microspheres + ES). Data are mean ± SEM (*n* = 3 per group). Statistical significance was determined by 2-way repeated-measures ANOVA with Tukey’s post hoc test. Light brown symbols indicate Dual-regulation versus Sham; purple symbols indicate Dual-regulation versus Lesion; pink symbols indicate Lesion versus Sham. (C) Safranin O-Fast Green staining of disc sections showing GAG distribution. Quantitative analysis of relative GAG content reveals severe depletion in the Aging damage group (11-month-old + needle puncture), partial restoration by ES, and substantial recovery by dual-regulation (*n* = 6). Healthy control (8-week-old untreated rats) is shown for reference. (D) AGG staining of disc sections. Quantification of relative AGG expression confirms enhanced matrix deposition in the Dual-regulation group compared with ES alone (*n* = 6). (E) COL II staining of disc sections. Quantitative analysis of relative COL II expression shows significant restoration in the Dual-regulation group, approaching levels of healthy discs (*n* = 6). (F) Masson’s trichrome (up) and H&E staining (down) of disc sections. Combined histological scoring demonstrates severe structural disorganization in the Aging damage group, partial improvement with ES, and marked structural restoration in the Dual-regulation group (*n* = 6).

To investigate the molecular basis of these structural benefits, we assessed matrix composition by histology. Safranin O-Fast Green staining revealed that glycosaminoglycan (GAG) content was markedly depleted in the Aging damage group, blank microsphere control group, and Glycine only group, with no significant differences among these groups. ES treatment partially restored GAG content, and dual-regulation substantially restored it to levels close to those of healthy discs (Fig. 8C). Similarly, AGG and COL II staining indicated that dual-regulation markedly enhanced matrix deposition relative to ES alone (Fig. 8D and E). Quantitative analyses verified that GAG, AGG, and COL II levels were highest in the healthy group, followed by the Dual-regulation, ES, and Aging damage groups. The Microsphere only and Glycine only groups showed levels comparable to the Aging damage group, with no statistically significant differences among them.

Consistent with these biochemical findings, histological evaluation of tissue architecture showed marked structural restoration after dual-regulation. Masson’s trichrome and hematoxylin and eosin (H&E) staining demonstrated that the Aging damage group, Microsphere only, and Glycine only groups displayed severe structural disorganization, collagen-fiber disruption, and matrix loss, whereas ES treatment produced only partial improvement (Fig. 8F). Notably, the Dual-regulation group showed well-preserved tissue architecture, with organized collagen fibers and matrix distribution approaching that of healthy discs. Quantitative histological scoring supported these observations, with the Dual-regulation group showing markedly lower scores than the Aging damage group.

Collectively, these in vivo data indicate that dual-regulation, combining microsphere-mediated delivery with ES, effectively preserves disc height, restores matrix composition, and maintains tissue architecture, outperforming ES alone in mitigating age-related disc degeneration. The lack of efficacy in the Microsphere only and Glycine only groups further confirms that the therapeutic benefit requires the coordinated action of both the electro-field and the vita-field.

## Discussion

IVDD is a major cause of chronic low back pain, yet effective therapeutic interventions remain limited [23]. The distinct avascular, hypoxic, and nutrient-restricted microenvironment of the disc renders NP cells highly vulnerable to aging-associated stress. Meanwhile, the immune-privileged status of the NP region is disrupted during degeneration, allowing peripheral immune cells—especially macrophages—to infiltrate the disc and form a maladaptive feedback loop with senescent NP cells [24,25].

Recent advances in piezoelectric biomaterials have demonstrated the feasibility of ES for disc regeneration. Gao et al. [26] developed a sono-piezoelectric hydrogel that activates Ca^2+^/CaMKII/Parkin-mediated mitophagy to restore mitochondrial function in NP cells. Pan et al. [27] engineered an ultrasound-responsive cerium dioxide, poly(L-lactic acid), and sodium alginate (CPS) piezoelectric hydrogel that drives macrophage M2 polarization via nuclear factor κB (NF-κB) inhibition for annulus fibrosus repair. Liu et al. [28] constructed a GelMA-FF piezoelectric scaffold that modulates phosphatidylinositol 3-kinase (PI3K)-AKT signaling to shift disc metabolism from inflammatory catabolism to anabolic regeneration. Despite these advances, these platforms primarily employ unidirectional stimulation targeting NP cells or immune cells in isolation, without establishing a sustained bidirectional crosstalk between cell types. Similarly, microneedle-based delivery systems have been explored for disc repair. Park et al. [29] comprehensively reviewed polymer microneedles for localized drug delivery in musculoskeletal tissues. Zhou et al. [30] developed circular microneedles for the delivery of CAR-M-like engineered macrophages to enhance efferocytosis capacity, while Zhang et al. [31] fabricated a self-powered triboelectric-responsive microneedle array for the controlled release of optogenetically engineered extracellular vesicles. However, these systems function predominantly as passive delivery vehicles for exogenous therapeutic agents and lack the capacity to actively modulate endogenous intercellular metabolic communication.

In this study, we developed a bioelectronic-immunometabolic strategy that integrates localized ES with metabolite feedback regulation [18]. By using injectable PLLA-HAMA microspheres, we delivered programmable ES directly to the degenerative disc niche, thereby reprogramming the transcriptional, metabolic, and functional phenotypes of senescent NP cells. Moreover, ES-activated NP cells induce macrophages to enter a metabolically activated, tissue-repairing state. In contrast to traditional interventions that target only NP cells, our findings reveal a previously unrecognized “ES-NP-macrophage-metabolite” feedback axis [32], offering a system-level paradigm for reversing disc degeneration. Recent advances in material-enabled biomedical therapies, such as adaptive nanovesicles for oral insulin delivery [33] and engineered exosomes for diffuse intrinsic pontine glioma (DIPG) therapy [34], further highlight how innovative material platforms can overcome biological barriers and achieve targeted therapeutic effects. Although distinct from piezoelectric biomaterials for intervertebral disc regeneration, these studies collectively underscore the importance of material-driven strategies in next-generation regenerative medicine. It is well recognized that percutaneous disc puncture carries inherent risks, including annulus fibrosus disruption and nerve root irritation. However, the threaded-groove design of our microneedle (Fig. 2J), together with the thermosensitive hydrogel carrier, physically anchors the functionalized microspheres and prevents premature loss during tissue penetration. This design substantially minimizes the risk of microsphere leakage into the spinal canal or adjacent nerve roots upon needle withdrawal, representing a significant advantage over conventional discography or percutaneous discectomy needles (typically 18-22 gauge). Encouragingly, in vivo safety monitoring over an 8-week observation period revealed no behavioral abnormalities, local infections, nerve root adhesions, or mortality across all treatment groups, supporting the short-term tolerability of this system. Although short-term in vivo monitoring (8 weeks) revealed no adverse events, PLLA degradation typically spans 12-24 months, producing lactic acid that may exacerbate acidosis in the hypoxic disc niche. Moreover, long-term retention of empty PLLA shells could potentially trigger foreign body reactions or mechanical irritation. These risks were not captured within the present study and warrant systematic long-term evaluation. Future work will focus on monitoring PLLA degradation kinetics and exploring copolymer or surface-modified designs to mitigate potential adverse effects.

Previous studies have demonstrated that ES exerts multilayered regulatory effects in tissue regeneration—including modulation of cell proliferation, migration, and differentiation as well as remodeling of mitochondrial dynamics and redox homeostasis [35,36]. However, how ES remodels the molecular programs remains insufficiently understood. By integrating single-cell transcriptomics and metabolomics, we found that ES not only suppresses canonical senescence markers but also promotes up-regulation of ECM-related genes (such as COL2A1 and SOX9) and mitochondrial metabolism-related genes (such as MT-ND4 and COX5B), together with activation of oxidative phosphorylation and ECM-organization pathways. The spatially targeted delivery of our system is supported by the threaded-groove microneedle design (Fig. 2J), which physically anchors microspheres within the target disc region and prevents premature loss during tissue penetration, while the temporally sustained release of glycine over 21 d is confirmed by the cumulative release profile (Fig. S1G). However, we acknowledge that direct quantitative evidence of the electric field distribution and glycine concentration gradient within the disc space was not obtained in the present study. Future studies employing microelectrode array mapping or fluorescence-labeled glycine tracking will be required to quantitatively define the spatial extent and gradient of the dual-field within the disc microenvironment.

To determine whether these beneficial effects of ES are coupled with a coordinated protective mechanism for macrophages, we further dissected the functional roles of the electro-field and the vita-field within this dual-field system. The electro-field acts as the initiating signal by rejuvenating senescent NP cells, ameliorating their aging phenotype and driving the secretion of a metabolite cocktail. This cocktail subsequently activates infiltrating macrophages and induces GSDMD pore formation, a process that carries the inherent risk of membrane damage and pyroptotic death. At this critical stage, the vita-field intervenes by protecting macrophage membrane integrity during pore formation, thereby preventing pyroptosis. Through this division of labor, macrophages remain in a hyperactivated yet viable state, continuously releasing reparative factors such as morroniside-like metabolite. This metabolite then feeds back to NP cells, further consolidating the anti-senescence effect. Thus, the electro-field initiates the cascade, while the vita-field ensures its sustainability, together forming a temporally and functionally coupled therapeutic axis (Fig. S6). We acknowledge the limitations of the pharmacological inhibitors used to probe the membrane protection pathways downstream of GSDMD pore formation. Strychnine, a glycine receptor (GlyR) antagonist, was employed to assess whether glycine’s protective effect is mediated through GlyR. While strychnine is widely used for this purpose, potential off-target effects cannot be fully excluded. We therefore interpret glycine’s protection as being at least partially mediated through GlyR while acknowledging possible off-target actions of strychnine. Similarly, BAPTA-AM was used to probe calcium-dependent membrane repair, but its limitations as a pharmacological chelator are recognized. Our data suggest that GlyR and calcium signaling contribute to glycine’s protective effect, although the specificity of these pharmacological tools is not absolute. Future studies employing genetic approaches (e.g., GlyR knockdown or calcium channel knockout) will be required to confirm these mechanisms.

Mechanistically, we further found that ES mediated this reprogramming through the Hippo-YAP/TAZ signaling pathway. ES reduces the phosphorylation of the core Hippo kinases MST1/2 and LATS1/2, thereby allowing nuclear translocation of YAP/TAZ and activation of their downstream transcriptional programs. This results in the up-regulation of pro-repair genes such as CTGF, CYR61, and ANKRD1 while simultaneously down-regulating senescence-associated genes including p53 and p21. This finding extends the current understanding of the anti-senescence mechanisms of ES [37]. While previous work has demonstrated that ultrasound-responsive piezoelectric hydrogels can attenuate NP cell senescence through activation of the AMP-activated protein kinase (AMPK)-FOXO1a pathway to promote mitophagy, our study identifies an additional mechanistic axis—the Hippo-YAP/TAZ pathway—through which ES reprograms senescent cells. These results indicate that ES may achieve broad functional recovery of aged cells through the coordinated activation of multiple signaling pathways, providing new insight into how bioelectrical cues govern cell fate decisions. The differential regulation of p21 and p16INK4a at the mRNA and protein levels underscores the complexity of ES-mediated senescence mitigation, which likely involves both transcriptional reprogramming (as evidenced by changes in p53 and Bnip3) and posttranscriptional regulation (as inferred from p21 and p16INK4a protein down-regulation without corresponding mRNA changes). Future studies employing polysome profiling or ubiquitination assays will be required to further elucidate the precise mechanisms underlying this posttranscriptional regulation. While CTGF is a canonical YAP/TAZ target, its up-regulation in senescence reflects a stress/fibrotic signature rather than a regenerative signal. In the degenerating disc, CTGF contributes to NP fibrosis and correlates with decreased AGG and COL II. Thus, authentic morroniside-induced CTGF up-regulation represents restoration of homeostasis, a conclusion supported by concurrent p53/p21 reduction and ECM recovery.

It is important to clarify that while our data demonstrate that the morroniside-like metabolite is endogenously produced by macrophages, as supported by its detection in serum-free conditioned medium and its absence in cell-free controls, our experiments do not formally establish the biosynthetic pathway (e.g., de novo synthesis from simple precursors versus conversion from intracellular pools of related iridoid glycoside precursors). We therefore refer to the metabolite as “endogenously produced” or “cell-derived”. This distinction does not affect the central conclusion that the metabolite is produced by macrophages in an activation- and GSDMD-dependent manner.

A second major finding of this study is that activated macrophages do not simply execute downstream effector functions; instead, they actively release morroniside-like metabolite (a structural analog), which feeds back to NP cells and further stabilizes their “repaired state”. This finding shifts the regulatory framework from a unidirectional cascade—ES → NP cells → macrophages—into a bidirectional, closed-loop, self-sustaining metabolite–dialogue network. Mechanistically, authentic morroniside (used as a functional surrogate) activated the Hippo-YAP/TAZ pathway in NP cells, thereby promoting anabolic ECM synthesis while suppressing apoptosis. Loss-of-function experiments further supported the necessity of this feedback loop: blocking morroniside-like metabolite-mediated signaling in the full 3-component coculture system markedly reduced expression of repair-associated markers and ECM synthesis in NP cells, without altering macrophage activation. These findings support the role of the morroniside-like metabolite as a key mediator of macrophage-to-NP feedback signaling.

From a broader biological standpoint, such metabolite-mediated intercellular feedback loops may constitute a conserved regulatory module in tissue repair. In the study by Wang and colleagues [38], GSDMD-dependent metabolic communication was defined as a unidirectional macrophage-to-stem-cell signal mediated by a single oxylipin metabolite (11,12-EET) in muscle regeneration. In contrast, our work identifies a distinct and more complex form of communication: NP cells initiate macrophage hyperactivation through a metabolite cocktail enriched with immunomodulatory molecules (including leucine, proline, lysine, carnitine, and resveratrol), whereas hyperactivated macrophages subsequently strengthen NP repair programs through morroniside-like metabolite. The emergence of this bidirectional metabolite dialogue—in which a synergistic mixture of NP-derived metabolites activates macrophages, which in turn feeds back a single small-molecule signal to NP cells—endows the system with self-sustaining and amplifying properties, which may explain why ES-based therapies sustain prolonged regenerative effects even after cessation of stimulation. We acknowledge that resveratrol alone may not fully recapitulate the activity of the complete 7-metabolite cocktail. Resveratrol was selected as a representative based on its enrichment in our metabolomic screen and its well-documented immunomodulatory bioactivity. Future studies employing the complete metabolite cocktail will be valuable to further validate the findings observed with resveratrol.

Collectively, this study reveals an ES-initiated, metabolite-mediated bidirectional communication network between NP cells and macrophages. Through ES-induced NP-cell reprogramming, a macrophage-activating metabolite cocktail derived from NP cells, and morroniside-mediated feedback reinforcement, this network achieves a multicellular therapeutic effect on disc degeneration. By integrating biophysical stimulation (ES) with immunometabolic regulation, we propose an “ES-immunometabolic axis” as a new paradigm for intervertebral disc regeneration and provide preclinical evidence for metabolite-based strategies in tissue repair. More broadly, this framework may be extended to other degenerative diseases defined by cellular senescence and chronic inflammation, thereby opening new avenues for the development of next-generation “intelligent” regenerative therapies.

## Conclusion

This study presents a Chinese microneedle-microsphere system that creates a spatially precise dual-field microenvironment within degenerated intervertebral discs. The electro-field attenuates NP cell senescence and promotes secretion of a defined cocktail of metabolites that shifts macrophages toward a non-pyroptotic hyperactivated state via GSDMD pore formation, whereas the vita-field preserves macrophage viability to support sustained secretion of reparative factors. Importantly, we identify a morroniside-like metabolite as a macrophage-derived feedback metabolite that acts on NP cells through the Hippo-YAP/TAZ pathway, thereby forming a bidirectional, self-sustaining intercellular communication circuit. Collectively, this work proposes an “electro-immunometabolic axis” as a new therapeutic paradigm for disc regeneration, providing a platform for deep-tissue repair and next-generation regenerative therapies.

## Materials and Methods

### Materials

HAMA (150 kDa) was purchased from Shanghai Yuju Tech Co. Ltd. (Shanghai, China). PLLA was supplied by Dr. F.L.’s laboratory. NP cells (C57BL/6, primary) and mouse macrophages (RAW264.7 cell line) were purchased from Hangzhou Junyue Biotech Co. Ltd. (Zhejiang, China). RNA-Quick Purification kit was obtained from Shanghai Esunbio Biotechnology Co. Ltd. (Shanghai, China). RNA Isolator Total RNA Extraction Reagent was acquired from Vazyme Biotech Co. Ltd. (Jiangsu, China). Cell Cycle and Apoptosis Kit, Calcein/PI Cell Viability/Cytotoxicity Assay kit, Senescence β-Galactosidase Staining kit, Reactive Oxygen Species kit, and 2′,7′-dichlorodihydrofluorescein diacetate (DCFH-DA) probe and JC-1 mitochondrial membrane potential detection kit were provided by Beyotime Biotechnology (Shanghai, China). Live and Dead Cell Staining kit was obtained from Abbkine Scientific Co. Ltd. (Wuhan, China). Dulbecco’s modified Eagle’s medium (DMEM) and fetal bovine serum (FBS) were purchased from Gibco (China). Morroniside (HY-N0532) and YAP/TAZ inhibitor (HY-111429) were purchased from MedChemExpress (China). Male Sprague-Dawley rats (8 weeks and 11 months) were obtained from Center for Laboratory Animal Science, Hangzhou Medical College (Zhejiang, China).

### TEM of liposomes

The morphology of liposomes was characterized by negative staining. Briefly, a carbon-coated copper grid (Zhongjingkeyi, China) was placed on filter paper. A 10 μl aliquot of the liposome suspension was deposited onto the grid and allowed to adsorb for 1 min. Excess liquid was gently removed by blotting with filter paper from the edge. Subsequently, 10 μl of 2% (w/v) uranyl acetate (TED PELLA, catalog no. 19481) was applied to the grid for negative staining for 1 min, and the excess stain was removed with filter paper. The grid was air-dried at room temperature for several minutes. Images were acquired using a transmission electron microscope (Hitachi HT7800, Japan) operated at an accelerating voltage of 80 to 120 kV.

### Liposome size and polydispersity index analysis

The hydrodynamic diameter and PDI of liposomes were determined by DLS using a Zetasizer Nano ZS90 (Malvern Instruments, UK). Liposome samples were diluted to an appropriate concentration (approximately 0.1 mg/ml) with deionized water and transferred into a disposable polystyrene cuvette. After equilibration at 25 °C for 120 s, measurements were performed in triplicate with 12-15 runs per measurement. The results were reported as the *Z*-average intensity mean diameter, and the PDI was used to assess the uniformity of the size distribution.

### Synthesis of HAMA

HAMA was synthesized by reacting hyaluronic acid (HA) with methacrylic anhydride in aqueous solution at pH 8.0, followed by dialysis and lyophilization. ^1^H NMR spectra (400 MHz, dimethyl sulfoxide-d6) confirmed successful grafting of methacrylate groups.

### AFM mechanical testing and Young’s modulus calculation of PLLA film

The local mechanical properties of PLLA films were characterized using AFM (Bruker Dimension Icon, USA) in force–distance curve mode. Silicon nitride probes (ScanAsyst-Air, nominal spring constant *k* = 0.4 N/m) were used, and the tip radius was calibrated using a standard tip characterization sample. Measurements were performed under ambient laboratory conditions (~25 °C). Force–distance maps were acquired over a 2 μm × 2 μm area using a 16 × 16 grid at a ramp rate of 1 Hz. At least 10 randomly selected regions were tested for each sample. Indentation depth was maintained below 10% of the film thickness to satisfy the assumptions of the Hertz contact model. Force curve data were analyzed using Nanoscope Analysis 1.9, and Young’s modulus (*E*) was calculated by fitting the contact portion of the approach curve using the Hertz model (spherical indenter approximation).

### Piezoelectric performance measurement of PLLA film

The piezoelectric output performance of the PLLA film was characterized using a custom-built ultrasonic stimulation system. The PLLA film was fixed on a sample stage, and ultrasound stimulation was applied at 40 kHz with an acoustic intensity of 0.33 W/cm^2^ via an ultrasonic transducer at power outputs of 0.1, 0.5, and 1 W. The open-circuit voltage generated across the film was recorded using a digital oscilloscope. Under each power condition, voltage values were recorded at 0, 5, and 10 s, with at least 3 repeated measurements at each time point. All measurements were performed at room temperature (25 °C). Data are presented as mean ± standard deviation (SD) (or as raw data points).

### XRD analysis of PLLA film

The crystal structure of the PLLA film was characterized using an x-ray diffractometer (Bruker D8 Advance). Measurements were performed with Cu Kα radiation (wavelength λ = 1.5406 Å) at an operating voltage of 40 kV and a current of 40 mA. Diffraction patterns were collected over a 2θ range of 10° to 50° with a step size of 0.02° and a scanning rate of 2°/min. The PLLA film sample was directly placed on a quartz sample holder, ensuring a flat surface for measurement. The obtained diffraction data were analyzed using the instrument’s proprietary software (Bruker DIFFRAC.EVA), and diffraction peaks were indexed by comparison with standard PDF cards (ICDD 00-054-1917).

### DSC analysis of PLLA film

The thermal properties of the PLLA film were characterized using a differential scanning calorimeter (TA Instruments Q2000). Approximately 5 to 10 mg of the PLLA film sample was placed in an aluminum pan and sealed with a lid, and an empty aluminum pan was used as a reference. Measurements were performed under a nitrogen atmosphere (flow rate, 50 ml/min) at a heating rate of 10 °C/min from 0 to 200 °C. The heat flow as a function of temperature was recorded.

### 3D printing of HAMA microspheres

HAMA microspheres were fabricated using digital light processing (DLP) 3D printing technology. HAMA (150 kDa) was dissolved in deionized water at a concentration of 2% (w/v) with 0.2% (w/v) lithium phenyl-2,4,5-trimethylbenzoylphosphinate (LAP) as the photoinitiator. The precursor solution was loaded into the resin tank of a DLP 3D printer (Azure-12, Cyberiad). Printing was performed in single-image static mode with a light intensity of 60 mW/cm^2^, an exposure time of 20 s per layer, and a layer thickness of 50 μm. These parameters were determined through pre-experimental optimization: Insufficient exposure (<20 s) resulted in fragile microspheres prone to fragmentation due to incomplete crosslinking, whereas excessive exposure (>20 s) caused shape distortion and over-crosslinking at the periphery, deviating from the intended spherical geometry. At the optimized 20s exposure, the microspheres exhibited stable spherical morphology, uniform porous structures, and sufficient mechanical integrity for subsequent processing. After printing, the microspheres were thoroughly washed with ethanol and deionized water to remove unreacted monomers, and finally freeze-dried for further use. Microsphere size distribution was analyzed using ImageJ software (version 2.0.2, National Institutes of Health (NIH)). A total of 280 microspheres were randomly selected from representative SEM images, and the equivalent circle diameter (ECD) was measured using the Analyze Particles function. The cumulative particle size distribution curve was plotted, and D10, D50, and D90 were calculated. The span value was calculated as (D90 − D10)/D50 to evaluate distribution width.

### Preparation of PLLA–HAMA composite microspheres

PLLA-HAMA composite microspheres were prepared by dip coating. PLLA (molecular weight 100,000 Da) was dissolved in dichloromethane at a concentration of 1% (w/v). Dried HAMA microspheres were immersed in the PLLA solution and dispersed with ultrasonic assistance for 5 min to allow thorough infiltration and coating of PLLA onto the microsphere surfaces. Subsequently, the solvent was slowly removed by rotary evaporation to solidify the PLLA layer on the HAMA microspheres. The obtained composite microspheres were washed 3 times with ethanol to remove loosely attached PLLA and dried under vacuum at room temperature. SEM cross-sectional imaging revealed a uniform PLLA shell with an average thickness of approximately 2-5 μm.

### SEM observation

The morphology of HAMA microspheres and PLLA-HAMA composite microspheres was observed using a scanning electron microscope (Hitachi S-4800). Observations were performed at various magnifications to capture both the overall morphology and surface microstructure of the microspheres.

### Loading of liposomes into HAMA microspheres

Porous HAMA microspheres were loaded with liposomes using a combined incubation-vacuum infiltration strategy. Liposomes were composed of hydrogenated soybean phosphatidylcholine (HSPC), cholesterol, and 1,2-distearoyl-sn-glycero-3-phosphoethanolamine (DSPE)-polyethylene glycol 2000 (PEG2000) at a molar ratio of 55:40:5, prepared by the thin-film hydration method. Glycine was encapsulated at an initial glycine-to-lipid ratio of 200 μg per mg of total lipid. Dried HAMA microspheres were immersed in the glycine-loaded liposome suspension and incubated to allow diffusion-driven penetration into the internal pore network. Samples were subsequently subjected to repeated vacuum cycles to enhance infiltration efficiency and ensure uniform distribution of liposomes throughout the microspheres. Encapsulation efficiency (EE%) and drug loading (DL%) were quantified using ultrafiltration centrifugation (3 kDa molecular weight cutoff, 10,000*g*, 30 min, 4 °C). Free glycine in the filtrate was measured by ultraviolet-visible (UV-Vis) spectroscopy at 570 nm following ninhydrin derivatization, using a glycine calibration curve (0-200 μg/ml, *R*^2^ > 0.999). EE% was calculated as (encapsulated glycine/total glycine added) × 100%, and DL% was calculated as (encapsulated glycine/total liposome mass) × 100%. The glycine-loaded liposomes exhibited an EE% of 96.46% and a DL% of 32.54%. Release kinetics were evaluated in PBS (pH 7.4, 37 °C). Ultrasound stimulation was applied during the release period to assess ultrasound-responsive release behavior.

### Fabrication of the Chinese microneedle system

The Chinese microneedle system was fabricated following the structural design of a previously developed heat-transfer Chinese microneedle [39], with modifications to enable microsphere loading. A screw-threaded groove was machined at the needle tip to provide an anchoring site for hydrogel microspheres. A triblock polymer adhesive previously developed in our laboratory was applied to the groove to serve as a coupling layer between the Chinese microneedle and the microspheres. Functionalized hydrogel microspheres were then mounted onto the coated groove, completing the construction of the Chinese microneedle system. For in vivo administration, the microneedle loaded with composite microspheres was inserted percutaneously into the NP region of the target disc under ultrasound guidance. Each disc received 5 μl of microsphere suspension containing PLLA-HAMA composite microspheres at 2 mg/ml, corresponding to approximately 10 μg of composite microspheres per disc. Based on the measured PLLA-to-HAMA ratio and glycine loading efficiency, this amount corresponds to ~10 μg of PLLA-HAMA microspheres, including ~2 μg of encapsulated glycine delivered per disc. Treatments were performed twice weekly for 4 consecutive weeks (8 total sessions). Ultrasound stimulation (40 kHz, 0.33 W/cm^2^, 10 min) was applied immediately after each administration in the ES and Dual-regulation groups.

### SA-β-gal staining

Senescence-associated β-galactosidase activity was detected using a commercial SA-β-gal staining kit (Beyotime, China). Cells were fixed and incubated with staining solution at 37 °C overnight. Blue-stained cells were counted under a light microscope, and the proportion of positive cells was calculated.

### EdU incorporation assay

Cell proliferation was assessed using the EdU assay kit (Abbkin, China). Cells were incubated with EdU (50 μM) for 2 h, fixed, and stained with Apollo dye. Nuclei were counterstained with Hoechst 33342. EdU-positive cells were quantified by fluorescence microscopy.

### Cytotoxicity assay

The cytotoxicity of the functionalized hydrogel microspheres was evaluated using live/dead cell staining and CCK-8 assays. Mouse macrophages (RAW264.7) and mouse NP cells (C57BL/6) were cocultured with the highest concentration of microspheres for 24, 72, and 120 h. At each time point, cells were gently washed with PBS and stained using a commercial live/dead cell staining kit (C2015S, Beyotime, China) following the manufacturer’s protocol. Fluorescence images were acquired using an inverted fluorescence microscope, where live cells were labeled in green and dead cells in red. The proportion of live cells was quantified using ImageJ software.

Cell metabolic activity was further assessed using CCK-8 (C0037, Beyotime, China). After coculture with microspheres for the indicated time points, 10% CCK-8 working solution was added to each well and incubated for 2 h at 37 °C. The absorbance at 450 nm was measured using a microplate reader. All values were normalized to the untreated control group to determine the effect of microspheres on cell proliferation. Each experiment was performed in triplicate.

### Western blot analysis

Proteins were extracted with radioimmunoprecipitation assay (RIPA) buffer containing protease inhibitors. Equal amounts of protein were separated by sodium dodecyl sulfate–polyacrylamide gel electrophoresis (SDS-PAGE) and transferred to polyvinylidene difluoride (PVDF) membranes. Membranes were blocked with 5% bovine serum albumin (BSA) and incubated with primary antibodies against p53 (GB115747, 1:500), p21 (GB155313, 1:500), p16^INK4a^ (ab211542, 1:1,000), TAF13 (GB111626, 1:500), BNIP3 (#DF8188, 1:500), MST1/2 (DF8569), phospho-MST1/2 (p-MST1/p-MST2, 28953-1-AP, 1:1,000), LATS1/2 (DF7517, 1:1,000), phospho-LATS1/2 (p-LATS1/p-LATS2, AF8163, 1:500), TAZ (A26425PM, 1:1,000), YAP (GB114152, 1:500), CTGF (25474-1-AP, 1:500), CYR61 (DF6250, 1:500), AGC1 (#64169, 1:500), ACTIN (GB15003, 1:5,000), glyceraldehyde-3-phosphate dehydrogenase (GAPDH) (GB15004, 1:3,000), GSDMD (GB114198, 1:3,000), cleaved caspase-1 (AF4005, 1:5,000), GLYRα1 (sc-293498, 1:3,000), DYSFERLIN (31658-1-ap, 1:3,000), and ANKRD1 (11427-1-AP, 1:1,500), followed by horseradish peroxidase (HRP)-conjugated secondary antibodies. Signals were visualized using enhanced chemiluminescence (ECL) reagent and quantified with ImageJ.

### Mitochondrial membrane potential (JC-1 assay)

Cells were incubated with JC-1 dye (Beyotime, China) for 20 min at 37 °C. Red (aggregates) and green (monomers) fluorescence were detected by confocal microscopy. The red/green ratio was calculated to evaluate mitochondrial membrane potential.

### ROS detection

Intracellular ROS levels were measured using DCFH-DA probe (Beyotime, China). Cells were incubated with 10 μM DCFH-DA for 30 min, washed, and imaged by fluorescence microscopy. Mean fluorescence intensity was quantified.

### Flow cytometry for cell cycle analysis

Cells were fixed in 70% ethanol overnight at -20 °C, treated with ribonuclease (RNase) A, and stained with PI. DNA content was analyzed by flow cytometry (BD FACSCanto II), and cell cycle distribution was determined using FlowJo (version 10.8.1) software.

### Immunofluorescence staining

Cells were fixed with 4% paraformaldehyde (PFA), permeabilized with 0.1% Triton X-100, and blocked with 5% BSA. Primary antibodies against COL II (ABclonal, A1560) and AGG (ABclonal, A8536) were incubated with the cells overnight at 4 °C. After washing, Alexa Fluor-conjugated secondary antibodies (Alexa Fluor 488: Abcam, ab150077; Alexa Fluor 647: Abcam, ab150075) were applied. Nuclei were counterstained with 4′,6-diamidino-2-phenylindole (DAPI) prior to imaging.

### Animal model of IVDD

Sprague-Dawley rats (11-month-old; *n* = 6 per group for histology; *n* = 3 per group for longitudinal imaging; sex-balanced) were obtained from the Animal Experimental Center at Hangzhou Medical College (Zhejiang, China). Animals were randomly assigned to experimental groups using a random number table method before any surgical procedure. Following a 1-week acclimatization period, a caudal IVDD model was established under general anesthesia with intraperitoneal administration of sodium pentobarbital (30 mg/kg). For postoperative analgesia, buprenorphine (0.05 mg/kg, subcutaneous) was administered every 12 h for the first 48 h post-surgery. For imaging sessions (x-ray and MRI), animals were anesthetized with 2% isoflurane in oxygen via inhalation. After disinfection with povidone-iodine, a 21-gauge needle was percutaneously inserted into the center of coccygeal discs, rotated 360°, retained for 30 s, and then withdrawn to induce structural disruption. All surgical procedures were performed under sterile conditions. Animals were housed in a specific pathogen-free (SPF) facility under controlled environmental conditions (22-24 °C, 40%-70% humidity, 12-h light/dark cycle) with unrestricted access to food and water. All experimental protocols were conducted in accordance with institutional and national guidelines for the care and use of laboratory animals. For outcome assessment, all x-ray, MRI, and histological samples were coded with random numbers by a researcher not involved in the analysis. Radiological assessments [disc height index (DHI) and T2 signal intensity] and histological scoring were performed independently by 2 trained observers who were blinded to group allocation, and the mean values were used for statistical analysis. Animals were excluded from the study if they exhibited (a) signs of infection at the injection site, (b) tail autotomy, or (c) failure of the puncture procedure (confirmed by lack of disc space narrowing on post-operative x-ray). No animals met these exclusion criteria during the study period.

### Coculture of NP cells and macrophages with noncontact ES

Senescent mouse NP cells (C57BL/6, passage 3, designated as P3) were induced by exposure to d-galactose (8 mg/ml) and passaged 3 times prior to experimentation. Mouse macrophages (RAW264.7, passage 3) were used for coculture. Cells were subcultured every 2 to 3 d using 0.25% trypsin for dissociation. For coculture, NP cells were seeded in the lower compartment of Transwell inserts (Costar, 3640) at a density of 1 × 10^5^ cells/well, while macrophages were seeded in the upper chamber at 5 × 10^4^ cells/well. Unless otherwise specified, cells were maintained in DMEM supplemented with 10% heat-inactivated FBS and 1% penicillin-streptomycin, in a humidified incubator at 37 °C with 5% CO_2_. We performed a proof-of-concept experiment using a YC-3B biphasic programmable stimulator (version 2; Beijing Jiahhang Bochuang Technology Co. Ltd., China). Stimulation parameters were as follows: output mode, timed direct current; pulse polarity, positive; intensity, 2 mA; stimulation duration, 1,800 s; triggering mode, internal; synchronization signal, rising edge. These parameters were selected based on previous literature and pilot studies [40]. This experiment was performed once to establish equivalence between direct ES and the microsphere-based system. The results are presented in Fig. S3E, showing that YC-3B stimulation similarly reduced the proportion of SA-β-gal-positive senescent NP cells, comparable to the effect of the microsphere + ultrasound system.

For all subsequent experiments, including the mechanistic studies (Figs. 3 to 7) and in vivo studies (Fig. 8), the PLLA-HAMA composite microspheres were activated by ultrasound to generate localized piezoelectric stimulation. Specifically, microspheres were added to the culture system at a concentration of 2 mg/ml, and ultrasound was applied at 40 kHz with an acoustic intensity of 0.33 W/cm^2^ for 10 min per session using an ultrasonic bath (Branson 2800 CPX series). Stimulation was performed once every 12 h, followed by continued culture for an additional 6 h after the final stimulation. This modality represents the actual therapeutic platform and was used for all experiments unless otherwise noted. Throughout the manuscript, “ES” in figures and text refers to this piezoelectric stimulation via ultrasound-activated PLLA-HAMA microspheres, unless explicitly stated as the YC-3B proof-of-concept experiment.

### Cell culture and treatments

In the cell culture experiment to assess the anti-senescence effect of morroniside and the involvement of YAP/TAZ signaling, NP cells (C57BL/6, P3) were divided into 4 groups: young NP cells (C), aging NP cells (S), aging NP cells treated with morroniside (S-M), and aging NP cells treated with morroniside plus YAP/TAZ inhibitor (S-MY). For drug treatment, NP cells were incubated with 100 μM morroniside for 24 h, while YAP-TAZ signaling was inhibited by pretreatment with 1 μM YAP-TAZ inhibitor-1 for 24 h.

In the negative control group, cells were maintained under standard culture conditions without stimulation. In the model control group, cells were pretreated with LPS (1 μg/ml) for 4 h, followed by nigericin (10 μM) for 30 min to induce classical inflammasome activation. In the vita-field group, cells received LPS pretreatment (1 μg/ml, 4 h) and were subsequently cotreated with nigericin (10 μM) and glycine (5 mM) to evaluate the protective effect of glycine against pyroptosis. In the GlyR antagonist group, cells were treated with LPS (1 μg/ml, 4 h), nigericin (10 μM), glycine (5 mM), and the GlyR antagonist strychnine (10 μM) to assess receptor dependency. In the membrane repair inhibitor group, cells were treated with LPS (1 μg/ml, 4 h), nigericin (10 μM), glycine (5 mM), and BAPTA-AM (10 μM), an intracellular calcium chelator, to block calcium-dependent membrane repair. In the resveratrol group, cells were directly exposed to resveratrol (20 μM) to examine metabolic activation effects. Finally, in the resveratrol + glycine group, cells were simultaneously treated with resveratrol (20 μM) and glycine (5 mM) to evaluate potential synergistic protective effects.

### Quantitative real-time PCR

Total RNA was extracted using an RNA extraction reagent (G3013, Saiweier Biotechnology, China) according to the manufacturer’s protocol. Complementary DNA (cDNA) was synthesized using the SweScript All-in-One RT SuperMix for qPCR (G3337, Saiweier Biotechnology). Quantitative real-time PCR was performed using the 2× Universal Blue SYBR Green qPCR Master Mix (G3326, Saiweier Biotechnology) on a CFX Connect Real-Time PCR Detection System (Bio-Rad, USA). Actin was used as the internal reference gene for normalization. Primer sequences are listed in Fig. S7.

### Bulk RNA-seq library preparation and analysis

Total RNA was isolated from mouse NP cells (C57BL/6, P3) using a standard phenol-based extraction protocol, followed by integrity and purity assessment via NanoPhotometer (OD260/280 and OD260/230), Qubit 2.0 Fluorometer, and Agilent 2100 Bioanalyzer. Samples with RNA integrity number (RIN) > 7.0 were processed for library construction. Ribosomal RNA was selectively depleted using a commercial rRNA removal kit. Enriched polyadenylated mRNA was fragmented under optimized conditions and reverse-transcribed into cDNA using random primers. After RNA degradation, second-strand synthesis was performed with DNA polymerase I. Double-stranded cDNA underwent end-repair, dA-tailing, and ligation with indexed adapters. Fragment selection (~200 base pairs (bp)) was conducted with AMPure XP beads, followed by limited-cycle PCR amplification. Final libraries were purified and quality-controlled prior to sequencing. Libraries were sequenced on an Illumina NovaSeq 6000 platform with paired-end 150-bp reads. Raw sequencing data underwent initial quality assessment using FastQC (v0.11.9) with default parameters. Adapter sequences and low-quality bases were removed using Cutadapt (v4.4). High-quality reads were subsequently aligned to the mouse reference genome (Mus musculus, Ensembl release 113) using STAR (v2.7.10a). Gene-level quantification was performed using featureCounts (v2.0.1). Differential expression analysis was conducted with DESeq2 (v1.38.3) implemented in R (v4.2.2), and genes with adjusted *P* value < 0.05 were considered significantly differentially expressed. Downstream functional enrichment analyses, including GO and KEGG pathway annotation, were carried out using ClusterProfiler (v4.4.4).

### Untargeted metabolomics

#### Chemicals and reagents

Methanol (LC-MS grade, lot no. 1230, CAS: 67-56-1), acetonitrile (lot no. 1646, CAS: 75-05-8), ammonium acetate (lot no. 1350K100, CAS: 631-61-8), and ammonium hydroxide (CAS: 1336-21-6) were purchased from CNW Technologies (ANPEL Laboratory Technologies, Shanghai, China). LC-MS grade water was obtained from a Milli-Q purification system (Merck Millipore, Germany). The internal standard 2-chloro-l-phenylalanine (CAS: 103616-89-3, ≥98% purity) was purchased from Hengbai Biotechnology (Shanghai, China).

#### Instrumentation

Metabolomic profiling was performed using an Agilent 1290 UHPLC system coupled with a Q Exactive Orbitrap mass spectrometer (Thermo Fisher Scientific, USA). Chromatographic separation was achieved using an ACQUITY UPLC HSS T3 column (1.7 μm, 2.1 × 100 mm; Waters). Additional instruments included JXFSTPRP-24 tissue homogenizer (Shanghai Jingxin Technology), Heraeus Fresco17 centrifuge (Thermo Scientific), VORTEX-5 mixer (QLB Instruments), BSA124S-CW balance (Sartorius, Beijing), PS-60AL ultrasonic bath (Redbond Electronics, Shenzhen), and NanoPhotometer spectrophotometer.

#### Metabolite extraction

For each sample, 100 μl of biological fluid was mixed with 300 μl of methanol and 20 μl of internal standard. After vortexing for 30 s, samples were sonicated in an ice-water bath for 5 min, followed by incubation at -20 °C for 2 h. Precipitated proteins were removed by centrifugation at 13,000 rpm for 15 min at 4 °C. A 200 μl aliquot of the supernatant was transferred to a 2 ml autosampler vial for LC-MS analysis. Quality control (QC) samples were prepared by pooling equal volumes of each experimental extract. One QC sample was injected every 6 to 10 runs to monitor system reproducibility and signal stability.

#### LC-MS conditions

In positive ion mode, mobile phase A consisted of 0.1% formic acid in water and mobile phase B was acetonitrile. In negative ion mode, mobile phase A was 5 mM ammonium acetate in water (pH adjusted to 9.0 with ammonium hydroxide); mobile phase B was acetonitrile. The injection volume was 1 μl. 

#### MS settings

Full MS and data-dependent MS/MS (ddMS2) scans were acquired using electrospray ionization (ESI) in both positive (spray voltage: +3,800 V) and negative (-3,100 V) modes. Additional parameters: capillary temperature 320 °C, sheath gas flow 45 Arb, auxiliary gas flow 15 Arb, scan range 70 to 1,000 *m*/*z*, resolution 70,000 (MS1) and 17,500 (MS2), stepped collision energy at 20, 40, and 60 eV, scan rate 7 Hz. QC samples were analyzed using full MS-ddMS2 mode across 4 acquisition windows (70 to 200, 190 to 400, 390 to 600, 590 to 1,000 *m*/*z*), each scanned once to maximize MS/MS coverage for compound identification.

#### Data preprocessing

Raw MS data were converted to .MzML format using ProteoWizard and processed using XCMS for peak alignment, retention time correction, and peak area extraction. The following parameters were used for peak picking: centWave *m*/*z* = 10 ppm, peakwidth = c (10, 60), prefilter = c (10, 100). For peak grouping: bw = 5, mzwid = 0.025, minfrac = 0.5. Features with >50% missing values in any group were filtered out, and remaining missing values were imputed using KNN. Features with relative standard deviation (RSD) > 50% in QC samples were excluded from downstream analysis.

#### Metabolite annotation

Compound identification was performed by comparing the accurate *m*/*z* values (mass error < 10 ppm) and MS/MS spectra against an in-house database established by the service provider, which contains high-resolution mass and MS/MS spectral information from authentic standards compiled from public databases including Human Metabolome Database (HMDB), the Metabolite and Chemical Entity Database (METLIN), and KEGG.

#### Annotation confidence levels

Following the MSI guidelines, we have assigned confidence levels to all key metabolites (Table 1).

**Table 1.** Confidence levels for metabolite identification based on MSI guidelines

MSI level	Definition
Level 1	Confirmed by authentic standards (retention time, accurate mass, and MS/MS matching)
Level 2	Putatively annotated compounds (accurate mass with mass error < 10 ppm and MS/MS spectral matching to database)
Level 3	Putatively characterized compound classes (accurate mass only)

#### GO enrichment analysis for metabolites

To perform GO analysis on metabolites, we mapped the annotated metabolites to KEGG compound IDs, then to corresponding KEGG genes/enzymes, and finally to GO biological process terms. The enrichment analysis employed a hypergeometric distribution-based over-representation test, and *P* values were corrected for multiple testing using the Benjamini-Hochberg method to control the FDR at <0.05. All *P* values were adjusted using the Benjamini-Hochberg procedure, and metabolites with FDR < 0.05 and |log_2_FC| ≥ 1 were considered significantly differential. Blank medium controls (without cells), serum controls, and cell-free controls were included in the metabolomic workflow. Features detected in these controls were excluded from differential analysis to eliminate false-positive annotations from media components or serum contaminants.

### Single-cell library preparation and sequencing

Rat (Sprague-Dawley) lumbar intervertebral disc tissues were harvested from 8-week-old rats for the Young group, and from 11-month-old rats for the Aging and ES groups, which were washed twice with ice-cold PBS. The samples were digested in 6 ml of 0.25% trypsin-EDTA at 37 °C with gentle agitation (100 rpm) for 10 min. The digestion was terminated by adding an equal volume of cold PBS containing 10% FBS, followed by 2 additional washes with PBS. The resulting cell suspension was sequentially filtered through 70- and 30μm strainers to remove tissue debris. Two cellular fractions were collected and treated with 4 ml of red blood cell lysis buffer to eliminate erythrocytes. Cells were resuspended in 1× PBS containing 0.04% BSA and then centrifuged twice at 300*g* for 3 min at 4 °C. Cell viability (>85%) was confirmed via trypan blue exclusion, and total cell count was measured using an automated cell counter.

Single-cell suspensions were loaded into the 10x Chromium Controller using the Chromium Single Cell 3′ Reagent Kit v3 (10x Genomics), targeting approximately 15,000 to 20,000 cells per sample, consistent with the final estimated cell counts. Reverse transcription, cDNA amplification, and library preparation were performed following the manufacturer’s protocol. Libraries were sequenced on an Illumina NovaSeq 6000 platform (paired-end, 150 bp) by LC-Bio Technology, with a minimum read depth of 20,000 reads per cell.

### Single-cell data analysis

Raw sequencing data were processed using Cell Ranger (version 3.1.0, 10x Genomics) with default parameters for demultiplexing, alignment to the rat reference genome (rattus_norvegicus-102), and generation of gene-cell count matrices. QC filtering was performed to remove cells with fewer than 200 detected genes or with mitochondrial gene percentages greater than 10%. Data normalization and variance stabilization were performed using the SCTransform function in Seurat (version 5.0.1). Batch effects among samples were corrected using Harmony (version 1.2.4) integrated with Seurat. PCA was applied for dimensionality reduction, and the first 30 principal components were used for downstream analyses. UMAP was used for visualization. Cells were clustered using the graph-based Louvain algorithm implemented in Seurat with a resolution of 0.5.

Cell-type annotation was performed based on canonical marker genes and publicly available reference datasets. Differential gene expression analysis was conducted using the Wilcoxon rank-sum test, with genes considered differentially expressed if |log₂FC| ≥ 0.25 and adjusted *P* value < 0.05 (Benjamini-Hochberg correction). Pathway enrichment analysis was performed using GO and KEGG via the ClusterProfiler package (version 4.16.0). All statistical analyses were performed in R (version 4.3.2) using RStudio. The “Repair score” for macrophages was calculated using the AddModuleScore function from the Seurat package. This function computes a module score for each cell as the mean log-normalized expression of a curated gene set, subtracted by the aggregated expression of control feature sets randomly selected from expression bins. The repair gene set was curated based on previously published macrophage repair signatures [41–43] and included the following genes: Vegfa, Tgfb1, Mertk, and Il10. Per-cell module scores were then scaled (*z*-scored) across all macrophages to enable comparison across conditions.

### Flow cytometry for apoptosis

Apoptosis was assessed using the Annexin V-FITC/PI Apoptosis Detection Kit (BD Biosciences, catalog no. 556547) according to the manufacturer’s protocol. Briefly, NP cells were harvested, washed twice with cold PBS, and resuspended in 1× binding buffer at a concentration of 1 × 10^6^ cells/ml. Cells were stained with 5 μl of Annexin V–fluorescein isothiocyanate (FITC) and 5 μl of PI for 15 min at room temperature in the dark. Samples were analyzed within 1 h using a BD FACSCanto II flow cytometer (BD Biosciences) equipped with 488- and 561-nm lasers. For each sample, at least 10,000 events were acquired. Data were processed using FlowJo software (version 10.8, BD Biosciences). Early apoptotic cells were defined as Annexin V^+^/PI^−^, late apoptotic cells as Annexin V^+^/PI^+^, and necrotic cells as Annexin V^−^/PI^+^. The total apoptotic cell proportion was calculated as the sum of early and late apoptotic populations.

### X-ray and MRI imaging

Rats (Sprague-Dawley, 11-month-old) were anesthetized with 2% isoflurane in oxygen prior to imaging. X-ray radiographs were obtained at 0, 2, 4, 6, and 8 weeks using a digital x-ray imaging system with exposure settings of 40 kV and 1 mAs. DHI was calculated as the ratio of disc height to adjacent vertebral body height using ImageJ (version 2.0.2, NIH).

T2-weighted sagittal images of the coccygeal were acquired using a 7.0-T small-animal MRI scanner (PharmaScan 70/16, Bruker BioSpin GmbH, Germany). Imaging was performed with a rapid acquisition with relaxation enhancement (RARE) sequence using the following parameters: repetition time (TR)/echo time (TE) = 2,500/33 ms, echo spacing = 8.25 ms, RARE factor = 8, averages = 6, field of view = 70 × 35 mm, matrix size = 256 × 256, and slice thickness = 0.8 to 1.2 mm. Signal intensity was measured within the NP region, and normalized T2 signal intensity was calculated as the ratio of disc signal to adjacent cerebrospinal fluid signal.

### Histological staining

Rats (Sprague-Dawley, 11-month-old) were euthanized, and caudal intervertebral discs were harvested, fixed in 4% PFA for 48 h at 4 °C, decalcified in 10% EDTA (pH 7.4) for 4 weeks, and embedded in paraffin. Sections (5 μm thickness) were cut sagittally using a microtome.

#### Safranin O-Fast Green staining

Sections were deparaffinized, rehydrated, and stained with Weigert’s hematoxylin for 5 min, followed by Fast Green FCF (0.04% w/v) for 5 min, and then Safranin O (0.1% w/v) for 10 min. GAG content was visualized as red staining and quantified as the percentage of Safranin O-positive area relative to the total disc area using ImageJ.

#### Immunohistochemistry

For AGG and COL II detection, sections were subjected to antigen retrieval with pepsin (0.4% w/v in 0.01 M HCl) for 30 min at 37 °C. Endogenous peroxidase activity was blocked with 3% H_2_O_2_ for 10 min. Sections were incubated overnight at 4 °C with primary antibodies against AGG (Abcam, ab3778, 1:150) and COL II (Abcam, ab34712, 1:150). After washing, sections were incubated with an HRP-conjugated secondary antibody (PV-6001, ZSGB-BIO) for 1 h at room temperature. Immunoreactivity was visualized using a 3,3′-diaminobenzidine (DAB) substrate, followed by hematoxylin counterstaining. Positive staining areas were quantified using ImageJ as described above.

#### Masson’s trichrome and H&E staining

For structural assessment, sections were stained with Masson’s trichrome using a commercial staining kit (Solarbio, G1340) according to the manufacturer’s protocol. For H&E staining, sections were stained with hematoxylin for 5 min and eosin for 2 min.

### Cytotoxicity and inflammasome assays

Supernatants were collected and the concentrations of IL-1β, IL-6, and TNF-α were determined using commercial enzyme-linked immunosorbent assay (ELISA) kits (DUMABIO, catalog no. DM-X5495 for IL-1β, catalog no. DM-X5501 for IL-6, and catalog no. DM-K126 for TNF-α) according to the manufacturer’s instructions. Caspase-1 activity was measured using the Caspase-1 Activity Assay Kit (Abbkin, catalog no. KTA3020), with absorbance at 405 nm used to calculate *p*-nitroaniline release. LDH release was assessed using the LDH Cytotoxicity Assay Kit (Beyotime, catalog no. C0016), with corrected optical density (OD) values normalized to maximum lysis controls. Plasma membrane permeability was evaluated by YO-PRO-1 uptake (Beyotime, catalog no. C1356S), and fluorescence intensity was quantified by microscopy analysis.

### Targeted LC-MS/MS quantification

Morroniside concentrations in macrophage supernatants were determined using an AB Sciex Triple Quad 6500 LC-MS/MS system operated in MRM mode. Absolute quantification was performed with an external calibration curve generated from authentic morroniside standards.

### GSDMD inhibition assay

To assess whether metabolite release depends on GSDMD pore formation, macrophages were pretreated with disulfiram (100 μM, 2 h) prior to activation with NP-conditioned medium. Supernatants were subjected to targeted LC-MS/MS quantification.

### High-resolution MS

The candidate metabolite was analyzed on a high-resolution mass spectrometer to determine its exact molecular formula and fragmentation pattern. Data were compared with authentic morroniside standards under identical LC-MS conditions to evaluate structural similarity.

### Statistical analysis

Statistical analyses were conducted using GraphPad Prism (version 10) and R. Coding and data visualization were performed in RStudio (version 4.5.0). All quantitative data are presented as mean ± SD. Statistical comparisons between groups were performed using either Student’s *t* test or one-way analysis of variance (ANOVA), as appropriate. Differences were considered statistically significant at *P* < 0.05. Significance levels are denoted as follows: *P* < 0.05 (*), *P* < 0.01 (**), *P* < 0.001 (***), and *P* < 0.0001 (****). *P* values greater than 0.05 were considered not significant (ns). For longitudinal imaging datasets (x-ray DHI and MRI T2 signal intensity measured at 0, 2, 4, 6, and 8 weeks), 2-way repeated-measures ANOVA was used with Group and Time as factors, followed by Tukey’s post hoc tests. This model accounts for correlations among repeated measurements within the same animal.

## Ethical Approval

The animal experiments were approved by the Animal Ethics Committee of the Second Affiliated Hospital of Zhejiang University School of Medicine (ethics approval no. 2024-351).

## Data Availability

The data that support the findings of this study are available from the corresponding authors upon reasonable request.
