# Supplementary material for: Positioning Creation of Cell Communication Channels via Chinese Microneedle
Source: Research (Wash D C). 2026 Oct 27;9:1459. doi: 10.34133/research.1459 (PMC13710764; doi:10.34133/research.1459)
Supplement: Supplementary file 1 [file research.1459.f1.pdf]

Positioning creation of cell communication channels via Chinese microneedle

Yiwen Xu [1, 2, 3, 4, 5, 6]†, Miaojie Fang[1, 2, 3, 4, 5, 6]†, Zhenzhong Chen[1, 2, 3, 4, 5, 6], Hong Liu[1, 2, 3, 4, 5, 6], Yucheng Xue[1, 2, 3, 4, 5, 6], Zilong Li[1, 2, 3, 4, 5, 6], Yue Lin[1, 2, 3, 4, 5, 6], Kelei Wang[1, 2, 3, 4, 5, 6], Feng Lin [1, 2, 3, 4, 5, 6 *], Ning Zhang [1, 2, 3, 4, 5, 6 *]

[1] Department of Orthopedic Surgery, The Second Affiliated Hospital, Zhejiang University School of Medicine, Hangzhou 310009, China

[2] Orthopedics Research Institute of Zhejiang University, Hangzhou 310009, China

[3] Key Laboratory of Motor System Disease Research and Precision Therapy of Zhejiang

Province, Hangzhou 310009, China

[4] Zhejiang Provincial Clinical Medical Research Center for Motor System Diseases, Hangzhou

310009, China

[5] International Chinese Musculoskeletal Research Society, Hangzhou 310009, China

[6] State Key Laboratory of Transvascular Implantation Devices, Hangzhou 310009, China

*Corresponding author. Email: zhangning@zju.edu.cn (N. Z.); linfeng2023@zju.edu.cn (F. L.)

†These authors contributed equally to this work.

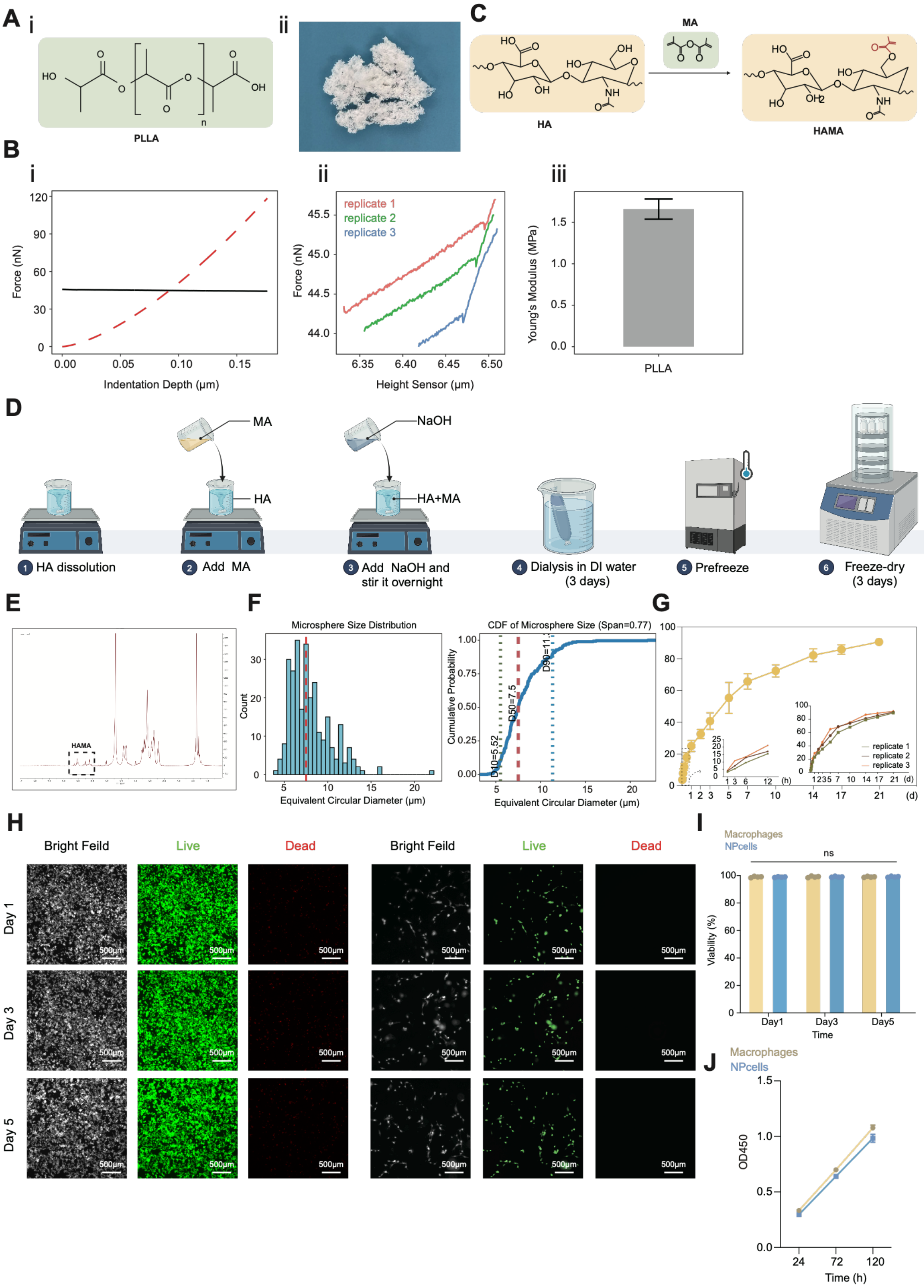

Supplementary Figure 1. Structural and mechanical characterization of HAMA and PLLA.

(A) (i) Chemical formula of PLLA; (ii) Photograph of PLLA film.

(B) (i) Representative Hertz fitting curve obtained from AFM indentation of PLLA film; (ii) Triplicate AFM measurements; (iii) Statistical analysis of Young's modulus values, showing an average modulus of 1.66 GPa.

(C) Chemical structure of HAMA, synthesized by conjugating hyaluronic acid (HA) with methacrylic anhydride (MA).

(D) Schematic illustration of the preparation process of HAMA microspheres.

(E) ^1H NMR spectrum of HAMA, confirming successful methacrylation of HA.

(F) Size distribution analysis of 3D-printed HAMA microspheres (n=280). Left, histogram showing frequency distribution of equivalent circle diameters (ECD) with D50 indicated (red dashed, 7.50 μm). Right, cumulative particle size distribution curve with D10 (green dashed, 5.52 μm), D50 (red dashed, 7.50 μm), and D90 (blue dashed, 11.33 μm). Span = 0.77, indicating a narrow and uniform size distribution.

(G) Cumulative release was measured in PBS (pH 7.4, 37 °C) over 21 days.

(H) Live/dead staining of macrophages and nucleus pulposus cells co-cultured with the highest concentration of functionalized hydrogel microspheres for 24 h, 72 h, and 120 h. Live cells are shown in green and dead cells in red. No obvious dead-cell aggregation was observed at any time point.

(I) Quantification of live-cell ratios at different time points, showing consistently high cell viability with no significant differences among groups (n=3).

(J) CCK-8 assay results of macrophages and nucleus pulposus cells after co-culture with microspheres for 24 h, 72 h, and 120 h, indicating that the microspheres do not inhibit cell proliferation at the maximum working concentration.

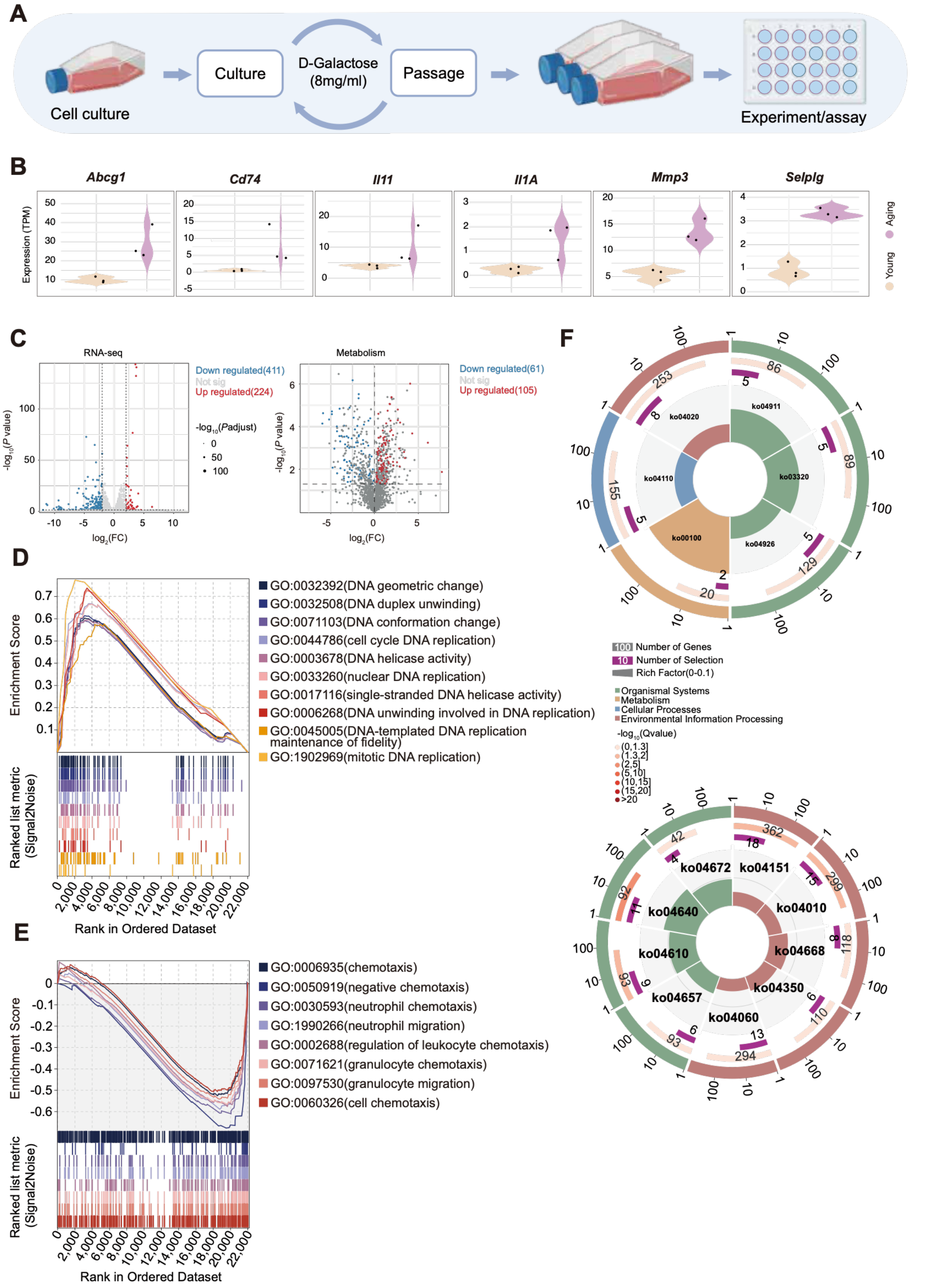

Supplementary Figure 2. Transcriptomic and metabolomic profiling reveals molecular reprogramming of senescent nucleus pulposus cells by electrical stimulation.

(A) Experimental scheme of the Aging NP model and electrical stimulation protocol.

(B) Violin plots showing the distribution of transcript levels (TPM) for representative genes

(*Abcg1, Cd74, Il11, Il1a, Mmp3, Selplg*) in young nucleus pulposus cells (Y-NPCs, beige) and aged nucleus pulposus cells (A-NPCs, purple) (n=3).

(C) Volcano plots of transcriptomic (left, n=3 biological replicates) and metabolomic (right, n=3 biological replicates) data comparing Aging and ES groups. Red dots represent significantly upregulated genes/metabolites in the ES group, blue dots represent significantly downregulated genes/metabolites ($|\log_2 FC| \geq 1$, FDR < 0.05).

(D) GSEA showing significantly enriched upregulated pathways in the ES group (ES vs Aging). Pathways include cell cycle, DNA replication, and oxidative phosphorylation (FDR < 0.05, NES > 1).

(E) GSEA showing significantly enriched downregulated pathways in the ES group (ES vs Aging). Pathways include senescence-associated secretory phenotype (SASP), inflammatory response, and TGF-β signaling (FDR < 0.05, NES < -1).

(F) Enrichment map circles showing upregulated (top) and downregulated (bottom) KEGG pathways in ES vs Aging.

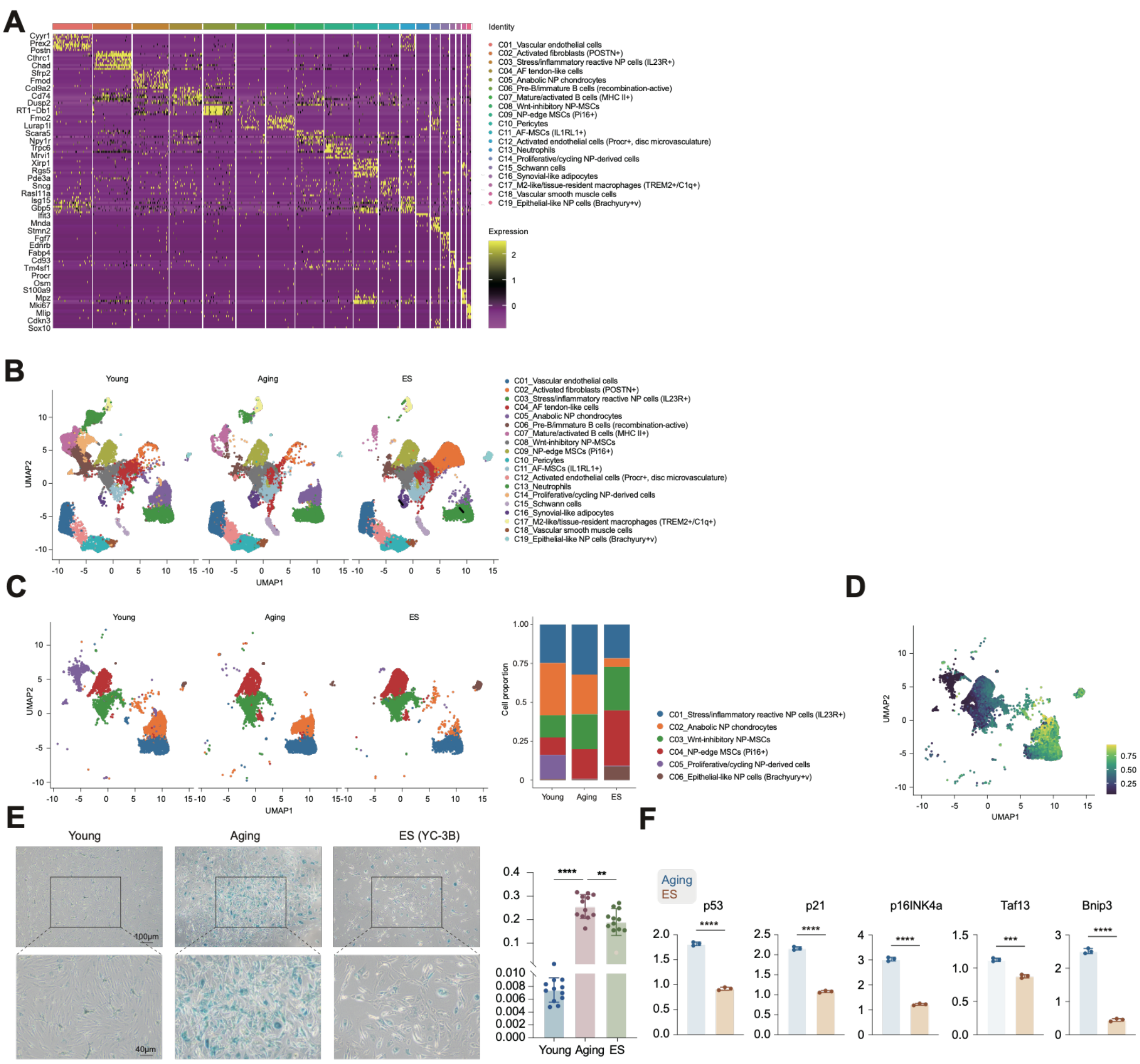

Supplementary Figure 3. Cell type identification and NP subcluster distribution across Young, Aging, and ES groups.

(A) Heatmap showing expression of signature markers for each cell type.

(B) UMAP plots of all cell clusters from intervertebral disc tissue in Young, Aging, and ES groups.

(C) UMAP plots highlighting NP cell-related clusters across Young, Aging, and ES groups.

(D) CytoTRACE score of each state of NP cells.

(E) Proof-of-concept validation using YC-3B external electrical stimulation. SA-β-gal staining (left) and quantitative histogram (right) in Young, Aging, and YC-3B stimulation groups (n = 12). Senescent NP cells received YC-3B electrical stimulation (2 mA, 1800 s) or no treatment.

(F) Western blot quantification of aging-related and matrix synthesis proteins (n=3).

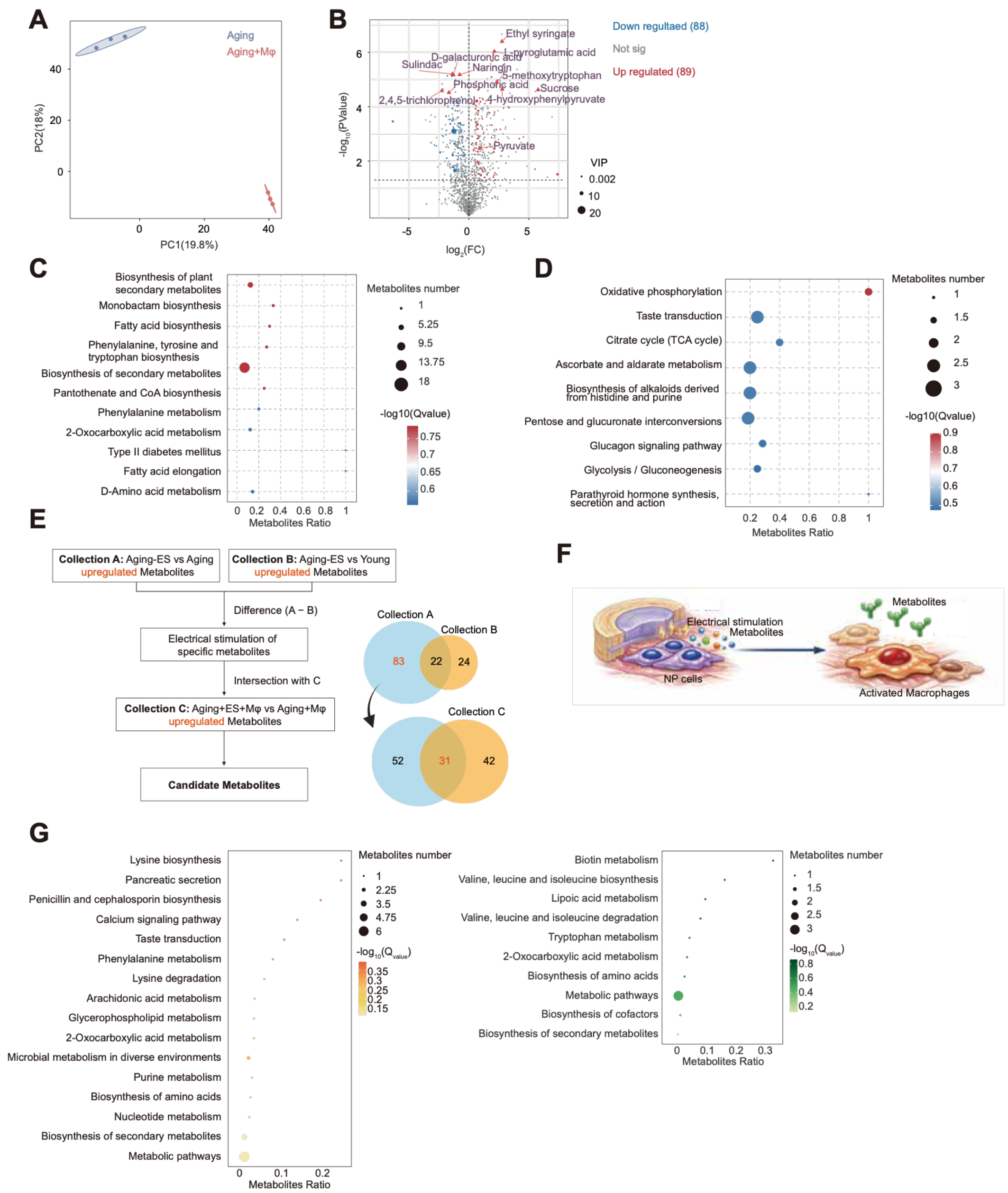

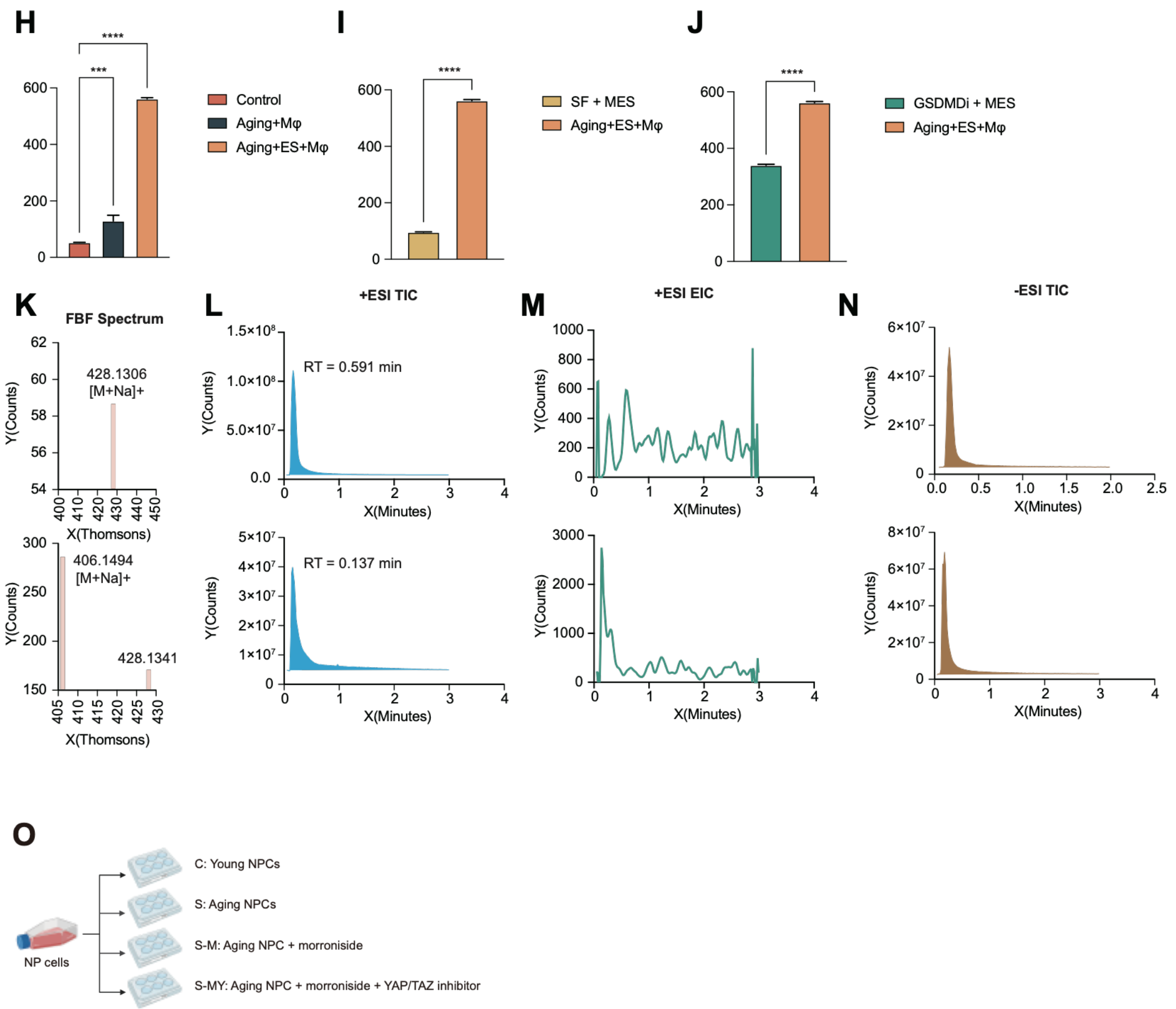

Supplementary Figure 4. Metabolomic analysis of synergistic regulation of macrophage polarization by microsphere-treated nucleus pulposus cells

(A) PCA analysis showing metabolic profile separation between aging group and aging NPC+macrophage co-culture group, indicating significant metabolic remodeling induced by co-culture.

(B) Volcano plot of differential metabolites between aging and aging NPC+macrophage co-culture groups. Red dots represent significantly upregulated metabolites after co-culture, blue dots represent significantly downregulated metabolites ($|log_2FC| \geq 1$, FDR < 0.05).

(C) GO enrichment analysis of upregulated metabolites. Metabolites upregulated in the co-culture group are primarily enriched in anti-inflammatory and repair-related pathways.

(D) GO enrichment analysis of downregulated metabolites. Metabolites downregulated in the co-culture group are primarily enriched in pro-inflammatory and oxidative stress-related pathways.

(E) Flowchart of metabolite screening strategy, illustrating the analytical workflow from differential metabolite identification to candidate metabolite selection.

(F) ES-activated NP cells release metabolites that prime macrophage activation, which in turn secrete feedback metabolites.

(G) GO enrichment bubble plot. Red bubbles (left) represent pathways upregulated in MES compared to MN, blue bubbles (right) represent pathways downregulated in MES.

(H) Absolute concentrations of morroniside-like metabolite (ng/mL) determined by external standard LC-MS/MS MRM in supernatants of untreated macrophages (Control), macrophages treated with conditioned medium from non-stimulated NP cells (Aging+Mφ), and macrophages treated with conditioned medium from electrically stimulated NP cells (Aging+ES+Mφ) (n=3).

(I) Morroniside-like metabolite concentrations in macrophages activated under serum-free (SF + MES) versus serum-containing (Aging+ES+Mφ) conditions (n=3).

(J) Morroniside-like metabolite concentrations in macrophages activated with or without disulfiram (GSDMD inhibitor, 2 h pretreatment) (n=3).

(K) Full-scan spectra showing the $[M+Na]^+$ ion at m/z 428.13 detected in both the candidate metabolite and authentic morroniside standard, consistent with the theoretical molecular formula $C_{17}H_{25}O_{11}Na$. Mass errors for the candidate were 5.98 ppm ($[M+H]^+$) and 12.17 ppm ($[M+Na]^+$).

(L-M) Total ion chromatograms (+ESI) and extracted ion chromatograms (EIC, m/z 428.13) of the morroniside standard (top) and the candidate metabolite (bottom) (L-M), and total ion chromatogram (-ESI) of the candidate (N). The standard and candidate exhibited distinct retention times (0.591 min vs. 0.137 min), whereas both shared the $[M+Na]^+$ ion at m/z 428.13, confirming the molecular formula $C_{17}H_{25}O_{11}$.

(O) NP cells were divided into four groups: young (C), senescent (S), senescent treated with authentic morroniside (S-M), and senescent treated with authentic morroniside plus a YAP/TAZ inhibitor (S-MY).

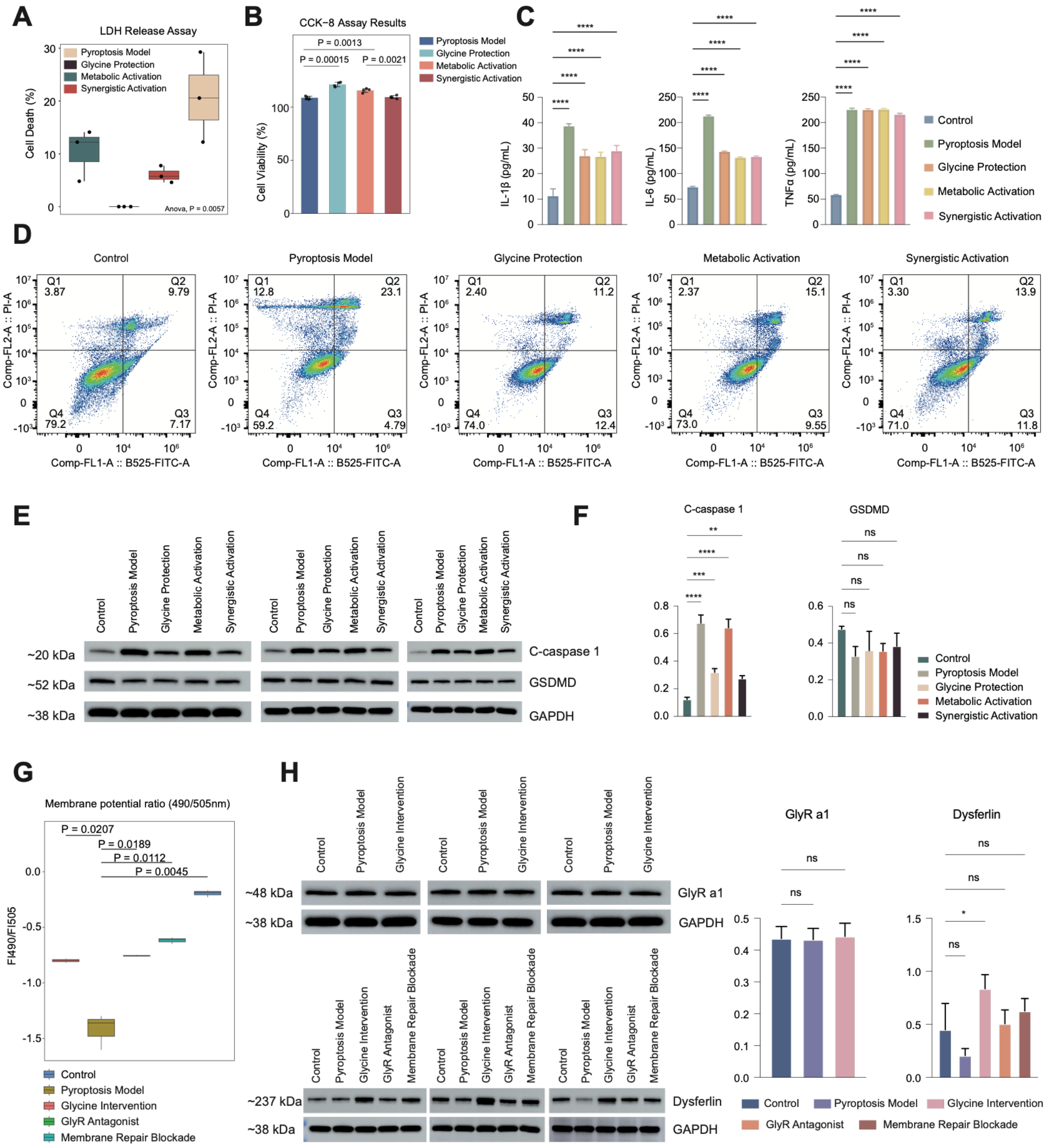

A
LDH Release Assay
Pyroptosis Model
Glycine Protection
Metabolic Activation
Synergistic Activation
Cell Death (%)
Anova, P = 0.0057
B
CCK-8 Assay Results
P = 0.0013
P = 0.00015
P = 0.0021
Cell Viability (%)
Pyroptosis Model
Glycine Protection
Metabolic Activation
Synergistic Activation
C
IL-1β (pg/mL)
IL-6 (pg/mL)
TNFα (pg/mL)
Control
Pyroptosis Model
Glycine Protection
Metabolic Activation
Synergistic Activation
D
Control
Pyroptosis Model
Glycine Protection
Metabolic Activation
Synergistic Activation
Comp-FL2-A :: PI-A
Comp-FL1-A :: B525-FITC-A
E
Control
Pyroptosis Model
Glycine Protection
Metabolic Activation
Synergistic Activation
~20 kDa
~52 kDa
~38 kDa
C-caspase 1
GSDMD
GAPDH
F
C-caspase 1
GSDMD
Control
Pyroptosis Model
Glycine Protection
Metabolic Activation
Synergistic Activation
G
Membrane potential ratio (490/505nm)
P = 0.0207
P = 0.0189
P = 0.0112
P = 0.0045
FI490/FI505
Control
Pyroptosis Model
Glycine Intervention
GlyR Antagonist
Membrane Repair Blockade
H
Control
Pyroptosis Model
Glycine Intervention
~48 kDa
~38 kDa
GlyR a1
GAPDH
Control
Pyroptosis Model
Glycine Intervention
GlyR Antagonist
Membrane Repair Blockade
~237 kDa
~38 kDa
Dysferlin
GAPDH
GlyR a1
Dysferlin
Control
Pyroptosis Model
Glycine Intervention
GlyR Antagonist
Membrane Repair Blockade

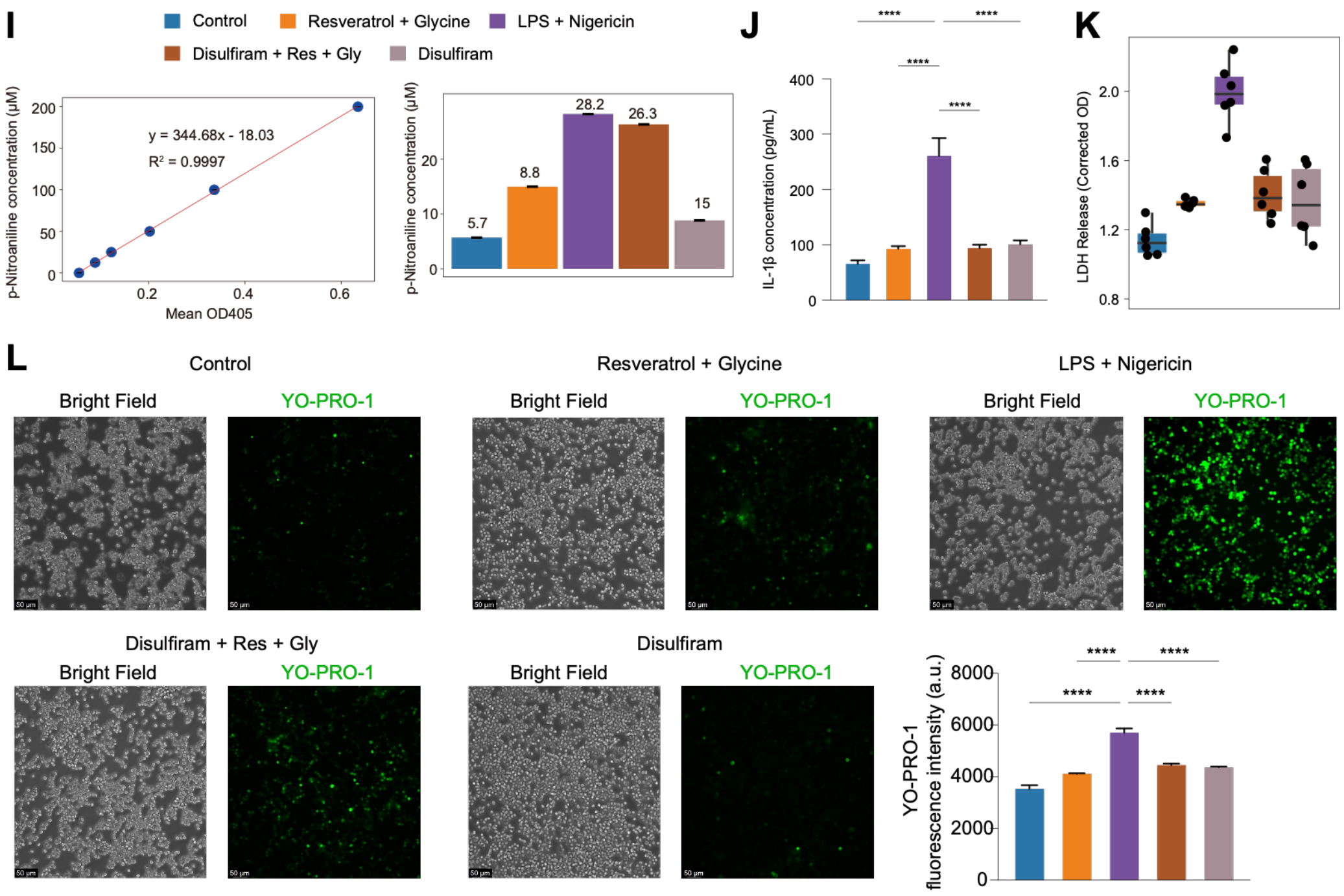
I
Control
Resveratrol + Glycine
LPS + Nigericin
Disulfiram + Res + Gly
Disulfiram
p-Nitroaniline concentration (μM)
y = 344.68x - 18.03
R² = 0.9997
Mean OD405
5.7
8.8
28.2
26.3
15
J
IL-1β concentration (pg/mL)
****
K
LDH Release (Corrected OD)
L
Control
Resveratrol + Glycine
LPS + Nigericin
Bright Field
YO-PRO-1
Disulfiram + Res + Gly
Disulfiram
YO-PRO-1
fluorescence intensity (a.u.)

Supplementary Figure 5. In vitro validation of macrophage activation and Vita-field mechanism

(A) LDH release assay across five treatment groups (n=3).

(B) CCK-8 viability assay (n=3).

(C) ELISA quantification of IL-1β, IL-6, and TNF-α secretion (n=3).

(D) Flow cytometric analysis of Annexin V/PI staining, with percentages of viable (Q4), early apoptotic (Q3), and necrotic/late apoptotic (Q2+Q1) cells indicated (n=3).

(E) Western blot analysis of full-length GSDMD (GSDMD-FL), GSDMD N-terminal cleavage fragment (GSDMD-NT), CASPASE-1, and β-ACTIN.

(F) Quantification of GSDMD-NT/GSDMD-FL ratios from (E) (n=3).

(G) $DiBAC_4$ (3) fluorescence intensity representing membrane potential across indicated groups (n=3).

(H) Western blot analysis of DYSFERLIN, GLYRα1, and β-ACTIN. Data are presented as mean ± SD. Statistical significance was determined by one-way ANOVA; *P < 0.05, **P < 0.01, ***P < 0.001; ns, not significant (n=3).

(I) Calibration curve for p-nitroaniline quantification (left) and Caspase-1 activity (right) measured across Control, Resveratrol+Glycine (Res+Gly), LPS+Nigericin (LPS/Nig), Disulfiram+Res+Gly, and Disulfiram groups.

(J) ELISA quantification of mature IL-1β secretion.

(K) LDH release assay showing plasma membrane rupture.

(L) YO-PRO-1 uptake assay demonstrating GSDMD pore formation.

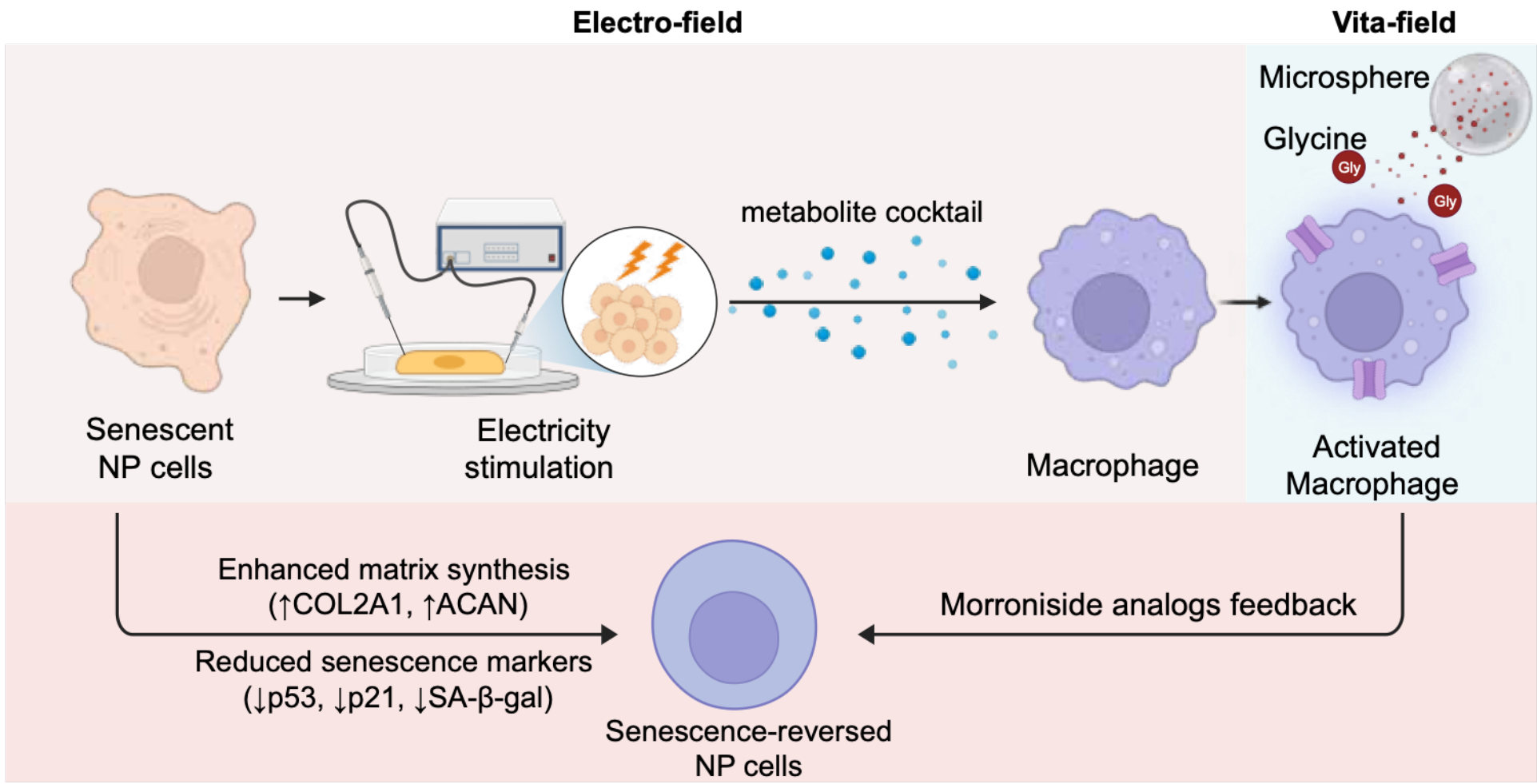

Supplementary Figure 6. Mechanistic coupling of Electro-field and Vita-field in NP cell rejuvenation

Electro-Vita dual-field axis: Electro-field reprograms NP cell metabolism to reverse senescence and induce metabolite secretion, while Vita-field provides glycine-mediated cytoprotection that sustains macrophage activation and enables prolonged morroniside analogs feedback to NP cells.

Actin	AGGTCGGTGTGAACGGATTTG	TGTAGACCATGTAGTTGAGGTCA
p53	CCCTCTGAGCCAGGAGACATT	CCCAGGTGGAAGCCATAGTTG
p21	CTTGTCGCTGTCTTGCACTCTG	GAAATCTGTCAGGCTGGTCTGC
Cdkn2a (p16INK4A)	CGGGGACATCAAGACATCGT	AAGAAGAAAAAGGCGGGCTG
Taf13	GTATGATGTATGGGTTTGGGGAC	CTAGCCCGTTTCAATTCTTCAT
Bnip3	TTCCAGCCTCCGTCTCTATTTA	AATCTTCCTCAGACAGAGTGCTG
Taz	GGACCAAGTACATGAACCACCTC	GTTCCAGATGTGGCGGAGTTT
Ctgf	CCTACCGACTGGAAGACACATTT	TTGTCATTGGTAACTCGGGTG
Ccn1 (Cyr61)	CAGTGCTGTGAAGAGTGGGTTT	CAAAGACAGGAAGCCTCTTCAGT
Ankrd1	GTGGATGTGCCGAGGTTTCT	GTGGAGTGCCGTCCGTTTAT

Supplementary Figure 7. Primer sequences used for quantitative real-time PCR.

Term	Definition
Dual-Fields	Refers to the spatiotemporal coupling of the physical Electro-field and the biochemical Vita-field. This coupling is achieved through the ultrasound-responsive controlled-release properties of the microneedle-microsphere system----ultrasound simultaneously triggers the PLLA component to generate electrical stimulation (Electro-field) and promotes the release of glycine from the microspheres (Vita-field), thereby achieving temporal synchronicity and spatial overlap of these two distinct types of signals at the lesion site.
Electro-field	Refers to a localized, non-invasive physiological-range electrical field generated by ultrasound-triggered piezoelectric PLLA films and delivered locally to the nucleus pulposus region of the degenerated intervertebral disc via the conductive microneedle. The stimulation intensity is set at 2 mA.
Vita-field	Refers to a protective biochemical microenvironment formed by the sustained release of glycine from liposomes in the local lesion site. Our experimental results demonstrate that glycine release maintains macrophage viability and functionality upon GSDMD pore formation, thereby prolonging the secretion of reparative factors. We conceptualize this protective effect as the Vita-field to describe its "membrane-protective effect" in intercellular communication.
Electro-immunometabolic axis	Refers to a complete bidirectional closed-loop signaling cascade: external electrical stimulation induces metabolic reprogramming of senescent nucleus pulposus (NP) cells, leading to the secretion of seven specific immunomodulatory metabolites (the metabolite cocktail). These metabolites drive non-pyroptotic GSDMD-dependent hyperactivation of macrophages, which in turn secrete morroniside-like metabolite. Morroniside-like metabolite further promotes NP cell rejuvenation and functional recovery through activation of the Hippo-YAP/TAZ pathway.
Metabolite Cocktail	Refers to a panel of seven specific metabolites actively secreted by senescent NP cells into the microenvironment following electrical stimulation-induced metabolic reprogramming. The cocktail comprises: leucine, proline, lysine, carnitine, N-acetyl-L-methionine, N-acetyl-D-glucosamine, and

	resveratrol. This cocktail serves as the first messenger signal that initiates NP-macrophage communication.
Morroniside-like metabolite-mediated feedback loop	Refers to the closed-loop regulatory mechanism in which hyperactivated macrophages secrete morroniside-like metabolite, which then acts on senescent NP cells by suppressing the phosphorylation of the core Hippo pathway kinases MST1/2 and LATS1/2, thereby activating downstream effectors YAP/TAZ and their target genes (CTGF, CYR61, ANKRD1). This cascade inhibits NP cell apoptosis, promotes matrix synthesis, and reverses the senescent phenotype.
Chinese Microneedle	The localized lesion-delivery device used in this study. It is characterized by a minimally invasive design inspired by traditional acupuncture principles, featuring helical grooves machined at the needle tip that physically anchor functionalized microspheres via a hydrogel adhesive, enabling precise, quantitative, and low-trauma delivery of microspheres to the nucleus pulposus region of the degenerated intervertebral disc. The threaded structure effectively prevents premature shedding of microspheres during tissue penetration.
Cell communication channel	A functional bidirectional paracrine signaling loop between NP cells and macrophages, mediated by soluble metabolites and cytokines, that enables sustained intercellular crosstalk.

Supplementary Figure 8. Key Terms and Definitions

Experiment	Species	Age	Cell type	Source	Passage
Bulk RNA-seq	Mouse (C57BL/6)	N/A	NP cells	Commercially purchased	Treated with D-galactose (8 mg/mL) for 7 days and passaged three times prior to experimentation
Single-cell RNA-seq	Rat (SD)	8 weeks and 11 months	Disc tissues	Isolated in-house	N/A
Histological staining	Rat (SD)	11 months	Disc tissues	Isolated in-house	N/A
In vivo experiments	Rat (SD)	11 months	Disc tissues	Isolated in-house	N/A
Co-culture experiments	Mouse (C57BL/6)	N/A	NP cells + Macrophages	Commercially purchased	Treated with D-galactose (8 mg/mL) for 7 days and passaged three times prior to experimentation

Supplementary Figure 9. Summary of sample sources and reference genomes used in this study.

Group	Description	n (imaging)	n (histology)	Sex	Age	Disc level per animal	Time points	Outcome measures
Aging	11-month-old untreated rats (age-matched control for in vivo)	3	-	Male	11 Months	One disc per animal	0, 2, 4, 6, 8 weeks	X ray DHI
Lesion	11-month-old + needle puncture with rotation (degeneration model)	3	-					
ES	Lesion + electrical stimulation (single treatment)	3	-					
Sham	11-month-old + needle insertion without rotation (sham control, MRI only)	3	-					MRI T2 signal intensity
Control	11-month-old untreated rats	3	-					
Dual-regulation	Lesion + microspheres + ES + glycine (combined treatment)	3	6				0, 2, 4, 6, 8 weeks, 8 weeks	X ray DHI, MRI T2 signal intensity, Histology (GAG,
Aging damage group	11-month-old + needle puncture with rotation (degeneration model)	-	6				8 weeks (endpoint)	Histology (GAG, AGG, COL II, scoring)
Blank microsphere	Lesion + blank microspheres (material control)	-	6					
Glycine only group	Lesion + glycine only (glycine control)	-	6					
Healthy group	11-month-old untreated rats	-	6					

Supplementary Figure 10: Experimental design for in vivo studies.

Note: This table provides a direct cross-reference between the names as they appear in the main text and figure legends and the standardized group names used in the experimental design. The “Control” group in Fig. 7B refers to 11-month-old untreated rats used as the normalization baseline for MRI signal intensity (set to 1). The “Healthy group” in Fig. 7C-F refers to the same 11-month-old untreated rats, shown as a reference for normal disc histology. The “Aging damage group” is synonymous with the “Lesion” group. The “Blank microsphere control group” and “Glycine only groups” correspond to the Microsphere and Glycine groups, respectively. This naming convention is used consistently throughout the main text and figure legends to avoid ambiguity.

Supplementary Table 1

Cluster 1		Cluster 2	
NAME	MEM.SHIP	NAME	MEM.SHIP
Akr1b1	0.62408156114306	Hoxa4	0.742972154618108
Apoe	0.598432172874351	Folh1	0.804423525907543
Il11	0.615025256988173	Cd36	0.778244274136603
Ggt1	0.51565939010464	Eno2	0.854761163773759
Cd40	0.630638598338117	Chrd	0.616132260939843
Arg1	0.505363217630365	Pcdha10	0.594196800906794
Moxd1	0.681122812912413	Hapln4	0.754173467141565
Gria1	0.749924148059721	Cers4	0.637183767271329
Lratd1	0.563361507383132	Eya4	0.744288649497951
Tmem132e	0.562983954498296	Ebf3	0.907762206139609
Gcnt2	0.519508697525076	Mcoln2	0.706501728720964
S100z	0.687631972734017	Alx3	0.837845679517979
Sla	0.750372016353586	Cybb	0.62955094983078
Fam83f	0.67184902378841	Notch4	0.82901033756671
Abcg1	0.538483381563605	Plekho1	0.835915388174984
Clip4	0.571358771286332	Lcorl	0.70692540707124
Ifitm1	0.566476912217615	H2-M3	0.600915515619832
Lamb3	0.549786110280101	Wt1	0.717756443298866
Chrna1	0.746039520317079	Plxdc1	0.504934938417997
Bmp2	0.532456237825987	Pltp	0.874665196412682
Sdcbp2	0.516838708613541	Ptgis	0.56432731218518
Tm4sf4	0.746259857727682	Gpx3	0.759783841668414
Coro2a	0.513612211090929	Frk	0.819994562139874
Plin2	0.736843667202702	Kitl	0.693854993070548
Prom1	0.741486525455812	Fam241b	0.590887683477228
Sorcs2	0.664474936732326	Plek	0.773142427968867
Pagr1a	0.65755486848302	Aldh1l2	0.698913443991384
Trim66	0.532950758364218	Pimreg	0.56521153731609
Ano1	0.624249527006505	Foxg1	0.642863627839575
Chst2	0.607556699696244	Sav1	0.662193320206106
Tmem59l	0.560743241866628	Smoc1	0.593455666558243
P2ry13	0.538690259068535	Acot2	0.735385848053887
Acp7	0.687704305888573	Fdft1	0.742716154234757
Tbkbp1	0.586847096828518	Gpr137b	0.674634070378247
Slco4a1	0.566725194254852	Aspn	0.857737962316499
Cpa4	0.503035797441441	Cenpp	0.86034300634388
Cdh6	0.551787016519957	Pcsk1	0.865918349724839
Abcb1a	0.614547691618272	F2rl2	0.516085292419555
Map7d2	0.736412508500276	Esco2	0.59928942914569
Chd7	0.621858939639286	Ephx2	0.875897975385949
Gjb3	0.579716716751516	Nefm	0.623554091026955
Ttc9	0.735290381522059	Npr3	0.70724326996371
Lgr6	0.705160058633078	Sqle	0.832955004283906
Nrep	0.595117461193308	Mtss1	0.904979060284117
Sh2d5	0.635441693848642	Syngr1	0.594699259039159
Penk	0.708901952603005	Septin3	0.534003565258385
Fut4	0.723286542342616	Cela1	0.745971257961658

Lce1h	0.745865853021484
BC030500	0.69998948590388
Lrrc15	0.555062397486496
Tg	0.560239369437231
Gper1	0.521989374613623
Gpr50	0.505494558909903
Sp7	0.707583404637743
Or2t1	0.567277340616282
Trbc2	0.747812523221488
Gm13285	0.526409936803496
Samd11	0.658142546105751
Sh2d1b1	0.678511278709633
Gm49361	0.748472644135145

Cluster 3

NAME	MEM.SHIP
Tbx4	0.539295375644474
Acvrl1	0.777594105826411
Hoxa10	0.728977955561796
Irx2	0.546589489915207
Hoxc13	0.623324254611313
Hoxc11	0.781625155182715
Hoxd13	0.533667304122248
Crabp2	0.500391974457712
Crip2	0.523682776998668
Hyal1	0.633531880792045
Fcrl2	0.565113482723411
Cd34	0.584177773970895
Mmp9	0.674848304580487
6330403K07Rik	0.583157885489233
Sema6a	0.591502711214698

Clic5	0.572338421075203
Qpct	0.725224346786649
Celf4	0.619259102556403
Rab27b	0.890965298393916
Gna14	0.857017759811422
Ltbp3	0.883410848441583
Vegfb	0.733053701321627
Sntg1	0.509621968124125
Gsta3	0.661212494805554
Cd28	0.580167256910673
Cdk15	0.868637233392214
Ecel1	0.694513166304337
Niban1	0.557547436363497
Selp	0.668447440926276
Fcna	0.810498318577133
Sord	0.872956678738604
Knl1	0.898719799072543
Lrrc39	0.699689815510363
Bdh2	0.893308330282125
Atp6v0d2	0.687293738855785
Cdkn2c	0.616378724401159
Cda	0.557151470026752
Sdsl	0.908705818625775
Tgfa	0.757254481018013
Slc41a3	0.770988323413984
Mgp	0.616518588361743
Irak1	0.907223122152705
Eomes	0.673438008697936
Cck	0.691133573422244
Trib3	0.884099895909044
Igdcc4	0.835051779394272
Ank2	0.839876288870381
Lpcat2	0.738458739560803
Akr1c14	0.698830148828392
Rhov	0.763675838502949
Jdp2	0.648914227456959
Fbxo45	0.516287758813661
Gadd45a	0.671578935737569
Kif15	0.616896732515547
Tgm2	0.574104730156358
Atp13a4	0.536727520290172
Hoxa5	0.638043349822762
C1s1	0.601927972825852
1700003F12Rik	0.750961991421678
Hoxb7	0.654448483027497
Ccdc68	0.726002936268325
Plcl2	0.593078231669274
Rpl22l1	0.571249082060104
Isg20	0.862231954820543

Ptprb	0.528964387510248	Foxk2	0.661890526979098
Klhl29	0.52185991656167	Rcan2	0.539686144936876
Coch	0.587257500168784	Plcb4	0.893794474172887
Bdkrb2	0.524556062261616	Fmo2	0.777122777054025
Nedd9	0.725807417773489	C3ar1	0.74256185401458
Barx1	0.664128701121044	Slc26a7	0.865071909508533
Pitx1	0.693493944654882	Ildr2	0.545303238307044
Anxa8	0.696542197183056	Pck2	0.688449795489671
Adcy4	0.583331360109697	Pif1	0.755441855604484
Sybu	0.684067937526557	Nacad	0.517068246831064
Cldn1	0.624056311819175	Car8	0.679452298205542
Il1rap	0.570151943233486	Fbxo2	0.771972589437397
B3gnt5	0.564746476959865	Coro2b	0.635446166363913
Masp1	0.596153110350566	Pgm5	0.704450319954097
Pla2g7	0.614217066095344	Bex2	0.553042080652761
Arhgap28	0.731846081518891	Klhl6	0.617499979510013
C2	0.705394012362057	Ifi209	0.565053383526576
Pcsk5	0.753586498531147	Cdc25c	0.553342427623202
Lipo3	0.781553567343342	Ctla2a	0.593040049236237
Bnc1	0.594770250872036	Snhg11	0.701859367531802
Igfbp5	0.762992579631069	Chrm3	0.618615193004881
Kif26b	0.698954293229935	Rbp1	0.608892826040987
Kif5c	0.653326660666274	Pcdhb16	0.692681630727742
Pfkfb3	0.604182293755704	Pcdhb18	0.720114032867613
Abcb11	0.677983163611814	Gpr137c	0.675254747871743
Dhrs9	0.628564218669902	Themis	0.618479517763127
Itga6	0.793854786054058	Upk1b	0.700873656500309
Syt13	0.603292578377251	Ccdc69	0.604384868163541
Angpt4	0.519177936067701	Mmp12	0.907395198325263
Mecom	0.79094296403586	Foxc1	0.749169681184238
Tbx15	0.525276548502515	Samd3	0.743004768219638
Prss12	0.778320108248663	Rab7b	0.605568574139917
Dkk2	0.657336800711969	Creb5	0.748092645071383
Bcar3	0.609518404293877	Gpr141	0.637311993375015
Tnc	0.650581284416858	Aldh1a1	0.826620089588778
Mfsd2a	0.794874074462568	Sytl5	0.650448557138412
U90926	0.7791532162608	Xlr	0.72437808780593
Erp27	0.533744611198364	Hmgb2	0.778433087188856
Arhgdib	0.704894221780497	Afp	0.571270674041405
Stk26	0.532904746471312	Prl2c5	0.560577491795291
Itm2a	0.728484273853785	Prl2c3	0.627723050326943
Gpm6b	0.767553146701287	Tmem140	0.598293795423014
Tktl1	0.576883594631485	Serpinb9g	0.561757921127817
Irx5	0.629638727909195	Tcp11x2	0.754591281168918
Slc27a1	0.747629425007635	Idi1	0.863409463672403
Ankk1	0.784031932601553	Znrf2	0.541640767707772
Crabp1	0.698733251815479	Rad51b	0.686326840116096
Clstn2	0.586214671380015	Fcgr4	0.649224896058598
Mgll	0.755251766389064	Pyroxd2	0.829940467701732

Mamdc2	0.781145134555339	Kcnd2	0.775838908387778
Rtp4	0.70439435222115	Galnt13	0.626056822579199
Otud7a	0.561153996148689	Cxcl12	0.673495586690922
Kcnk10	0.714391715398878	Klra6	0.767605127657502
Vax2	0.582696662075669	Serpinb9e	0.515073538285872
Gdpd5	0.558996956083041	Fabp4	0.873162660676308
Slc2a13	0.760194304059163	Tubb4a	0.768320248490435
Unc93b1	0.707586055027508	Kcnj15	0.839973802874511
Apln	0.755677529824352	Erhrt-ps	0.535587090249442
Aff3	0.526773005286622	Nmral1	0.895399445558481
Chst7	0.724747900794442	Tnfrsf11b	0.678214342274634
Hoxa11	0.776998515915277	Srrm4	0.556345448534368
Spats2l	0.691742623460598	Hvcn1	0.71311202487276
Rpl39l	0.623798950162971	Tnfsf18	0.666035100414833
Fgl2	0.652103053119845	Kpna2rt	0.656406122345513
Npnt	0.680341729187302	Wfdc17	0.565095413275188
Macc1	0.516656273427976	Sp140	0.68611353046715
Klhdc8a	0.516071778791224	Zfp1008	0.812114316118725
Fam83g	0.683258938655775	Gpr174	0.63906569501481
Mfap4	0.689543849771424	Xlr3b	0.701721704982367
Mustn1	0.681944872451692	Ifi204	0.719984266001653
Tafa2	0.517234072481444	Ifi207	0.825172637257918
Pcsk9	0.580447645967133	Adh1	0.524418629342557
S1pr1	0.761096925179194	Pira12	0.750591135944488
Dcaf12l1	0.64633295212064	Malrd1	0.882158554331011
Tnfrsf26	0.755013321992987	Smim1	0.564429474474031
Kctd4	0.527947265181274	Fyb2	0.822242732318092
Sfn	0.541832757625049	Zfp1004	0.752321689706037
Palm3	0.579819472385823	Prl2c2	0.65480311271452
Igf2	0.599387573594344	Col4a3	0.62364929954793
Gal3st1	0.5115548610912	H2-DMb1	0.733922192938562
Grem2	0.596306142349335	Hoxa3	0.617770390428904
Hoxd10	0.607558428066002	Klra4	0.708700745465342
Hs3st1	0.596929812097294	Alkal1	0.871958154985394
Flrt3	0.781969846217547	Pira2	0.764206401189627
Tafa5	0.555701705124869	Gm2004	0.560881902116497
Mab21l1	0.788376772446891	Vmn2r26	0.611227813441375
Ebf1	0.503206350238389	Pcdhga1	0.717398014741162
Mab21l2	0.633844990298435	Pcdhgb5	0.906424399701757
Klk8	0.555169284384718	Pcdhga8	0.799677506617347
Adamtsl3	0.715801892026602	Gbp5	0.907652225462887
Hpcal1	0.643901837534083	Gm49391	0.766081800712662
-	0.600416510722999	Gm49392	0.564720949346748

	**Cluster 4**	Sema3f	0.553418413035238
NAME	MEM.SHIP	Ripor2	0.735390895661713
Col6a1	0.657888143768694	Wnt4	0.649722227734069
Cfp	0.610794004368924	Prr32	0.703741906409204
Aqp1	0.636950574508686	Icam1	0.567422903654783

Slc1a3	0.515186457882255	Vnn1	0.569949218614947
Jph2	0.593655175923075	Hand2	0.638290506346234
Tbx5	0.720556874896286	Igfbp2	0.614701891158284
Myh11	0.517599430404264	Kazn	0.645357754148185
Plagl1	0.602065471256312	Ramp3	0.587566695638934
Lama4	0.541103496128239	Fmod	0.592413687266002
Ntn4	0.62688635540131	Pm20d1	0.735640353373732
Efemp1	0.581951073057987	Synpo	0.708943884304311
Krt19	0.508332657985964	Cavin2	0.73276856722277
Id4	0.533452344457008	Kcne4	0.690888869167846
Cxcl14	0.724678078492233	Tshz2	0.727519066868056
Nid2	0.608936981863797	Ahcyl	0.509235018983319
Nr4a1	0.646263648349168	Lurap1l	0.702994934478657
Sncg	0.734733091163015	Vstm2a	0.561224640216996
Dpysl4	0.732937573999244	Epha3	0.61115750071725
St8sia2	0.581270366821704	Adh7	0.724260419620258
Ttll11	0.680179437132536	Uts2b	0.640329520938053
Zdbf2	0.71683350138047	Cend1	0.693679924374271
Slc27a3	0.678345395650083	Kdr	0.517497653042919
Tinagl1	0.566206332367548	Ifi27	0.603564446506181
Cxcl5	0.533367726133675	Capn6	0.552066979295419
Tmem176b	0.556747552316753	Ptprz1	0.624989038592113
Fgf13	0.683652667186274	Hs3st3b1	0.654546907903075
Tnmd	0.675255191400726	Rasgrp3	0.609988667893077
Crispld2	0.701148775181795	Fndc1	0.511959695118076
Usp2	0.563870442236012	Plac9	0.64224550610984
Car12	0.510991838624262	Ctxnd1	0.73048875179316

Supplementary Table 2

Cluster 1

NAME	MEM.SHIP
M351T27_NEG	0.525360480703824
M158T224_NEG	0.667933775064257
M177T543_NEG	0.724221608165289
M281T478_NEG	0.730826880514211
M187T26_1_NEG	0.761775569577539
M401T227_NEG	0.72701830815976
M513T384_NEG	0.741171613924036
M205T211_NEG	0.622220887259015
M242T414_NEG	0.547656590485639
M313T217_NEG	0.647527237287085
M839T449_NEG	0.653917973281729
M482T248_NEG	0.73129368091594

Cluster 2

NAME	MEM.SHIP
M231T25_NEG	0.553884238143648
M753T264_NEG	0.811280273006336
M337T27_1_NEG	0.551850740478725
M203T26_1_NEG	0.580209164174516
M239T47_NEG	0.786085335215361
M293T584_NEG	0.539074525783565
M291T26_NEG	0.814666699053682
M185T45_NEG	0.798204341561543
M565T436_NEG	0.681752331200592
M150T476_NEG	0.738994295127423
M170T479_2_NEG	0.60567143297835
M328T236_NEG	0.751454261311185
M229T29_NEG	0.561454590399088
M355T31_NEG	0.814592013193028
M252T479_NEG	0.687720119983272

Cluster 3

NAME	MEM.SHIP
M469T521_NEG	0.88320639039791
M125T458_NEG	0.871872315868836
M85T457_NEG	0.841480490281312
M238T472_NEG	0.518526915746601
M543T273_NEG	0.550998379274703
M191T584_NEG	0.739038927728742
M113T458_NEG	0.713632429564488
M101T457_NEG	0.805870516367951
M149T385_NEG	0.726338909741272
M161T457_NEG	0.849402872189382
M89T443_NEG	0.577156812806893
M325T32_NEG	0.83561946562251
M142T428_NEG	0.767284684141743
M221T206_NEG	0.617428801720129
M129T46_NEG	0.512245694161994
M101T443_NEG	0.5355938933985
M161T211_NEG	0.70171801868734
M218T429_NEG	0.579020688630923
M360T433_NEG	0.746336080862594
M297T35_NEG	0.849069361378977
M292T458_NEG	0.835014169336088
M329T519_NEG	0.619846778083293
M97T542_1_NEG	0.648005140065522
M71T457_NEG	0.858660420920983
M354T500_NEG	0.545262921099668
M311T34_NEG	0.821168874818423

Cluster 4

NAME	MEM.SHIP
M427T460_NEG	0.582874371018825
M362T437_NEG	0.662987352861789
M328T416_NEG	0.628156799534396
M145T680_2_NEG	0.643759092684533
M280T437_NEG	0.67757726333012
M192T357_2_NEG	0.515481260916729
M526T437_NEG	0.606891949964569

Cluster 5

NAME	MEM.SHIP
M298T472_NEG	0.779398811587525
M462T472_NEG	0.77778964761678
M216T471_NEG	0.73111738880772
M157T319_NEG	0.51744548298666

Cluster 6

NAME	MEM.SHIP
M147T247_NEG	0.711621429796161
M388T451_NEG	0.665280844741893
M210T256_NEG	0.741538636230161
M233T280_2_NEG	0.504290527177153
M233T280_1_NEG	0.511438949256066
M408T441_NEG	0.66466802273127
M193T267_NEG	0.660708758094181
M259T77_NEG	0.858358232158513
M210T454_NEG	0.726222601886865
M342T389_NEG	0.593442928027957
M310T459_NEG	0.812112647643534
M217T397_NEG	0.843313995200106

M293T385_NEG	0.509736667835594	M278T464_NEG	0.608542101521316
M473T496_NEG	0.527737313069805	M147T389_NEG	0.543753122497119
M380T471_NEG	0.757690255167867	M171T44_NEG	0.767036190036093
M114T319_NEG	0.711654126590311	M225T400_NEG	0.825901761298054
M376T440_NEG	0.549866375096512	M87T240_NEG	0.83456933280037
M255T306_NEG	0.559451612111854	M299T398_NEG	0.591598350639326
M176T216_NEG	0.560519947973006	M665T625_NEG	0.761779725802687
M333T306_NEG	0.60138565866457	M341T498_NEG	0.679104933502536
M151T394_1_NEG	0.712898111927453	M371T45_NEG	0.512440984973061
M201T425_NEG	0.777411688002808	M241T383_NEG	0.695408331446974

Supplementary Table 3

ID	MN1	MN2	MN3	MES1	MES2
M333T174_neg_NEG	2013405	1591417	1552971	2869923	3204774
M289T303_neg_NEG	721659.6	956698.8	966225.3	1648680	1854711
M333T66_neg_NEG	349001.6	455470.2	400705.6	891802	943214.3
M753T134_neg_NEG	502878.3	831835.9	641719.1	951599	1091670
M277T274_neg_NEG	66518.71	263305	134923.6	328877	452069
M345T119_neg_NEG	338814.3	294575.7	441981.4	651312	587621.5
M333T53_neg_NEG	22688.1	16407.94	15636.52	153867	147401.1
M704T129_neg_NEG	372.0332	1209.101	527.4958	41021.5	158721.4
M317T280_neg_NEG	145736	206731.1	176001.8	331887	251036.7
M260T311_neg_NEG	239848.5	195916.4	238247	144566	89832.01
M398T238_neg_NEG	65098.31	56886.7	62129.1	236739	142053.1
M383T312_neg_NEG	157968.4	88328.1	88572.74	6411.82	7076.826

M337T286_neg_NEG	8967.877	17109.76	20076.43	138053	78152.18
M355T303_1_neg_NEG	159206.5	125020.7	197053.8	266400	244383.4
M310T209_neg_NEG	78816.39	93959.24	82775.45	16206.1	5434.876
M721T132_neg_NEG	47995.63	128907.1	45612.35	157219	148462.1
M757T154_neg_NEG	177.14	72.47744	409.2881	26716.3	84713.98
M626T310_neg_NEG	20081.68	48065.99	33620.19	86318.5	87592.77
M891T469_neg_NEG	37066.22	64829.38	55470.67	121111	146611.1
M602T173_neg_NEG	1406.91	2928.251	2494.731	22250.6	77623.98
M398T127_neg_NEG	607.9643	1128.94	763.4672	20288.7	76777.78
M321T160_neg_NEG	85089.06	101523.7	148092.4	189101	160232
M801T158_neg_NEG	767.004	153.8973	319.1982	23042.8	75704.86

M511T101_neg_NEG	13593.91	24088.28	15712.5	65646.8	58231.74
M431T303_neg_NEG	64406.48	74671.91	61846.23	112070	124230.5
M498T158_neg_NEG	2842.379	3294.058	3940.458	68029.8	30817.67
M337T192_neg_NEG	42057.08	59166.96	53481.59	85866.7	94797.65
M451T315_neg_NEG	104587.4	82609.73	85626.73	67526.1	25754.25
M511T315_neg_NEG	7006.422	6622.709	11291.83	33178.4	54211.03
M158T142_neg_NEG	63849.55	62385.78	40939.9	89233.6	93245.92
M365T36_neg_NEG	53818.33	75075.37	70419.01	24001.8	44825.96
M271T204_neg_NEG	18615.37	35215.33	20026.38	50432.7	71807.72
M235T67_neg_NEG	188.371	392.503	2074.446	15969.3	50030.36
M699T303_2_neg_NEG	24176.8	29379.79	27563.51	58658.1	54149.77

MES3	control_mean	case_mean	log2_FC(case_mean/control_mean)	Pvalue	FDR
3304857	1719264.4	3126518	0.862765451	0.002055	0.358961
2000827	881527.9139	1834739	1.057497057	0.001827	0.355697
617621.8	401725.8134	817545.9	1.025088638	0.017008	0.50422
1037565	658811.1132	1026945	0.64042154	0.0238	0.563839
454837.4	154915.753	411927.7	1.410907186	0.022438	0.556602
487804	358457.1424	575579.3	0.683214003	0.028259	0.595519
130272.8	18244.18624	143847	2.979026323	7.01E-05	0.186965
163681.9	702.8766226	121141.6	7.429206856	0.039766	0.632141
306900.8	176156.3166	296608.1	0.751701686	0.01538	0.491268
127139.8	224670.6674	120512.6	-0.898627899	0.008541	0.459447
145201.8	61371.36648	174664.6	1.508949479	0.022007	0.550694
7782.558	111623.097	7090.402	-3.976624262	0.010737	0.474227

118710.6	15384.69002	111638.5	2.859267622	0.005848	0.451346
261193.1	160427.023	257325.6	0.681678075	0.011359	0.477541
13344.71	85183.68999	11661.9	-2.868774448	0.000189	0.186965
149967.2	74171.68423	151882.8	1.034018045	0.047591	0.645759
73653.97	219.635166	61694.75	8.133894749	0.025869	0.587927
93161.5	33922.61868	89024.25	1.391950873	0.00273	0.358961
82342.99	52455.42162	116688.2	1.153495299	0.034487	0.623389
68461.12	2276.630656	56111.89	4.623334357	0.034829	0.62459
64373.49	833.4573223	53813.32	6.012711045	0.036546	0.62459
170377.3	111568.394	173236.8	0.634816709	0.040635	0.632141
57070.87	413.366513	51939.52	6.973267231	0.028785	0.598231

67995.85	17798.2279	63958.13	1.845394074	0.000446	0.216887
99483.77	66974.87436	111928.2	0.740881381	0.00527	0.44
46267.67	3358.964855	48371.73	3.848075438	0.014048	0.488167
95333.92	51568.5447	91999.42	0.83513342	0.002364	0.358961
47047.34	90941.28408	46775.88	-0.959170528	0.033495	0.622435
52750.63	8306.986382	46713.35	2.491437882	0.005221	0.44
95915.59	55725.07568	92798.38	0.73577292	0.008382	0.459447
20490.37	66437.5717	29772.72	-1.158008224	0.021214	0.54841
57784.88	24619.02626	60008.43	1.285391488	0.012586	0.482359
41180.32	885.106555	35726.67	5.335006485	0.027058	0.592404
61555.86	27040.03184	58121.23	1.103968365	0.000298	0.199942

VIP	MS2_name	RT	m/z	score
7.002585	5-[4-(4-Hydroxy-2-quinazolinyl)phenyl]-5-methyl-2,4-imidazolidinedione	174.2	333.0912	0.0064
5.664393	11-Methoxycurvularin	303.3405	289.1016	0.0147
3.513257	Phlorin	65.7185	333.0906	0.017
3.322245	1,2-disinapoyl diglucoside	134.0125	753.2244	0.9679
2.813092	2,3,7,9-Tetrahydroxy-4a-methyl-3,4-dihydro-2H-benzo[c]chromen-6-one	274.314	277.0736	0.0774
2.449588	1,7-Bis(3,4-dihydroxyphenyl)-6-hydroxyheptan-3-one	119.2285	345.1256	0.2667
2.074847	Aigialomycin D	53.248	333.1367	0.5413
1.926153	Fumonisin B2	128.885	704.3692	0.0012
1.903064	N-Phenylacetyl-L-prolylglycine ethyl ester	280.457	317.1472	0.01
1.840406	2,3-Dimethoxy-5-sulfamoylbenzoic acid	311.315	260.0232	0.0968
1.810851	6-Bromo-2-[3-(2-methylpropoxy)phenyl]quinoline-4-carboxylic acid	238.0215	398.0333	0.0496
1.779688	Maltotetraose	312.305	383.1211	0.0494

1.747654	3-O-p-Coumaroylquinic acid	285.926	337.1006	0.8413
1.723529	5-(2-Chlorophenyl)-7-fluoro-8-methoxy-3-methyl-2,10-dihydropyrazolo[3,4-b][1,4]benzodiazepine	303.046	355.0722	0.0105
1.594954	Ethyl 4-(2-chloro-6-fluorophenyl)-2-methyl-6-oxo-1,4,5,6-tetrahydropyridine-3-carboxylate	208.96	310.0697	0
1.481548	Specnuezhenide	131.957	721.1973	0.8355
1.393778	Cyclo(D-alanyl-D-tryptophyl-4-aminobutanoyl-D-alanyl-D-valyl-L-phenylalanyl-D-valyl)	153.915	757.3998	0.0001
1.346701	5-(Diethoxyphosphoryl)-5-methyl-1-pyrroline-N-oxide-biotin	310.056	626.2373	0.0007
1.34009	Cytidine 5'-diphosphate ethanolamine	469.472	891.1164	0
1.290515	Phosphatidylcholine lyso 22	173.3655	602.3871	0
1.277368	(3z)-3-[[1,6-dimethyl-2-[(1e,3e)-penta-1,3-dienyl]-4a,5,6,7,8,8a-hexahydro-2h-naphthalen-1-yl]-hydroxymethylidene]-5-(1-hydroxyethyl)pyrrolidine-2,4-dione	127.4515	398.2649	0.0011
1.27287	2-Amino-3-[5-tert-butyl-3-(phosphonomethoxy)isoxazol-4-yl]propionic acid	159.761	321.0943	0.0128
1.268115	Antibiotic F 1839A	158.1795	801.4254	0.0091

1.253977	Selaginellin	100.542	511.1602	0.0042
1.206887	Methanesulfonamide, N-[4-[(2S)-3-[[2-(3,4-dichlorophenyl)ethyl]amino]-2-hydroxypropoxy]phenyl]-	302.7205	431.0575	0.0182
1.16596	3- Quinolinecarboxylic acid, 1,4,5,6,7,8-hexahydro-4-(3-hydroxyphenyl)-7-(2-methoxyphenyl)-2-methyl-5-oxo-, 2-methoxyethyl ester	158.116	498.1689	0.0005
1.158498	Altenuene	191.9175	337.0855	0.1834
1.142606	Morroniside	314.902	451.1365	0.0014
1.134195	[(1as,1bs,5s,5as,6s)-6-hydroxy-6-(hydroxymethyl)-2-[[(2r,3r,4s,5s,6r)-3,4,5-trihydroxy-6-(hydroxymethyl)oxan-2-yl]oxymethyl]-1b,5,5a,6a-tetrahydro-1ah-oxireno[3,4]cyclopenta[1,3-d]pyran-5-yl] 3-methylbutanoate	314.598	511.1613	0.0276
1.120159	DL-Indole-3-lactic acid	141.57	158.0614	0.9883
1.073808	15-Cyclohexylpentanorprostaglandin F2.alpha.	35.7775	365.2383	0.0049
1.063754	Butein	203.864	271.059	0.1815
1.044719	3-(2-Methyl-3-oxo-2,5,6,7,8,8a-hexahydro-1H-naphthalen-1-yl)propanoic acid	67.1885	235.1341	0.3261
1.02963	DFLNHc	302.7	699.2328	0.0297

HMDB	CAS	SuperClass	Class	SubClass
--	-	--	--	--
--	-	--	--	--
HMDB0035589	28217-60-9	Organic oxygen compounds	Organooxygen compounds	Carbohydrates and carbohydrate conjugates
HMDB0040838	139891-98-8	--	--	--
--	-	--	--	--
--	-	--	--	--
--	-	--	--	--
HMDB0034703	116355-84-1	Organic acids and derivatives	Carboxylic acids and derivatives	Fumonisins
--	-	--	--	--
--	-	--	--	--
--	-	--	--	--
HMDB0001296	34612-38-9	Organic oxygen compounds	Organooxygen compounds	Carbohydrates and carbohydrate conjugates

--	-	Organic oxygen compounds	Organooxygen compounds	Alcohols and polyols
--	-	--	--	--
--	-	--	--	--
--	-	Organic oxygen compounds	Organooxygen compounds	Carbohydrates and carbohydrate conjugates
--	-	--	--	--
--	-	--	--	--
HMDB0001564	3036-18-8	Nucleosides, nucleotides, and analogues	Pyrimidine nucleotides	Pyrimidine ribonucleotides
--	-	--	--	--
--	-	--	--	--
--	-	--	--	--
--	-	--	--	--

--	-	--	--	--
--	-	--	--	--
--	-	--	--	--
--	-	--	--	--
--	-	Organic oxygen compounds	Organooxygen compounds	Carbohydrates and carbohydrate conjugates
HMDB0035635	64703-85-1	--	--	--
HMDB0000671	7417-65-4	--	--	--
--	-	--	--	--
--	-	--	--	--
--	-	--	--	--
--	-	--	--	--
